# Supplementary material for: Women’s childbirth experiences in the Swedish Post-term Induction Study (SWEPIS): a multicentre, randomised, controlled trial
Source: BMJ Open. 2021 Apr 6;11(4):e042340. doi: 10.1136/bmjopen-2020-042340 (PMC8031013; doi:10.1136/bmjopen-2020-042340)
Supplement: Supplementary data [file bmjopen-2020-042340supp001.pdf]

Supplementary material, Table A Baseline characteristics for participants not answering CEQ2

Variables	Induction group n=125	Expectant management group n=178
Age at randomisation (years)		
Mean (SD)	30.7 (4.8)	30.8 (4.8)
Median (interquartile range)	30 (27; 34)	30 (27; 35)
< 35 years	99 (79.2%)	139 (78.1%)
≥ 35 years	26 (20.8%)	39 (21.9%)
Parity (includes stillborn or live births)		
Primiparous	73 (58.4%)	88 (49.4%)
Multiparous	52 (41.6%)	90 (50.6%)
Smoking at first antenatal visit		
No	96/102 (94.1%)	134/141 (95.0%)
Yes	6/102 (5.9%)	7/141 (5.0%)
BMI at first antenatal visit		
Mean (SD)	25.4 (5.0)	25.7 (5.4)
Median (interquartile range)	23.9 (21.8; 28.3)<br>n=108	24.4 (22.0; 28.0)<br>n=150
Region of birth		
Sweden	84/115 (64.2%)	123/167 (73.7%)
Other Nordic countries	12/115 (10.4&)	12/167 (7.2%)
Europe outside Nordic countries	1/115 (1.0%)	2/167 (1.2%)
Outside Europe	18/115 (15.7%)	12/167 (7.2%)
Highest education		
Primary school ≤ 9 years	5/102 (4.9%)	6/155 (3.9%)
High school 9 to 12 years	37/102 (36.2%)	57/155 (36.8%)
University or corresponding	60/102 (58.8%)	92/155 (59.4%)
Gestational age at delivery (days)		
Mean (SD)	288.7 (1.6)	291.1 (2.6)
Median (interquartile range)	288 (288; 289)	291 (289; 294)
Time from admittance to labour ward to delivery (hours)		
Mean (SD)	21.1 (13.9)	12.8 (10.8)
Median (interquartile range)	18.1 (10.5; 27.8)	10.1 (5.1; 18.0)
Onset of birth process		
Spontaneous	18 (14.4%)	126 (70.(%)
Scheduled caesarean delivery	0 (0%)	1 (0.6%)
Induction	107 (85.6%)	51 (28.7%)
Mode of birth		
Spontaneous vaginal	104 (83.2%)	149 (83.7%)
Instrumental vaginal	7 (5.6%)	12 (6.7%)
Caesarean delivery	14 (11.2%)	17 (9.6%)
Use of epidural anaesthesia	87 (69.6%)	84 (47.2%)
Maternal complications		
Perineal lacerations III-IV	4 (3.2%)	4 (2.2%)
Postpartum haemorrhage (>1000 ml)	16 (12.8%)	14 (7.9%)
Postpartum infection	4 (3.2%)	0 (0%)
Preeclampsia/gestational hypertension/eclampsia	0 (0%)	5 (2.8%)
Perinatal complications		
Admittance to neonatal intensive care units (NICU)	5 (4.0%)	9 (5.1%)
Apgar score <7 at 5 min	2/125 (1.6%)	0/175 (0.0%)
Macrosomia (≥ 4500 g)	5 (4.0%)	16 (9.0%)
Girls	57 (45.6%)	77 (43.3%)
Birth weight (g)		
Mean (SD)	3829 (403)	3882 (450)
Median (interquartile range)	3815 (3547; 4102)	3917 (3563; 4199)
