# Supplementary material for: Women’s childbirth experiences in the Swedish Post-term Induction Study (SWEPIS): a multicentre, randomised, controlled trial
Source: BMJ Open. 2021 Apr 6;11(4):e042340. doi: 10.1136/bmjopen-2020-042340 (PMC8031013; doi:10.1136/bmjopen-2020-042340)

Supplementary material, Figure A

Flowchart of participants in the two randomised groups rating their overall childbirth experience on a VAS (1-10) within 3 days of delivery

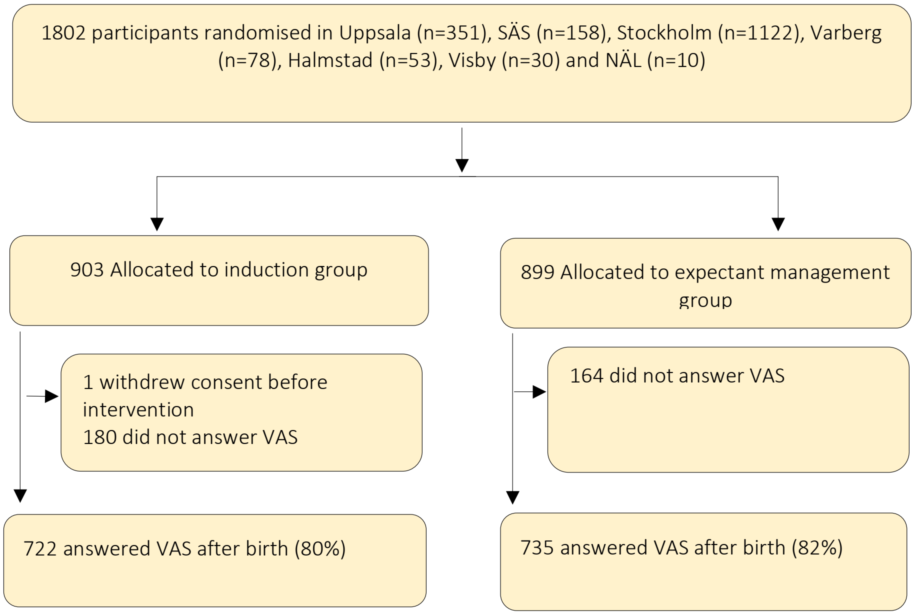

Supplement: Supplementary data [file bmjopen-2020-042340supp002.pdf]
