# Supplementary material for: Women’s childbirth experiences in the Swedish Post-term Induction Study (SWEPIS): a multicentre, randomised, controlled trial
Source: BMJ Open. 2021 Apr 6;11(4):e042340. doi: 10.1136/bmjopen-2020-042340 (PMC8031013; doi:10.1136/bmjopen-2020-042340)
Supplement: Supplementary data [file bmjopen-2020-042340supp003.pdf]

Supplementary material, Table B

Baseline characteristics for participants responding to a VAS (1-10) on overall childbirth experience, not responding to VAS, and total VAS population of women participating in SWEPIS.

	Participants responding to VAS		Participants not responding to VAS		Total VAS population	
Variables	Induction group n=722	Expectant management group n=735	Induction group n=180	Expectant management group n=164	Induction group n=902	Expectant management group n=899
Age at randomisation (years)						
Mean (SD)	31.3 (4.7)	31.3 (4.7)	31.5 (4.4)	31.7 (4.3)	31.3 (4.6)	31.0 (4.6)
Median (interquartile range)	31.2 (28; 35)	31.2 (28; 35)	31.5 (28; 34)	31.5 (29; 35)	31.2 (28; 34.6)	30.9 (28; 34.2)
< 35 years	567 (78.5%)	567 (78.5%)	141 (78.3%)	127 (77.4%)	708 (78.5%)	716 (79.6%)
≥ 35 years	155 (21.5%)	155 (21.5%)	39 (21.7%)	37 (22.6%)	194 (21.5%)	183 (20.4%)
Parity (includes stillborn or live births)						
Primiparous	402 (55.7%)	407 (55.4%)	91 (50.6%)	94 (57.3%)	493 (54.7%)	501 (55.7%)
Multiparous	320 (44.3%)	328 (44.6%)	89 (49.4%)	70 (42.7%)	402 (45.3%)	398 (44.3%)
Smoking at first antenatal visit						
No	678/695 (97.6%)	686/707 (97.0%)	172/175 (98.3%)	154/160 (96.3%)	850/870 (97.7%)	840/867 (96.9%)
Yes	17/695 (2.0%)	21/707 (2.0%)	3/175 (1.7%)	6/160 (3.8%)	20/870 (2.3%)	27/867 (3.1%)
BMI at first antenatal visit						
Mean (SD)	24.7 (4.4)	25.1 (4.9)	25.2 (5.1)	24.6 (4.2)	24.8 (4.6)	25.0 (4.8)
Median (interquartile range)	23.8 (21.7; 26.7) n=695	24 (21.6; 27.5) n=692	23.7 (22; 28) n=170	23.7 (22; 27) n=156	23.8 (21.7; 26.8) n=865	23.9 (21.6; 27.2) n=848
Region of birth						
Sweden	562/683 (82.3%)	581/697 (83.4%)	130/161 (80.7%)	120/152 (78.9%)	692/844 (82.0%)	701/849 (82.6%)
Other Nordic countries	39/683 (5.7%)	42/697 (6.0%)	8/161 (5.0%)	12/152 (7.9%)	47/844 (5.6%)	54/849 (6.4%)
Europe outside Nordic countries	10/683 (1.5%)	9/697 (1.3%)	3/161 (1.9%)	3/152 (2.0%)	13/844 (1.5%)	12/849 (1.4%)
Outside Europe	72/683 (10.5%)	65/697 (9.3%)	20/161 (12.4%)	17/152 (11.2%)	92/844 (10.9%)	82/849 (9.7%)

Nilvér H, *et al. BMJ Open* 2022; 11:e042340. doi: 10.1136/bmjopen-2020-042340

**Highest education**						
Primary school ≤ 9 years	31/657 (4.7%)	37/675 (5.4%)	9/152 (5.9%)	9/148 (6.1%)	40/809 (4.9%)	46/823 (5.8%)
High school 9 to 12 years	207/657 (31.5%)	237/675 (35.1%)	51/152 (33.6%)	45/148 (30.4%)	258/809 (31.9%)	282/823 (34.3%)
University or corresponding	419/657 (63.8%)	401/675 (59.4%)	92/152 (60.5%)	94/148 (63.5%)	511/809 (63.2%)	495/823 (60.1%)
**Gestational age at delivery** (days)						
Mean (SD)	288.9 (1.3)	291.9 (2.6)	289.2 (1.4)	291.8 (2.7)	289.0 (1.4)	291.9 (2.7)
Median (interquartile range)	289 (288; 289)	292 (290; 294)	289 (288; 290)	292 (290; 295)	289 (288; 289)	292 (290; 294)
**Time from admittance to labour ward to delivery** (hours)						
Mean (SD)	19.7 (14.6)	13.9 (12.9)	21.2 (17.4)	14.1 (12.3)	20.0 (15.2)	13.9 (12.8)
Median (interquartile range)	15.9 (8.8; 27.5) n=721	10.4 (4.6; 18.6) n=734	17.1 (9; 29)	10.1 (5; 20)	15.9 (8.8; 27.9) n=901	10.4 (4.6; 18.9) n=898
**Onset of birth process**						
Spontaneous	115 (15.9%)	489 (66.5%)	31 (17.2%)	104(63.4%)	146 (16.2%)	592 (66.0%)
Scheduled caesarean delivery	1 (0.1%)	0 (0.0%)	0 (0.0%)	1 (0.6%)	1 (0.1%)	1/899 (0.1%)
Induction	606 (83.9%)	246 (33.5%)	149 (82.8%)	59 (36.0%)	755 (83.7%)	305 (33.9%)
**Mode of birth**						
Spontaneous vaginal	601 (83.2%)	606 (82.4%)	149 (82.8%)	129 (78.7%)	750 (83.1%)	735 (81.8%)
Instrumental vaginal	46 (6.4%)	51 (6.9%)	11 (6.1%)	14 (8.5%)	95 (10.5%)	99 (11.0%)
Caesarean delivery	75 (10.4%)	78 (10.6%)	20 (11.1%)	21 (12.8%)	57 (6.3%)	65 (7.2%)
**Use of epidural anaesthesia**	346 (47.9%)	345 (46.9%)	97 (53.9%)	86 (52.4%)	443 (49.1%)	431 (47.9%)
**Maternal complications**						
Perineal lacerations III-IV	23 (3.2%)	34 (4.6%)	6 (3.3%)	8 (4.9%)	29/902 (3.2%)	42/899 (4.7%)
Postpartum haemorrhage (>1000 ml)	68 (9.4%)	88 (12%)	18 (10.0%)	13 (7.9%)	86/902 (9.5%)	101/899 (11.2%)
Postpartum infection	22 (3.0%)	16 (2.2%)	5 (2.8%)	5 (3.0%)	27/902 (3.0%)	21/899 (2.3%)
Preeclampsia/gestational hypertension/eclampsia	14 (1.9%)	26 (3.5%)	3 (1.7%)	2 (1.2%)	17/902 (1.9%)	28/899 (3.1%)

**Perinatal complications**						
Admittance to neonatal intensive care units (NICU)	28 (3.9%)	34 (4.6%)	13 (7.2%)	19 (11.7%)	41/902 (4.5%)	53/898 (5.9%)
Macrosomia (≥ 4500 g)	37 (5.1%)	58 (7.9%)	7 (3.9%)	10 (6.1%)	44/902 (4.9%)	68/898 (7.6%)
**Girls**	305 (42.2%)	340 (46.3%)	73/180 (40.6%)	69/163 (42.3%)	378/902 (41.9%)	409/898 (45.5%)
**Birth weight (g)**						
Mean (SD)	3796 (406)	3859 (424)	3891 (373)	3888 (418)	3815 (401)	3865 (423)
Median (interquartile range)	3783 (3500; 4060)	3845 (3565; 4120)	3893 (3621; 4175) n=180	3900 (3610; 4190) n=163	3804 (3530; 4090) n=902	3850 (3570; 4128) n=898
