# Supplementary material for: Women’s childbirth experiences in the Swedish Post-term Induction Study (SWEPIS): a multicentre, randomised, controlled trial
Source: BMJ Open. 2021 Apr 6;11(4):e042340. doi: 10.1136/bmjopen-2020-042340 (PMC8031013; doi:10.1136/bmjopen-2020-042340)
Supplement: Author's manuscript [file bmjopen-2020-042340.draft_revisions.pdf]

BMJ Open

BMJ Open is committed to open peer review. As part of this commitment we make the peer review history of every article we publish publicly available.

When an article is published we post the peer reviewers' comments and the authors' responses online. We also post the versions of the paper that were used during peer review. These are the versions that the peer review comments apply to.

The versions of the paper that follow are the versions that were submitted during the peer review process. They are not the versions of record or the final published versions. They should not be cited or distributed as the published version of this manuscript.

BMJ Open is an open access journal and the full, final, typeset and author-corrected version of record of the manuscript is available on our site with no access controls, subscription charges or pay-per-view fees (http://bmjopen.bmj.com).

If you have any questions on BMJ Open's open peer review process please email info.bmjopen@bmj.com

BMJ Open

# Women's childbirth experience in the Swedish Post-term Induction Study (SWEPIS): a multicentre, randomised, controlled trial

| | |
|---|---|
| Journal: | *BMJ Open* |
| Manuscript ID | bmjopen-2020-042340 |
| Article Type: | Original research |
| Date Submitted by the Author: | 02-Jul-2020 |
| Complete List of Authors: | Nilvér, Helena; Goteborgs universitet Sahlgrenska Akademin, Institution of Helath and Care Science<br>Wessberg, Anna; Goteborgs universitet Sahlgrenska Akademin, Institute of Helath and Care Sciense; Sahlgrenska universitetssjukhuset, Department of Obstetrics<br>Dencker, Anna; Goteborgs universitet Sahlgrenska Akademin, Institute of Health and Care Science<br>Hagberg, Henrik; Goteborgs universitet Sahlgrenska Akademin, Institute of Clinical Sciences, Centre of Perinatal Medicine & Health ; Sahlgrenska University Hospital, Department of Obstertics<br>Wennerholm, Ulla-Britt; Goteborgs universitet Sahlgrenska Akademin, Institute of Clinical Sciences, Centre of Perinatal Medicine &Health; Sahlgrenska University Hospital, Department of obstertics<br>Fadl, Helena; Örebro Universitet Institutionen för Medicinska Vetenskaper,<br>Sengpiel, Verena; Goteborgs universitet Sahlgrenska Akademin, Institute of Clinical Science, Centre of Perinatal Medicine Health; Sahlgrenska University Hospital, Department of Obstetrics<br>Lundgren, Ingela; Goteborgs universitet Sahlgrenska Akademin, Institute of Health and Care Science; Sahlgrenska University Hospital, Department of Obstetrics<br>Bergh, Christina; Sahlgrenska University Hospital, Department of Obstetrics<br>Wikström, Anna-Karin; Uppsala University, Department of Women's and Children's Health<br>Saltvedt, Sissel; Karolinska University Hospital, Division of Obstetrics and Gynaecology; Karolinska Institutet, Department of Women's and Children's Health<br>Wesström, Jan; Dalarna County Council, Centre for Clinical Research, Department of Women's health<br>Elden, Helen; Goteborgs universitet Sahlgrenska Akademin, Institution of Health and Care Sciences; Sahlgrenska University Hospital, Department of Obstertics |
| Keywords: | OBSTETRICS, Maternal medicine < OBSTETRICS, REPRODUCTIVE MEDICINE |
| | |

*I, the Submitting Author has the right to grant and does grant on behalf of all authors of the Work (as defined in the below author licence), an exclusive licence and/or a non-exclusive licence for contributions from authors who are: i) UK Crown employees; ii) where BMJ has agreed a CC-BY licence shall apply, and/or iii) in accordance with the terms applicable for US Federal Government officers or employees acting as part of their official duties; on a worldwide, perpetual, irrevocable, royalty-free basis to BMJ Publishing Group Ltd ("BMJ") its licensees and where the relevant Journal is co-owned by BMJ to the co-owners of the Journal, to publish the Work in this journal and any other BMJ products and to exploit all rights, as set out in our licence.*

*The Submitting Author accepts and understands that any supply made under these terms is made by BMJ to the Submitting Author unless you are acting as an employee on behalf of your employer or a postgraduate student of an affiliated institution which is paying any applicable article publishing charge ("APC") for Open Access articles. Where the Submitting Author wishes to make the Work available on an Open Access basis (and intends to pay the relevant APC), the terms of reuse of such Open Access shall be governed by a Creative Commons licence – details of these licences and which Creative Commons licence will apply to this Work are set out in our licence referred to above.*

*Other than as permitted in any relevant BMJ Author's Self Archiving Policies, I confirm this Work has not been accepted for publication elsewhere, is not being considered for publication elsewhere and does not duplicate material already published. I confirm all authors consent to publication of this Work and authorise the granting of this licence.*

For peer review only

# Women's childbirth experience in the Swedish Post-term Induction Study (SWEPIS): a multicentre, randomised, controlled trial

Helena Nilvér,[1] Anna Wessberg,[1,3] Anna Dencker,[1] Henrik Hagberg,[2,3] Ulla-Britt Wennerholm,[2,3] Helena Fadl,[4] Jan Wesström,[5] Verena Sengpiel,[2,3] Ingela Lundgren,[1,3] Christina Bergh,[3] Anna-Karin Wikström,[6] Sissel Saltvedt,[7] Helen Elden[1,3]

[1]Institute of Health and Care Sciences, Sahlgrenska Academy, University of Gothenburg, Gothenburg, Sweden

[2]Centre of Perinatal Medicine & Health, Institute of Clinical Sciences, Sahlgrenska Academy, Gothenburg, Sweden

[3]Department of Obstetrics, Sahlgrenska University Hospital, Region Västra Götaland, Gothenburg, Sweden

[4]Department of Obstetrics and Gynaecology, Faculty of Medicine and Health, Örebro University, Örebro, Sweden

[5]Department of Women's and Children's Health, Uppsala University, Uppsala, Sweden; Centre for Clinical Research Dalarna, Falun, Sweden

[6]Department of Women's and Children's Health, Uppsala University, Uppsala, Sweden

[7]Department of Women's and Children's Health, Karolinska Institute, Karolinska University Hospital, Stockholm, Sweden

Corresponding author:
Helena Nilvér
Institute of Health and Care Sciences
Sahlgrenska Academy
University of Gothenburg
Box 457
Gothenburg SE-405 30
Sweden
Helena.nilver@gu.se

For peer review only

# ABSTRACT

## Objective

To compare women's childbirth experience with induction of labour at 41 weeks versus expectant management and induction of labour at 42 weeks in the Swedish Post-term Induction Study (SWEPIS).

## Design

A register based, multicentre, randomised, controlled superiority trial.

## Setting

Women were recruited at 14 hospitals in Sweden, May 2016 - October 2018.

## Participants

Women with an uncomplicated singleton pregnancy were recruited at 41 gestational weeks.

## Interventions

The women were randomly assigned to induction of labour at 41 weeks (induction group, n=1381), or expectant management until 42 weeks (expectant management group, n=1379).

## Outcome measures

The women's childbirth experiences were measured using the Childbirth Experience Questionnaire version 2 (CEQ2), in 656 women, three months after the birth at three hospitals. The overall childbirth experience in 1457 women using a Visual Analogue Scale (VAS 1-10) within three days after delivery at the remaining eleven hospitals.

## Results

The total response rate was 77% (2113/2760). There were no significant differences in childbirth experience measured with CEQ2 between the groups in the subscales; *Own capacity* (2.8 versus 2.7, p=0.09), *Perceived safety* (3.3 versus 3.2, p=0.06) and *Professional support* (3.6 versus 3.5, p=0.38) or in the total CEQ2 score (3.3 versus 3.2, p=0.07) respectively. Women in the induction group scored significantly higher in the subscale *Participation* (3.6 versus 3.4, p=0.02) than women in the expectant management group, with a small effect size (0.19). No significant difference was observed in overall childbirth experience according to VAS (8.0 versus 8.1, p=0.22).

## Conclusions

There were no differences in childbirth experience according to CEQ2 or overall childbirth experience assessed with VAS between women randomly assigned to induction of labour at 41 weeks or expectant management and induction of labour at 42 weeks. The women's overall ratings of their childbirth experiences were high.


## Trial registration

Current Controlled Trials, ISRCTN26113652.



For peer review only - http://bmjopen.bmj.com/site/about/guidelines.xhtml

For peer review only

## KEYWORDS

Childbirth experience, Childbirth Experience Questionnaire, induction of labour, prolonged pregnancy, randomised controlled trial

For peer review only



For peer review only - http://bmjopen.bmj.com/site/about/guidelines.xhtml

## STRENGTH AND LIMITATIONS OF THIS STUDY

- The childbirth experiences were measured in the SWEdish Post term Induction study (SWEPIS), a large national randomised controlled multicentre trial including 2760 women at 14 hospitals in Sweden.
- The validated Childbirth Experience Questionnaire, version 2 (CEQ2) was used at 3/14 hospitals and data on a Visual Analogue Scale (VAS) with reference to overall childbirth experience were retrieved from the Swedish Pregnancy Register for the remaining 11/14 hospitals.
- The total response rate was high (77%).
- A limitation is that we did not measure CEQ2 at all hospitals, and the response rate with CEQ2 was higher in the induction group than in the expectant management group.
- The results of this study may be generalised to women with an uncomplicated pregnancy who are motivated to induce labour at 41 weeks.

For peer review only

## INTRODUCTION

Women's childbirth experiences are individual, complex life events, which are related to the outcome for the mother and child.[1-3] These experiences may leave long lasting impressions, as women tend to remember their childbirth experiences very well.[4-6] A positive birth experience can strengthen the woman and be an empowering life event[5 7] while a negative experience can increase the risk of postpartum depression,[8] fear of childbirth[9 10] and future fear of vaginal births.[11] Women with traumatic childbirth experiences have identified the lack and loss of control, and insufficient practical and emotional support, as the main sources of their traumatic experiences.[12] A qualitative study on women's positive childbirth experiences showed that being in a safe and supporting environment gave them a sense of control, and made it possible to focus on strategies to manage childbirth. Knowledge about the birth process was also important, helping the women to perceive control and enable them to take part in decision-making.[13]

Earlier studies have reported induction of labour being associated with a more negative childbirth experience than spontaneous onset of labour.[3 14 15] However, in prolonged pregnancies, this difference was not as obvious.[3 16] Induction of labour in prolonged pregnancies can relieve some women from feelings of discomfort, and the uncertainty of not knowing when and where labour will begin.[17] Two qualitative interview studies have described that women in a prolonged pregnancy may have growing worries about their unborn child's health, and doubts about their body's ability to initiate labour.[18 19] Women also described a lack of information and not being seen by health professionals.[19]

A systematic review of qualitative studies describes that induction of labour can require a shift in expectations as the woman needs to reconsider her original birth plan to a more medicalized one.[16] To our knowledge, there is only one previous randomised controlled trial (RCT)[20] comparing induction of labour at 41 weeks with expectant management until 42 weeks and 5 days (n=508). The majority of women in both groups stated that they would prefer to be induced at 41 weeks instead of undergoing expectant management in a future prolonged pregnancy.[20] Another RCT compared women's childbirth experiences of induction of labour at 39 weeks with expectant management until spontaneous onset or medical indication for induction of labour, in nulliparous women aged 35



For peer review only - http://bmjopen.bmj.com/site/about/guidelines.xhtml

or older, using the Childbirth Experience Questionnaire (CEQ) (n=618). No significant differences were found between the groups.[21]

Induction at or beyond term, compared with expectant management is related to a lower incidence of perinatal death.[22] Still, induction of labour at 42 weeks has been the current standard management in Sweden. The present study is part of the SWEdish Post Term Induction Study (SWEPIS), a national, multicentre, randomised, controlled, superiority trial.[23] The aim was to evaluate if induction of labour at 41 weeks (n=1381), compared with expectant management and induction of labour at 42 weeks (n=1379), improved perinatal and maternal outcomes. The study was stopped in advance due to ethical reasons related to six cases of perinatal deaths in the expectant management group and none in the induction group. The rates of perinatal mortality and morbidity (primary composite outcome), caesarean deliveries and instrumental vaginal deliveries were similar in both groups.

A woman's childbirth experience is essential for her psychological wellbeing and health. Therefore, the aim of this study was to compare childbirth experiences in women randomly assigned to induction of labour at 41 weeks with women randomly assigned to expectant management and induction of labour at 42 weeks within the SWEPIS.

# METHODS

## Study design

The study reports on childbirth experience within SWEPIS,[23 24] and was performed from May 2016 to October 2018 at 14 Swedish hospitals. The trial was a register based, multicentre, randomised, controlled, superiority trial. The protocol is available online (www-ncbi-nlm-nih-gov.proxy.kib.ki.se/pubmed/26951777) and as a publication.[24] It was undertaken with support from the Swedish Network for National Clinical Studies within Obstetrics and Gynaecology (SNAKS, www.snaks.se).

## Participants

Eligible participants in SWEPIS were women ≥18 years with an uncomplicated singleton pregnancy, with cephalic presentation at 40 weeks+6 days (40+6) to 41 weeks+1 day (41+1). Women were excluded if they had had a previous caesarean delivery or other uterine surgery, had pregestational or insulin-dependent gestational diabetes, hypertensive disorder of pregnancy, a multiple pregnancy, breech or transverse position of the foetus, diagnosed oligohydramnios, the foetus being small for gestational age or foetal malformation, or contraindications for vaginal delivery.

## Study logistics

The women were given a written leaflet with information about SWEPIS at their regular antenatal check–up at 40 weeks. If interested, they were invited to phone or e-mail the study coordinators, who then gave additional information about the study and booked an appointment at 40+6 to 41+1 weeks. At the visit, the study was further explained, eligibility was confirmed, and written consent to participate was obtained. Randomisation was done online with a module set up by the Swedish Pregnancy Register.[25] Allocation to the trial group, 1:1 was done using central online randomisation by dynamic allocation, to minimise imbalance between the groups. Study centre and parity (primiparity versus multiparity) were used as minimisation variables. All participants gave written informed consent. Women randomised to the induction group were appointed for induction at 41+0



For peer review only - http://bmjopen.bmj.com/site/about/guidelines.xhtml

to 41+2 weeks. Women randomised to the expectant management group were appointed for induction at 42+0 to 42+1 if they had not given birth at that time.

## Data collection

Women included at Sahlgrenska University Hospital, Örebro University Hospital and Falun Hospital were asked to complete *The Childbirth Experience Questionnaire Version 2* (CEQ2) three months after birth.[26 27] These three hospitals were chosen with the aim of including different sizes of hospitals in Sweden, representing urban and rural populations. The CEQ2 was sent through a link via e-mail with two reminders.

Women who participated at the remaining centres (Uppsala University Hospital, Södra Älvsborg Hospital, Region Stockholm [n=5 hospitals], Varberg Hospital, Halmstad Hospital, Visby Hospital and Norra Älvsborg County Hospital) were asked, according to clinical routine in Sweden, by healthcare staff to rate their overall childbirth experience on a VAS (1-10) within three days of delivery. Data on VAS assessing women's childbirth experiences were retrieved from the Swedish Pregnancy Registry.[25]

Data on background variables, pregnancy and delivery outcomes were retrieved from the Swedish Pregnancy Register.[25]

## Outcome measures

*The Childbirth Experience Questionnaire 2* (CEQ2) was used as the main outcome measure, and was a pre-specified secondary outcome in SWEPIS. The CEQ2 questionnaire was available in Swedish and English. A *Visual Analogue Scale* (VAS) on the woman's overall childbirth experiences was added as an exploratory outcome in the hospitals where CEQ2 was not distributed.

## The Childbirth Experience Questionnaire (CEQ)

The first CEQ[28] was developed and validated in Sweden, then translated and culturally adapted to several other languages.[29-32] In the present study, a revised version, CEQ2,[26 27] was used. CEQ2 consists of 22 questions considering four different subscales of the childbirth experience; *Own capacity* (8 items), *Perceived safety* (6 items), *Professional support* (5 items) and *Participation* (3 items), see Table 1. Responses to the items are given on a 4-point Likert scale, ranging from 1 =totally disagree, 2=mostly disagree, 3=mostly agree and 4=totally agree. Three of the items (perceived pain, control and sense of security) are rated on a visual analogue scale (VAS 1-100), which are categorized as 0-40=1, 41-60=2, 61-80=3 and 81-100=4. Negatively worded items are reversed in scoring. Higher scores represent a more positive childbirth experience. The score of each subscale is calculated as a mean of the individual domains. The total CEQ2 score is the mean of the four individual subscale scores (1-4).

*Table 1* Subscales and items in the Childbirth Experience Questionnaire version 2 (CEQ2)

*Own Capacity*

Labour and birth went as I had expected.
I felt strong during labour and birth.
I felt capable during labour and birth.



For peer review only - http://bmjopen.bmj.com/site/about/guidelines.xhtml

I was tired during labour and birth.
I felt happy during labour and birth.
I felt that I handled the situation well.
As a whole, how painful did you feel childbirth was?
As a whole, how much control did you feel you had during childbirth?

*Perceived safety*
I felt scared during labour and birth.
My impression of the team's medical skills made me feel secure.
I have many positive memories from childbirth.
I have many negative memories from childbirth.
Some of my memories from childbirth make me feel depressed.
As a whole, how secure did you feel during childbirth?

*Professional support*
Both my partner and I were treated with warmth and respect.
I would have preferred the midwife to be more present during labour and birth.
I would have preferred more encouragement from the midwife.
The midwife conveyed an atmosphere of calm.
The midwife helped me to find my inner strength.

*Participation*
I wish the staff had listened to me more during labour and birth.
I took part in decisions regarding my care and treatment as much as I wanted.
I received the information I needed during labour and birth.

## Overall childbirth experience measured with VAS

As part of the clinical routine in Sweden, a question on overall childbirth experience, with a VAS, was used to assess the women's overall childbirth experience within three days after delivery. It was added as an exploratory outcome and retrieved for women in the hospitals where CEQ was not distributed. Women were asked by a midwife to rate their childbirth experience on a VAS ranging from 1, representing a very negative experience, to 10, representing a very positive experience.[33] When needed, this could be undertaken with the assistance of an interpreter. A rating of 8-10 is considered a very good childbirth experience.[33]

## Patient and Public involvement

Participants and public were not involved in the design of the study or in the conduction of the trial. The study findings will be disseminated to the participants and public through popular science articles.

## Data analysis

Mean, standard deviation, Median, 25-75 quartiles and 95% confidence intervals were calculated. The Mann-Whitney U-test was used to compare the differences in childbirth experience (CEQ2 and VAS) between groups, and Fisher's exact test for categorical variables. Cohen's effect size was used to estimate clinical differences between groups, where an effect size of 0.2 was considered small, 0.5 medium and 0.8 as large.[34] All tests were two-sided, using a significance level of 5%. Results were analysed using SPSS, version 25.0 (SPSS Inc., Chicago, IL, USA) or SAS version 9 (SAS Institute, Cary, NC, USA).



For peer review only - http://bmjopen.bmj.com/site/about/guidelines.xhtml

# RESULTS

In all, 2760 participants were included in SWEPIS. Baseline characteristics were similar in the two treatment groups.[23] In total, 77% (2113/2760) responded to either CEQ2, 68% (656/959), or VAS, 81% (1457/1801).

## CEQ

In total, 960 women who were randomised at three centres: Gothenburg (May 20, 2016 to October 15, 2018), Falun (January 23, 2017 to October 15, 2018) and Örebro (September 9, 2017 to October 15, 2018) were asked to fill in the web questionnaire. One woman in the induction group withdraw her consent to participate before induction of labour, and 22 participants were not fluent in Swedish or English, and thus did not receive the questionnaire. Due to practical and technical issues, another 26 participants did not received the questionnaire, most often caused by the woman's e-mail address not being registered at randomisation due to technical issues in the registration module. A total of 78% (354/455) of women in the induction group and 66% (302/456) of women in the expectant management group responded to CEQ2 three months after the birth (Figure 1 - Flowchart).

Of the women in Falun, Gothenburg and Örebro (n=656) that responded to the CEQ2, 88.6% (542/612) were born in Sweden and 71.6% (411/574) had a university education. Of these women, 35.4% (232/656) had spontaneous onset of labour and 67.1% (420/656) had induction of labour. In the women who did not respond to the CEQ2, 73.4% (207/282) were born in Sweden, 59.1% (152/257) had a university education, 47.5% (144/303) had spontaneous onset of labour and 52.1% (158/303) had induction of labour (Table 2 and Supplementary material, Table A).

Table 2. Baseline characteristics for participants answering the CEQ2

| Variables | Induction group n=354 | Expectant management group n=302 |
|---|---|---|
| Age at randomisation (years) | 31.3 (5.0)<br>31 (28; 35) | 31.2 (4.2)<br>31 (28; 34) |
| < 35 years | 271 (76.6%) | 245 (81.1%) |
| ≥ 35 years | 83 (23.4%) | 57 (18.9%) |
| **Parity (includes stillborn or live births)** | | |
| Primiparous | 196 (55.4%) | 164 (54.3%) |
| Multiparous | 158 (44.6%) | 138 (45.7%) |
| **Smoking at first antenatal visit** | | |
| No | 296/302 (98.0%) | 254/259 (97%) |
| Yes | 6/302 (2.0%) | 5/259 (1.7%) |
| **BMI at first antenatal visit** | 25.2 (5.0)<br>24.1 (21.5; 27.7)<br>n=302 | 25.2 (4.8)<br>24.2 (21.8; 27.6)<br>n=267 |
| **Region of birth** | | |



For peer review only - http://bmjopen.bmj.com/site/about/guidelines.xhtml

| | | |
|---|---|---|
| Sweden | 293/330 (88.8%) | 249/282 (88.3%) |
| Other Nordic countries | 15/330 (4.5%) | 18/282 (6.4%) |
| Europe outside Nordic countries | 6/330 (1.8%) | 4/282 (1.4%) |
| Outside Europe | 16/330 (4.8%) | 14/282 (5.0%) |
| **Highest education** | | |
| Primary school ≤ 9 years | 9/310 (2.9%) | 5/264 (1.9%) |
| High school 9 to 12 years | 83/310 (26.8%) | 66/264 (25.0%) |
| University or corresponding | 218/310 (70.3%) | 193/264 (73.1%) |
| **Gestational age at delivery** (days) | 288.0 (1.0)<br>288.5 (288;289) | 291.7 (2.7)<br>292 (289; 294) |
| **Time from admittance to labour ward to delivery** (hours) | 20.1 (14.2)<br>15.9 (9.5; 27.8) | 13.3 (11.0)<br>10.7 (4.5; 19.3) |
| **Onset of birth process** | | |
| Spontaneous | 31 (8.8%) | 201 (66.6%) |
| Scheduled caesarean delivery | 4 (1.1%) | 0 (0.0%) |
| Induction | 319 (90.1%) | 101 (33.4%) |
| **Mode of birth** | | |
| Spontaneous vaginal | 296 (83.6%) | 256 (84.8%) |
| Instrumental vaginal | 24 (6.8%) | 14 (4.6%) |
| Caesarean delivery | 34 (9.6%) | 32 (10.6%) |
| **Use of epidural anaesthesia** | 199 (56.2 %) | 154 (51%) |
| **Maternal complications** | | |
| Perineal lacerations III-IV | 7 (2.0%) | 4 (1.3%) |
| Postpartum haemorrhage (>1000 ml) | 38 (10.7%) | 31 (10.3%) |
| Postpartum infection | 11 (3.1%) | 6 (2.0%) |
| Preeclampsia/gestational hypertension/eclampsia | 2 (0.6%) | 9 (3.0%) |
| **Perinatal complications** | | |
| Admittance to neonatal intensive care units (NICU) | 9 (2.5%) | 20 (6.6%) |
| Apgar score <7 at 5 min | 3/354 (0.8%) | 5/301 (1.7%) |
| Macrosomia (≥ 4500 g) | 19 (5.4%) | 30 (9.9%) |
| **Girls** | 165 (46.6%) | 136 (45.0%) |
| **Birth weight (g)** | 3811 (430)<br>3800 (3530; 4066) | 3905 (465)<br>3898 (3588; 4201) |

For categorical variables n/N (%) is presented.

For continuous variables Mean (standard deviation) / Median (interquartile range Q1; Q3) / n= is presented.



For peer review only - http://bmjopen.bmj.com/site/about/guidelines.xhtml

For participants answering the CEQ2, the baseline characteristics are presented in Table 2. In total, 90.1% (319/354) of the women in the induction group had their labour induced, compared with 33.4% (101/302) of the women in the expectant management group. The gestational age at delivery was on average 3 days longer in the expectant management group. The number of newborns admitted to neonatal intensive care units was 2.5% (9/354) in the induction group and 6.6% (20/302) in the expectant management group. The rates of spontaneous vaginal births were similar (83.6% versus 84.8% respectively).

The results of the CEQ2 scores are presented for each domain, as well as the total CEQ score, in Table 3. There were no incomplete questionnaires. No significant differences in childbirth experience were identified in three of the four subscales (*Own capacity*, *Perceived safety* and *Professional support)* or in the total score in the CEQ2 between the two randomised groups. There was a statistically significant difference in the subscale *Participation,* where women in the induction group scored slightly higher than women in the expectant management group (p=0.02), with a small effect size (0.19).

**Table 3. Childbirth experience in the induction group and the expectant management group, according to the Childbirth Experience Questionnaire 2 (CEQ2)**

| | Induction group n=354 | Expectant management group n=302 | Effect size | p-value |
|---|---|---|---|---|
| **Own Capacity** | 2.8 (0.6)<br>2.9 (2.3-3.2)<br>(2.7-2.85) | 2.7 (0.6)<br>2.8 (2.3-3.2)<br>(2.6-2.8) | 0.16 | 0.09 |
| **Perceived Safety** | 3.3 (0.7)<br>3.5 (3.0-3.8)<br>(3.3-3.4) | 3.2 (0.8)<br>3.5 (2.7-3.8)<br>(3.1-3.3) | 0.17 | 0.06 |
| **Professional Support** | 3.6 (0.5)<br>3.8 (3.4-4.0)<br>(3.5-3.6) | 3.5 (0.6)<br>3.8 (3.4-4.0)<br>(3.4-3.6) | 0.09 | 0.38 |
| **Participation** | 3.6 (0.6)<br>4.0 (3.3-4.0)<br>(3.5-3.6) | 3.4 (0.7)<br>3.7 (3.0-4.0)<br>(3.4-3.5) | 0.19 | 0.02 |
| **Total CEQ** | 3.3 (0.5)<br>3.5 (3.0-3.7)<br>(3.3-3.4) | 3.2 (0.6)<br>3.4 (2.9-3.7)<br>(3.2-3.3) | 0.17 | 0.07 |

Mean (standard deviation) / Median (interquartile range Q1; Q3) / (95% Confidence interval for Mean)

p-values calculated with Mann-Whitney U-test.



For peer review only - http://bmjopen.bmj.com/site/about/guidelines.xhtml

## VAS

In total, 1802 women were randomised at the remaining participating centres (Uppsala University Hospital, Södra Älvsborg Hospital, Region Stockholm, Varberg Hospital, Halmstad Hospital, Visby Hospital and Norra Älvsborg County Hospital). One woman in the induction group withdrew her consent to participate before induction of labour. A total of 80% (722/902) of women in the induction group and 82% (735/899) women in the expectant management group rated their overall childbirth experience on a VAS (1-10) within 3 days of delivery (Supplementary material, Figure A). The women responding to VAS had similar background characteristics to the women that did not answer VAS. Of the women responding to VAS, 82.8% (1143/1380) were born in Sweden, 61.6% (820/1332) had a university education, and 58.7% (855/1457) had their labour induced. In women not responding to VAS, 79.9% (250/313) were born in Sweden, 62.0% (186/300) had a university education and 60.5% (208/344) had their labour induced (Supplementary material, Table B and Table C).

In the induction group, 83.9% (606/722) of women had their labour induced, compared with 33.5% (246/735) of women in the expectant management group. Women in the expectant management group had a pregnancy on average 3 days longer than women in the induction group. There were similar modes of birth in the two groups, 83.2% (601/722) women had a spontaneous vaginal birth in the induction group, and 82.4% (606/735) women in the expectant management group (Supplementary Material, Table C).

There was no significant difference between the induction group and the expectant management group in overall childbirth experience on VAS, or in the number of women rating their childbirth experience as positive (VAS 8-10) (Table 4).

**Table 4. Childbirth experience measured with VAS 1-10**

| | Induction group n=722 | Expectant management group n=735 | Effect size | p-value |
|---|---|---|---|---|
| **VAS** | 8.0 (1.2)<br>8 (7-10)<br>(7.9-8.2) | 8.1 (2.0)<br>9 (7-10)<br>(8.0-8.3) | -0.05 | 0.22 |
| **VAS 8-10** | 497 (69%) | 528 (72%) | | 0.23 |

Mean (standard deviation) / Median (interquartile range Q1; Q3) / (95% CI for Mean)

For continuous variables, p-values calculated with the Mann-Whitney U-test.

For categorical variables n/N (%) is presented and p-value calculated with the $X^2$ Pearson Chi-Square.

## DISCUSSION

The main finding in this study was that there were no significant differences in women's childbirth experiences between women randomised to induction of labour at 41 weeks and women randomised to expectant management and induction of labour at 42 weeks, in terms of the total CEQ2 score, three of four subscales (*Own Capacity, Perceived safety, Professional support)* or



For peer review only - http://bmjopen.bmj.com/site/about/guidelines.xhtml

according to VAS. However, the women in the induction group scored slightly higher in the domain *Participation* compared with the women in the expectant management group. Women reported an overall positive childbirth experience.

Induction of labour has been reported to have a negative impact on women's childbirth experience.[8 9 12 15] However, this was not observed in our study, even though women had their labour induced to a larger extent in the induction group than in the expectant management group. The women had uncomplicated pregnancies and might have been more motivated for induction of labour as they were willing to participate. Furthermore, induction can be perceived as a relief from feelings of discomfort in a prolonged pregnancy[17] and a way to meet the child sooner.[35] Another explanation for the results may be that there were no differences in caesarean deliveries or instrumental vaginal births between the two randomised groups,[23] factors that can have a negative impact on women's childbirth experience.[4 28 36]

None of the subscales *Own Capacity*, *Perceived Safety*, *Professional support* showed significant differences. Most women have expectations of their capacity to give birth and hope they will be able to use their own inherited physical and psychosocial capacities.[37] It is also reported that most women want a vaginal birth, focusing both on safety and psychosocial wellbeing.[37] A safe and supporting environment enables the woman to focus on techniques to manage the birth.[13]

Women in the induction group scored significantly higher in the subscale *Participation* than the expectant management group (3.6 versus 3.4, maximum score 4, p=0.02). In a systematic review of qualitative studies, Downe, et al.[37] found that most women want a physiological labour and birth. However, if interventions were needed, women wanted sufficient information and to be active in the decision-making to retain a sense of personal achievement and control.[37] This may be reflected in the domain *Participation* as the women who participated in the study may have been more motivated for induction of labour, and therefore those allocated to the induction group may have experienced being part of the decision-making to a higher degree than those randomised to the expectant management group (standard care).

The mean total CEQ2 score in the women in the induction group was 3.3, and 3.2 in the expectant management group. This is in agreement with earlier studies using the CEQ2. In the RCT by Walker et al women at age 35 or older were randomised to either induction of labour at 39 weeks or expectant management until spontaneous onset or medical indication to induce labour (n=618).[21] The women in the induction group scored a total mean CEQ2 score of 3.03 versus 2.96 (p=0.12) in the expectant management group.[21] Also, women with vaginal delivery scored a mean total CEQ2 score of 3.3 in the CEQ2 validation study.[26]

In a retrospective cohort study from Sweden, including all women with a singleton pregnancy, using the VAS for assessment of overall birth experience, 69% (11 493/16 775) of women scored VAS 8-10 (mean 7.94).[15] These scores are similar to the scores in our study, where 69% in the induction group and 72% in the expectant management group scored 8-10 on VAS.

## Methodological considerations

By using CEQ2[26 28] to compare women's childbirth experiences, we chose an instrument with good psychometric properties.[38] Women could answer the CEQ2 in Swedish or English, which enabled more participants to fill in the questionnaire. The CEQ has been used in several studies in Sweden, and has been shown to have good internal and external validity.[39-42] The advantages of CEQ and CEQ2 are that they measure the multidimensional experience of labour and birth. However, there might be dimensions of the childbirth experience that are difficult to capture with a questionnaire.



For peer review only - http://bmjopen.bmj.com/site/about/guidelines.xhtml

A larger proportion of women answered the CEQ2 in the induction group (78%) than in the expectant management group (66%). This may be due to women not perceiving they were participating in a study when they were allocated to the expectant management group, which was standard care at the time. Furthermore, women with spontaneous onset of labour did not answer the questionnaire to the same extent as women whose labour was induced. However, these differences in response rate between the randomisation groups were not seen for VAS. This might be explained by the fact that the VAS is used as part of the clinical routine including all women in the participating hospitals.

We added the VAS as an exploratory variable, and it was  retrieved from the Swedish Pregnancy Register[25] with the purpose of obtaining information on overall childbirth experience in the women at the 11 hospitals were the CEQ2 was not distributed. The advantage of the VAS is that it is part of the clinical routine and therefore there might be less bias in the women's responses. On the other hand, it is a simplified and non-specific measure, and the women responded to it within three days after the birth, compared to the CEQ which was distributed 3 months after delivery and reflects four domains of childbirth. Hence, the childbirth experience may shift with time when the first wave of relief after the birth has settled. However, the VAS has been shown to correlate to women's overall childbirth experiences and is considered to be useful as a tool to screen for childbirth experience.[15 41 43]

A limitation of the study is that only 22% of eligible women participated in SWEPIS.[23] The baseline characteristics were similar for several background variables between the study population in SWEPIS and a Swedish background population. However, more women had completed a university education (64% vs. 55%) and were born in Sweden (83% vs. 73.%) in SWEPIS compared to the background population.[23] Oral and written information was given by the midwives at a regular antenatal check-up at week 40, but it is unclear how many women actually received the information. The caregiver's attitudes towards the study, as well as the influence of friends and family, might have affected the women's decision to participate.[44 45] It is possible that women participating in SWEPIS were more motivated for or positive to induction of labour than women who declined or chose not to contact the study team. If a specific intervention has negative associations, this may be a reason for not participating, and if it is considered favourable, a reason for participating.[46] Another reason for declining could have been that some women wanted a natural birth without interventions. The results of this study may be generalised to women with an uncomplicated pregnancy who are motivated to induce labour at 41 weeks.

## CONCLUSION

The main result of this study was that there were no significant differences in women's childbirth experiences between women randomised to induction of labour at 41 weeks and women randomised to expectant management and induction of labour at 42 weeks. However, women randomised to induction scored higher on the CEQ2 subscale *Participation* but the effect size was small. Overall, women's ratings of their childbirth experience were high.

Further research is needed to describe women's experiences of induction of labour in a prolonged pregnancy, and how the health care system can meet these women's expectations and needs, in order to support them during pregnancy and childbirth.



For peer review only - http://bmjopen.bmj.com/site/about/guidelines.xhtml

## Acknowledgments

The authors wish to thank all the women who participated in the study and took their time to answer the questionnaire, and all the midwives and obstetricians at the participating centres. We thank and acknowledge the Swedish Network for National Clinical studies (SNAKS) for their assistance and support.

## Contributions

AW, AD, HH, UBW, VS and HE conceived and designed the study. HN, AW, HH, UBW, HF, JW, AKW and SS oversaw recruitment of study participants and collection of CEQ data at the local centres. UBW and HN did the data cleaning together with statisticians Mattias Molin and Per Ekman. HN compiled and analysed the CEQ2 data. Per Ekman did the analysis regarding VAS. HN wrote the first draft of the manuscript, which was then critically reviewed and revised by all co-authors. All authors have approved the final version of the manuscript for submission. All authors have full access to all the data in the study and take responsibility for the integrity of the data and the accuracy of the data analysis. HE is the guarantor.

## Competing interests

None of the authors have any conflict of interest concerning this paper.

## Funding

This study was supported by the Swedish state under the agreement between the Swedish government and the county councils, the ALF-agreement (ALFGBG-440301, ALFGBG-718721,ALFGBG-70940, ALFGBG-426401), the Health Technology Centre at Sahlgrenska University Hospital, the Foundation of the Health and Medical care committee of the Region of Västra Götaland, Sweden (VGFOUREG387351, VGFOUREG640891, VGFOUREG854081), Hjalmar Svensson Foundation, the foundation Mary von Sydow, born Wijk donation fund, Uppsala-Örebro regional research council (RFR-556711, RFR-736891), Region Örebro County research committee (OLL-715501), the ALF-agreement in Stockholm (ALF-561222,ALF-562222, ALF-563222), and Centre for Clinical Research Dalarna-Uppsala University, Sweden (CKFUU-417011). The funders had no role in the study design, data collection, data analysis, data interpretation, or writing of the report. The researchers were independent of the funders.

## Ethics approval

This study was approved by the Regional Ethics Board in Gothenburg, Sweden, in May 2014 (Dnr: 285–14) and later complementary applications (T 905-15, T 291-16, T 1180-16, T 330-17, T 1066-17, T 087-18, T 347-18, T 961-18, T 1110-18).

## Consent to participate

Informed written consent was obtained from all women prior to randomisation.

## Data availability statement

Data is available from the corresponding author on reasonable request.

For peer review only

# REFERENCES


1. Larkin P, Begley CM, Devane D. Women's experiences of labour and birth: an evolutionary concept analysis. *Midwifery* 2009;25(2):e49-59. doi: 10.1016/j.midw.2007.07.010 [published Online First: 2007/11/13]
2. Lundgren I. Swedish women's experience of childbirth 2 years after birth. *Midwifery* 2005;21(4):346-54. doi: 10.1016/j.midw.2005.01.001 [published Online First: 2005/07/19]
3. Hildingsson I, Karlstrom A, Nystedt A. Women's experiences of induction of labour--findings from a Swedish regional study. *The Australian & New Zealand journal of obstetrics & gynaecology* 2011;51(2):151-7. doi: 10.1111/j.1479-828X.2010.01262.x [published Online First: 2011/04/07]
4. Bossano CM, Townsend KM, Walton AC, et al. The maternal childbirth experience more than a decade after delivery. *American journal of obstetrics and gynecology* 2017;217(3):342.e1-42.e8. doi: 10.1016/j.ajog.2017.04.027 [published Online First: 2017/04/30]
5. Simkin P. Just another day in a woman's life? Women's long-term perceptions of their first birth experience. Part I. *Birth (Berkeley, Calif)* 1991;18(4):203-10. [published Online First: 1991/12/01]
6. Simkin P. Just another day in a woman's life? Part II: Nature and consistency of women's long-term memories of their first birth experiences. *Birth (Berkeley, Calif)* 1992;19(2):64-81. [published Online First: 1992/06/01]
7. Olza I, Leahy-Warren P, Benyamini Y, et al. Women's psychological experiences of physiological childbirth: a meta-synthesis. *BMJ open* 2018;8(10):e020347. doi: 10.1136/bmjopen-2017-020347 [published Online First: 2018/10/21]
8. Bell AF, Andersson E. The birth experience and women's postnatal depression: A systematic review. *Midwifery* 2016;39:112-23. doi: 10.1016/j.midw.2016.04.014 [published Online First: 2016/06/21]
9. Nilsson C, Lundgren I. Women's lived experience of fear of childbirth. *Midwifery* 2009;25(2):e1-9. doi: 10.1016/j.midw.2007.01.017 [published Online First: 2007/05/22]
10. Dencker A, Nilsson C, Begley C, et al. Causes and outcomes in studies of fear of childbirth: A systematic review. *Women and birth : journal of the Australian College of Midwives* 2019;32(2):99-111. doi: 10.1016/j.wombi.2018.07.004 [published Online First: 2018/08/18]
11. Pang MW, Leung TN, Lau TK, et al. Impact of first childbirth on changes in women's preference for mode of delivery: follow-up of a longitudinal observational study. *Birth (Berkeley, Calif)* 2008;35(2):121-8. doi: 10.1111/j.1523-536X.2008.00225.x [published Online First: 2008/05/30]
12. Hollander MH, van Hastenberg E, van Dillen J, et al. Preventing traumatic childbirth experiences: 2192 women's perceptions and views. *Archives of women's mental health* 2017;20(4):515-23. doi: 10.1007/s00737-017-0729-6 [published Online First: 2017/05/30]
13. Karlstrom A, Nystedt A, Hildingsson I. The meaning of a very positive birth experience: focus groups discussions with women. *BMC pregnancy and childbirth* 2015;15:251. doi: 10.1186/s12884-015-0683-0 [published Online First: 2015/10/11]
14. Henderson J, Redshaw M. Women's experience of induction of labor: a mixed methods study. *Acta obstetricia et gynecologica Scandinavica* 2013;92(10):1159-67. doi: 10.1111/aogs.12211 [published Online First: 2013/07/03]
15. Falk M, Nelson M, Blomberg M. The impact of obstetric interventions and complications on women's satisfaction with childbirth a population based cohort study including 16,000 women. *BMC pregnancy and childbirth* 2019;19(1):494. doi: 10.1186/s12884-019-2633-8 [published Online First: 2019/12/13]
16. Lou S, Hvidman L, Uldbjerg N, et al. Women's experiences of postterm induction of labor: A systematic review of qualitative studies. *Birth (Berkeley, Calif)* 2018 doi: 10.1111/birt.12412 [published Online First: 2018/12/19]




For peer review only - http://bmjopen.bmj.com/site/about/guidelines.xhtml

17. Moore JE, Low LK, Titler MG, et al. Moving toward patient-centered care: Women's decisions, perceptions, and experiences of the induction of labor process. *Birth (Berkeley, Calif)* 2014;41(2):138-46. doi: 10.1111/birt.12080 [published Online First: 2014/04/08]
18. Wessberg A, Lundgren I, Elden H. Late-term pregnancy: Navigating in unknown waters - A hermeneutic study. *Women and birth : journal of the Australian College of Midwives* 2019 doi: 10.1016/j.wombi.2019.03.011 [published Online First: 2019/04/06]
19. Wessberg A, Lundgren I, Elden H. Being in limbo: Women's lived experiences of pregnancy at 41 weeks of gestation and beyond - A phenomenological study. *BMC pregnancy and childbirth* 2017;17(1):162. doi: 10.1186/s12884-017-1342-4 [published Online First: 2017/06/06]
20. Heimstad R, Romundstad PR, Hyett J, et al. Women's experiences and attitudes towards expectant management and induction of labor for post-term pregnancy. *Acta obstetricia et gynecologica Scandinavica* 2007;86(8):950-6. doi: 10.1080/00016340701416929 [published Online First: 2007/07/27]
21. Walker KF, Bugg GJ, Macpherson M, et al. Randomized Trial of Labor Induction in Women 35 Years of Age or Older. *N Engl J Med* 2016;374(9):813-22. doi: 10.1056/NEJMoa1509117 [published Online First: 2016/03/11]
22. Middleton P, Shepherd E, Crowther CA. Induction of labour for improving birth outcomes for women at or beyond term. *The Cochrane database of systematic reviews* 2018;5:Cd004945. doi: 10.1002/14651858.CD004945.pub4 [published Online First: 2018/05/10]
23. Wennerholm U-B, Saltvedt S, Wessberg A, et al. Induction of labour at 41 weeks versus expectant management and induction of labour at 42 weeks (SWEdish Post-term Induction Study, SWEPIS): multicentre, open label, randomised, superiority trial. *BMJ (Clinical research ed)* 2019;367:l6131. doi: 10.1136/bmj.l6131
24. Elden H, Hagberg H, Wessberg A, et al. Study protocol of SWEPIS a Swedish multicentre register based randomised controlled trial to compare induction of labour at 41 completed gestational weeks versus expectant management and induction at 42 completed gestational weeks. *BMC pregnancy and childbirth* 2016;16:49. doi: 10.1186/s12884-016-0836-9 [published Online First: 2016/03/10]
25. Stephansson O, Petersson K, Bjork C, et al. The Swedish Pregnancy Register - for quality of care improvement and research. *Acta obstetricia et gynecologica Scandinavica* 2018;97(4):466-76. doi: 10.1111/aogs.13266 [published Online First: 2017/11/25]
26. Dencker A, Bergqvist L, Berg M, et al. Measuring women's experiences of decision-making and aspects of midwifery support: a confirmatory factor analysis of the revised Childbirth Experience Questionnaire. *BMC pregnancy and childbirth* 2020;20(1):199. doi: 10.1186/s12884-020-02869-0 [published Online First: 2020/04/08]
27. Walker KF, Dencker A, Thornton JG. Childbirth experience questionnaire 2: Validating its use in the United Kingdom. *European journal of obstetrics & gynecology and reproductive biology: X* 2020;5:100097. doi: 10.1016/j.eurox.2019.100097 [published Online First: 2020/02/06]
28. Dencker A, Taft C, Bergqvist L, et al. Childbirth experience questionnaire (CEQ): development and evaluation of a multidimensional instrument. *BMC pregnancy and childbirth* 2010;10:81. doi: 10.1186/1471-2393-10-81 [published Online First: 2010/12/15]
29. Abbaspoor Z, Moghaddam-Banaem L, Ronaghi S, et al. Translation and Cultural Adaptation of the Childbirth Experience Questionnaire (CEQ) in Iran. *Iranian journal of nursing and midwifery research* 2019;24(4):296-300. doi: 10.4103/ijnmr.IJNMR_103_18 [published Online First: 2019/07/25]
30. Soriano-Vidal FJ, Oliver-Roig A, Cabrero-Garcia J, et al. The Spanish version of the Childbirth Experience Questionnaire (CEQ-E): reliability and validity assessment. *BMC pregnancy and childbirth* 2016;16(1):372. doi: 10.1186/s12884-016-1100-z [published Online First: 2016/11/26]



For peer review only - http://bmjopen.bmj.com/site/about/guidelines.xhtml

31. Walker KF, Wilson P, Bugg GJ, et al. Childbirth experience questionnaire: validating its use in the United Kingdom. *BMC pregnancy and childbirth* 2015;15:86. doi: 10.1186/s12884-015-0513-4 [published Online First: 2015/04/18]
32. Zhu X, Wang Y, Zhou H, et al. Adaptation of the Childbirth Experience Questionnaire (CEQ) in China: A multisite cross-sectional study. *PloS one* 2019;14(4):e0215373. doi: 10.1371/journal.pone.0215373 [published Online First: 2019/04/25]
33. Graviditetsregistrets Årsrapport 2018 (The Swedish Pregnancy Register yearly report 2018) [Available from: https://www.medscinet.com/GR/uploads/hemsida/dokumentarkiv/2018%20GR%20%C3%A5rsrapport_2.0.pdf.
34. Cohen J. A power primer. *Psychological bulletin* 1992;112(1):155.
35. Gatward H, Simpson M, Woodhart L, et al. Women's experiences of being induced for post-date pregnancy. *Women and birth : journal of the Australian College of Midwives* 2010;23(1):3-9. doi: 10.1016/j.wombi.2009.06.002 [published Online First: 2009/08/04]
36. Nystedt A, Hildingsson I. Women's and men's negative experience of child birth-A cross-sectional survey. *Women and birth : journal of the Australian College of Midwives* 2018;31(2):103-09. doi: 10.1016/j.wombi.2017.07.002 [published Online First: 2017/08/10]
37. Downe S, Finlayson K, Oladapo OT, et al. What matters to women during childbirth: A systematic qualitative review. *PloS one* 2018;13(4):e0194906. doi: 10.1371/journal.pone.0194906 [published Online First: 2018/04/18]
38. Nilver H, Begley C, Berg M. Measuring women's childbirth experiences: a systematic review for identification and analysis of validated instruments. *BMC pregnancy and childbirth* 2017;17(1):203. doi: 10.1186/s12884-017-1356-y [published Online First: 2017/07/01]
39. Bergqvist L, Dencker A, Taft C, et al. Women's experiences after early versus postponed oxytocin treatment of slow progress in first childbirth--a randomized controlled trial. *Sexual & reproductive healthcare : official journal of the Swedish Association of Midwives* 2012;3(2):61-5. doi: 10.1016/j.srhc.2012.03.003 [published Online First: 2012/05/15]
40. Hildingsson I, Rubertsson C. Childbirth experiences among women with fear of birth randomized to internet-based cognitive therapy or midwife counseling. *Journal of psychosomatic obstetrics and gynaecology* 2019:1-10. doi: 10.1080/0167482x.2019.1634047 [published Online First: 2019/06/28]
41. Turkmen S, Tjernstrom M, Dahmoun M, et al. Post-partum duration of satisfaction with childbirth. *The journal of obstetrics and gynaecology research* 2018;44(12):2166-73. doi: 10.1111/jog.13775 [published Online First: 2018/07/31]
42. Ulfsdottir H, Saltvedt S, Georgsson S. Women's experiences of waterbirth compared with conventional uncomplicated births. *Midwifery* 2019;79:102547. doi: 10.1016/j.midw.2019.102547 [published Online First: 2019/10/15]
43. Larsson C, Saltvedt S, Edman G, et al. Factors independently related to a negative birth experience in first-time mothers. *Sexual & reproductive healthcare : official journal of the Swedish Association of Midwives* 2011;2(2):83-9. doi: 10.1016/j.srhc.2010.11.003 [published Online First: 2011/03/29]
44. Monteiro TM, Katz L, Bento SF, et al. Reasons given by pregnant women for participating in a clinical trial aimed at preventing premature delivery: a qualitative analysis. *BMC pregnancy and childbirth* 2019;19(1):97. doi: 10.1186/s12884-019-2240-8 [published Online First: 2019/03/22]
45. van der Zande ISE, van der Graaf R, Hooft L, et al. Facilitators and barriers to pregnant women's participation in research: A systematic review. *Women and birth : journal of the Australian College of Midwives* 2018;31(5):350-61. doi: 10.1016/j.wombi.2017.12.009 [published Online First: 2018/01/27]




For peer review only - http://bmjopen.bmj.com/site/about/guidelines.xhtml

46. Oude Rengerink K, Logtenberg S, Hooft L, et al. Pregnant womens' concerns when invited to a randomized trial: a qualitative case control study. *BMC pregnancy and childbirth* 2015;15:207. doi: 10.1186/s12884-015-0641-x [published Online First: 2015/09/06]

For peer review only

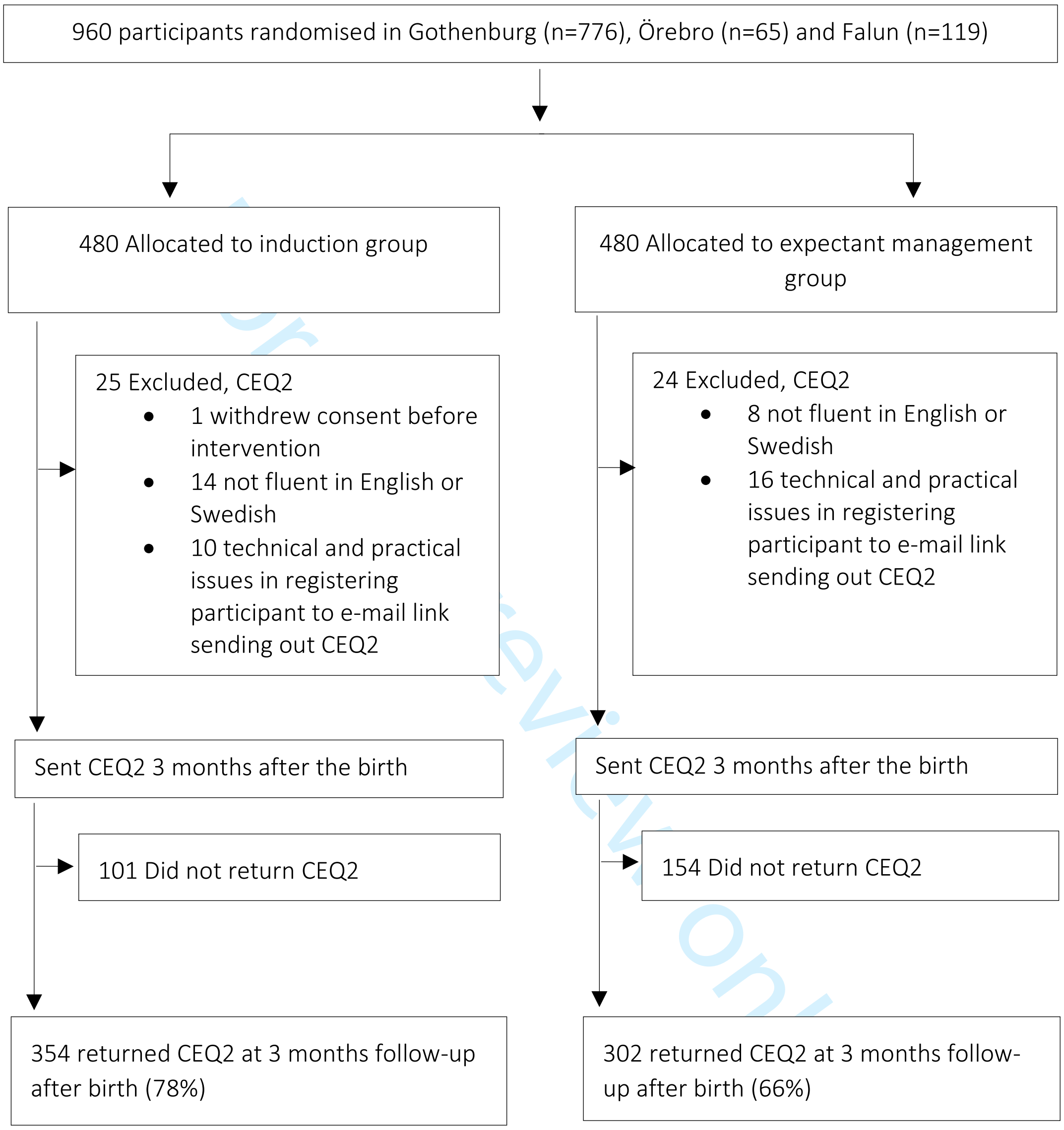


**Figure 1.** Flowchart of the women in the two randomised groups that responded to CEQ2 three months after birth

Supplementary material, Table A
Baseline characteristics for participants not answering CEQ2

| Variables | Induction group n=125 | Expectant management group n=178 |
|---|---|---|
| Age at randomisation (years) | 30.7 (4.8)<br>30 (27; 34) | 30.8 (4.8)<br>30 (27; 35) |
| < 35 years | 99 (79.2%) | 139 (78.1%) |
| ≥ 35 years | 26 (20.8%) | 39 (21.9%) |
| Parity (includes stillborn or live births) | | |
| Primiparous | 73 (58.4%) | 88 (49.4%) |
| Multiparous | 52 (41.6%) | 90 (50.6%) |
| Smoking at first antenatal visit | | |
| No | 96/102 (94.1%) | 134/141 (95.0%) |
| Yes | 6/102 (5.9%) | 7/141 (5.0%) |
| BMI at first antenatal visit | 25.4 (5.0)<br>23.9 (21.8; 28.3)<br>n=108 | 25.7 (5.4)<br>24.4 (22.0; 28.0)<br>n=150 |
| Region of birth | | |
| Sweden | 84/115 (64.2%) | 123/167 (73.7%) |
| Other Nordic countries | 12/115 (10.4&) | 12/167 (7.2%) |
| Europe outside Nordic countries | 1/115 (1.0%) | 2/167 (1.2%) |
| Outside Europe | 18/115 (15.7%) | 12/167 (7.2%) |
| Highest education | | |
| Primary school ≤ 9 years | 5/102 (4.9%) | 6/155 (3.9%) |
| High school 9 to 12 years | 37/102 (36.2%) | 57/155 (36.8%) |
| University or corresponding | 60/102 (58.8%) | 92/155 (59.4%) |
| Gestational age at delivery (days) | 288.7 (1.6)<br>288 (288; 289) | 291.1 (2.6)<br>291 (289; 294) |
| Time from admittance to labour ward to delivery (hours) | 21.1 (13.9)<br>18.1 (10.5; 27.8) | 12.8 (10.8)<br>10.1 (5.1; 18.0) |
| Onset of birth process | | |
| Spontaneous | 18 (14.4%) | 126 (70.(%) |
| Scheduled caesarean delivery | 0 (0%) | 1 (0.6%) |
| Induction | 107 (85.6%) | 51 (28.7%) |
| Mode of birth | | |
| Spontaneous vaginal | 104 (83.2%) | 149 (83.7%) |
| Instrumental vaginal | 7 (5.6%) | 12 (6.7%) |
| Caesarean delivery | 14 (11.2%) | 17 (9.6%) |
| Use of epidural anaesthesia | 87 (69.6%) | 84 (47.2%) |
| Maternal complications | | |
| Perineal lacerations III-IV | 4 (3.2%) | 4 (2.2%) |
| Postpartum haemorrhage (>1000 ml) | 16 (12.8%) | 14 (7.9%) |
| Postpartum infection | 4 (3.2%) | 0 (0%) |
| Preeclampsia/gestational hypertension/eclampsia | 0 (0%) | 5 (2.8%) |
| Perinatal complications | | |
| Admittance to neonatal intensive care units (NICU) | 5 (4.0%) | 9 (5.1%) |
| Apgar score <7 at 5 min | 2/125 (1.6%) | 0/175 (0.0%) |
| Macrosomia (≥ 4500 g) | 5 (4.0%) | 16 (9.0%) |
| Girls | 57 (45.6%) | 77 (43.3%) |
| Birth weight (g) | 3829 (403)<br>3815 (3547; 4102) | 3882 (450)<br>3917 (3563; 4199) |

For categorical variables n/N (%) is presented.
For continuous variables Mean (standard deviation) / Median (interquartile range Q1; Q3) / n= is presented.

For peer review only

**Supplementary material, Figure A**

Flowchart of participants in the two randomised groups rating their overall childbirth experience on a VAS (1-10) within 3 days of delivery

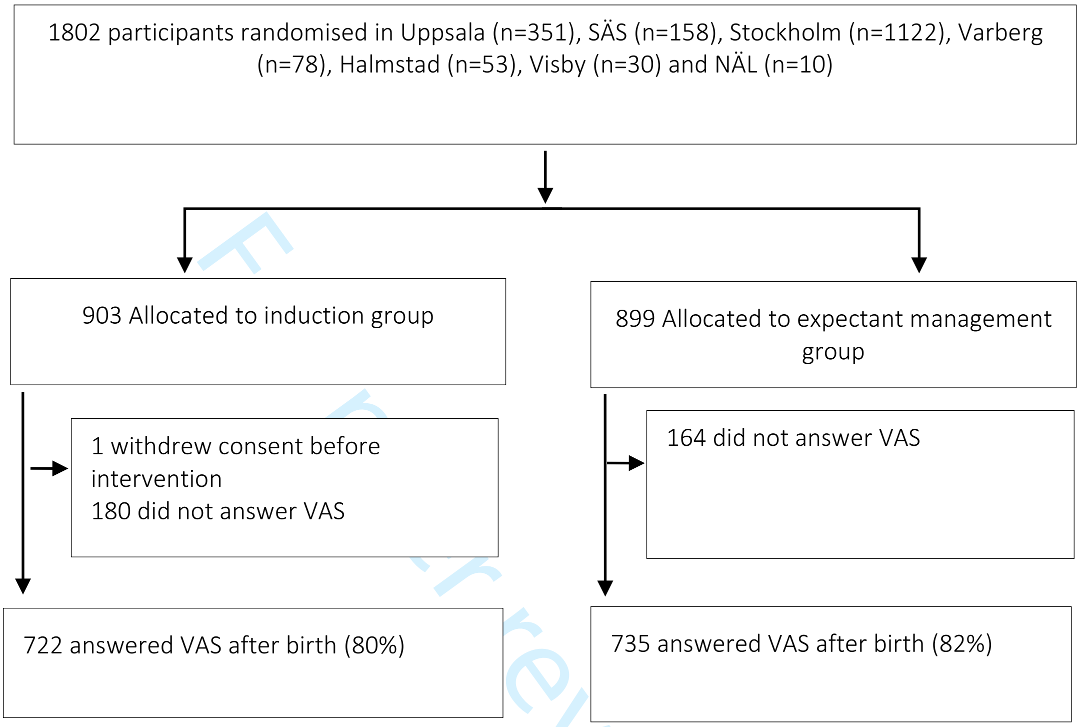


For peer review only

Supplementary material, Table B

Baseline characteristics for participants not answering VAS

| Variables | Induction group n=180 | Expectant management group n=164 |
|---|---|---|
| **Age at randomisation** (years) | 31.5 (4.4)<br>31.5 (28; 34) | 31.7 (4.3)<br>31.5 (29; 35) |
| < 35 years | 141 (78.3%) | 127 (77.4%) |
| ≥ 35 years | 39 (21.7%) | 37 (22.6%) |
| **Parity (includes stillborn or live births)** | | |
| Primiparous | 91/180 (50.6%) | 94/164 (57.3%) |
| Multiparous | 89/180 (49.4%) | 70/164 (42.7%) |
| **Smoking at first antenatal visit** | | |
| No | 172/175 (98.3%) | 154/160 (96.3%) |
| Yes | 3/175 (1.7%) | 6/160 (3.8%) |
| **BMI at first antenatal visit** | 25.2 (5.1)<br>23.7 (22; 28)<br>n=170 | 24.6 (4.2)<br>23.7 (22; 27)<br>n=156 |
| **Region of birth** | | |
| Sweden | 130/161 (80.7%) | 120/152 (78.9%) |
| Other Nordic countries | 8/161 (5.0%) | 12/152 (7.9%) |
| Europe outside Nordic countries | 3/161 (1.9%) | 3/152 (2.0%) |
| Outside Europe | 20/161 (12.4%) | 17/152 (11.2%) |
| **Highest education** | | |
| Primary school ≤ 9 years | 9/152 (5.9%) | 9/148 (6.1%) |
| High school 9 to 12 years | 51/152 (33.6%) | 45/148 (30.4%) |
| University or corresponding | 92/152 (60.5%) | 94/148 (63.5%) |
| **Gestational age at delivery** (days) | 289.2 (1.4)<br>289 (288; 290) | 291.8 (2.7)<br>292 (290; 295) |
| **Time from admittance to labour ward to delivery** (hours) | 21.2 (17.4)<br>17.1 (9; 29) | 14.1 (12.3)<br>10.1 (5; 20) |
| **Onset of birth process** | | |
| Spontaneous | 31 (17.2%) | 104(63.4%) |
| Scheduled caesarean delivery | 0 (0.0%) | 1 (0.6%) |
| Induction | 149 (82.8%) | 59 (36.0%) |
| **Mode of birth** | | |
| Spontaneous vaginal | 149 (82.8%) | 129 (78.7%) |
| Instrumental vaginal | 11 (6.1%) | 14 (8.5%) |
| Caesarean delivery | 20 (11.1%) | 21 (12.8%) |
| **Use of epidural anaesthesia** | 97 (53.9%) | 86 (52.4%) |
| **Perinatal complications** | | |
| Admittance to neonatal intensive care units (NICU) | 13 (7.2%) | 19 (11.7%) |
| Macrosomia (≥ 4500 g) | 7 (3.9%) | 10 (6.1%) |
| **Girls** | 73/180 (40.6%) | 69/163 (42.3%) |
| **Birth weight (g)** | 3891 (373)<br>3893 (3621; 4175)<br>n=180 | 3888 (418)<br>3900 (3610; 4190)<br>n=163 |

For categorical variables n/N (%) is presented.

For continuous variables Mean (standard deviation) / Median (interquartile range Q1; Q3) / n= is presented.

review only

Supplementary material, Table C
Baseline characteristics for participants answering VAS

| Variables | Induction group n=722 | Expectant management group n=735 |
|---|---|---|
| Age at randomisation (years) | 31.3 (4.7)<br>31.2 (28; 35) | 30.9 (4.7)<br>30.7 (28; 34) |
| < 35 years | 567/722 (78.5%) | 589/735 (80.1%) |
| ≥ 35 years | 155/722 (21.5%) | 146/735 (19.9%) |
| Parity (includes stillborn and live births) | | |
| Primiparous | 402/722 (55.7%) | 407/735 (55.4%) |
| Multiparous | 320/722 (44.3%) | 328/735 (44.6%) |
| Smoking at first antenatal visit | | |
| No | 678/695 (97.6%) | 686/707 (97.0%) |
| Yes | 17/695 (2.0%) | 21/707 (2.0%) |
| BMI at first antenatal visit | 24.7 (4.4)<br>23.8 (21.7; 26.7)<br>n=695 | 25.1 (4.9)<br>24 (21.6; 27.5)<br>n=692 |
| Region of birth | | |
| Sweden | 562/683 (82.3%) | 581/697 (83.4%) |
| Other Nordic countries | 39/683 (5.7%) | 42/697 (6.0%) |
| Europe outside Nordic countries | 10/683 (1.5%) | 9/697 (1.3%) |
| Outside Europe | 72/683 (10.5%) | 65/697 (9.3%) |
| Highest education | | |
| Primary school ≤ 9 years | 31/657 (4.7%) | 37/675 (5.4%) |
| High school 9 to 12 years | 207/657 (31.5%) | 237/675 (35.1%) |
| University or corresponding | 419/657 (63.8%) | 401/675 (59.4%) |
| Gestational age at delivery (days) | 288.9 (1.3)<br>289 (288; 289) | 291.9 (2.6)<br>292 (290; 294) |
| Time from admittance to labour ward to delivery (hours) | 19.7 (14.6)<br>15.9 (8.8; 27.5)<br>n=721 | 13.9 (12.9)<br>10.4 (4.6; 18.6)<br>n=734 |
| Onset of birth process | | |
| Spontaneous | 115 (15.9%) | 489 (66.5%) |
| Scheduled caesarean delivery | 1 (0.1%) | 0 (0.0%) |
| Induction | 606 (83.9%) | 246 (33.5%) |
| Mode of birth | | |
| Spontaneous vaginal | 601 (83.2%) | 606 (82.4%) |
| Instrumental vaginal | 46 (6.4%) | 51 (6.9%) |
| Caesarean delivery | 75 (10.4%) | 78 (10.6%) |
| Use of epidural anaesthesia | 346 (47.9%) | 345 (46.9%) |
| Maternal complications | | |
| Perineal lacerations III-IV | 23 (3.2%) | 34 (4.6%) |
| Postpartum haemorrhage (>1000 ml) | 68 (9.4%) | 88 (12%) |
| Postpartum infection | 22 (3.0%) | 16 (2.2%) |
| Preeclampsia/gestational hypertension/eclampsia | 14 (1.9%) | 26 (3.5%) |
| Perinatal complications | | |
| Admittance to neonatal intensive care units (NICU) | 28 (3.9%) | 34 (4.6%) |
| Macrosomia (≥ 4500 g) | 37 (5.1%) | 58 (7.9%) |
| Girls | 305 (42.2%) | 340 (46.3%) |
| Birth weight (g) | 3796 (406)<br>3783 (3500; 4060) | 3859 (424)<br>3845 (3565; 4120) |

For categorical variables n/N (%) is presented.
For continuous variables Mean (standard deviation) / Median (interquartile range Q1; Q3) / n= is presented.

For peer review only

# CONSORT 2010 checklist of information to include when reporting a randomised trial*

| Section/Topic | Item No | Checklist item | Reported on page No |
|---|---|---|---|
| **Title and abstract** | | | |
| | 1a | Identification as a randomised trial in the title | yes |
| | 1b | Structured summary of trial design, methods, results, and conclusions (for specific guidance see CONSORT for abstracts) | 2 |
| **Introduction** | | | |
| Background and objectives | 2a | Scientific background and explanation of rationale | 3 |
| | 2b | Specific objectives or hypotheses | 4 |
| **Methods** | | | |
| Trial design | 3a | Description of trial design (such as parallel, factorial) including allocation ratio | 4 |
| | 3b | Important changes to methods after trial commencement (such as eligibility criteria), with reasons | 3 |
| Participants | 4a | Eligibility criteria for participants | 4 |
| | 4b | Settings and locations where the data were collected | 4 |
| Interventions | 5 | The interventions for each group with sufficient details to allow replication, including how and when they were actually administered | 4 |
| Outcomes | 6a | Completely defined pre-specified primary and secondary outcome measures, including how and when they were assessed | 5 |
| | 6b | Any changes to trial outcomes after the trial commenced, with reasons | No changes |
| Sample size | 7a | How sample size was determined | This was a secondary outcome measure |
| | 7b | When applicable, explanation of any interim analyses and stopping guidelines | - |
| Randomisation: | | | |
| Sequence generation | 8a | Method used to generate the random allocation sequence | 4 |
| | 8b | Type of randomisation; details of any restriction (such as blocking and block size) | 4 |
| Allocation concealment mechanism | 9 | Mechanism used to implement the random allocation sequence (such as sequentially numbered containers), describing any steps taken to conceal the sequence until interventions were assigned | 4 |


For peer review only - http://bmjopen.bmj.com/site/about/guidelines.xhtml

| | | | |
|---|---|---|---|
| Implementation | 10 | Who generated the random allocation sequence, who enrolled participants, and who assigned participants to interventions | 4 |
| Blinding | 11a | If done, who was blinded after assignment to interventions (for example, participants, care providers, those assessing outcomes) and how | - |
| | 11b | If relevant, description of the similarity of interventions | - |
| Statistical methods | 12a | Statistical methods used to compare groups for primary and secondary outcomes | 6 |
| | 12b | Methods for additional analyses, such as subgroup analyses and adjusted analyses | 6 |
| **Results** | | | |
| Participant flow (a diagram is strongly recommended) | 13a | For each group, the numbers of participants who were randomly assigned, received intended treatment, and were analysed for the primary outcome | 6 |
| | 13b | For each group, losses and exclusions after randomisation, together with reasons | 7 |
| Recruitment | 14a | Dates defining the periods of recruitment and follow-up | 6 |
| | 14b | Why the trial ended or was stopped | 3 |
| Baseline data | 15 | A table showing baseline demographic and clinical characteristics for each group | 7 |
| Numbers analysed | 16 | For each group, number of participants (denominator) included in each analysis and whether the analysis was by original assigned groups | 7 |
| Outcomes and estimation | 17a | For each primary and secondary outcome, results for each group, and the estimated effect size and its precision (such as 95% confidence interval) | 8 |
| | 17b | For binary outcomes, presentation of both absolute and relative effect sizes is recommended | - |
| Ancillary analyses | 18 | Results of any other analyses performed, including subgroup analyses and adjusted analyses, distinguishing pre-specified from exploratory | - |
| Harms | 19 | All important harms or unintended effects in each group (for specific guidance see CONSORT for harms) | - |
| **Discussion** | | | |
| Limitations | 20 | Trial limitations, addressing sources of potential bias, imprecision, and, if relevant, multiplicity of analyses | 10-11 |
| Generalisability | 21 | Generalisability (external validity, applicability) of the trial findings | 11 |
| Interpretation | 22 | Interpretation consistent with results, balancing benefits and harms, and considering other relevant evidence | 9-10 |
| **Other information** | | | |
| Registration | 23 | Registration number and name of trial registry | 2 |
| Protocol | 24 | Where the full trial protocol can be accessed, if available | - |
| Funding | 25 | Sources of funding and other support (such as supply of drugs), role of funders | 12 |

For peer review only


For peer review only - http://bmjopen.bmj.com/site/about/guidelines.xhtml

*We strongly recommend reading this statement in conjunction with the CONSORT 2010 Explanation and Elaboration for important clarifications on all the items. If relevant, we also recommend reading CONSORT extensions for cluster randomised trials, non-inferiority and equivalence trials, non-pharmacological treatments, herbal interventions, and pragmatic trials. Additional extensions are forthcoming: for those and for up to date references relevant to this checklist, see www.consort-statement.org.

For peer review only

BMJ Open

# Women's childbirth experience in the Swedish Post-term Induction Study (SWEPIS): a multicentre, randomised, controlled trial

| | |
|---|---|
| Journal: | *BMJ Open* |
| Manuscript ID | bmjopen-2020-042340.R1 |
| Article Type: | Original research |
| Date Submitted by the Author: | 26-Dec-2020 |
| Complete List of Authors: | Nilvér, Helena; Göteborgs Universitet, Institution of Helath and Care Science<br>Wessberg, Anna; Goteborgs universitet Sahlgrenska Akademin, Institute of Helath and Care Sciense; Sahlgrenska universitetssjukhuset, Department of Obstetrics<br>Dencker, Anna; Goteborgs universitet Sahlgrenska Akademin, Institute of Health and Care Science<br>Hagberg, Henrik; Goteborgs universitet Sahlgrenska Akademin, Institute of Clinical Sciences, Centre of Perinatal Medicine & Health ; Sahlgrenska University Hospital, Department of Obstertics<br>Wennerholm, Ulla-Britt; Goteborgs universitet Sahlgrenska Akademin, Institute of Clinical Sciences, Centre of Perinatal Medicine &Health; Sahlgrenska University Hospital, Department of obstertics<br>Fadl, Helena; Örebro Universitet Institutionen för Medicinska Vetenskaper,<br>Wesström, Jan; Dalarna County Council, Centre for Clinical Research, Department of Women's health<br>Sengpiel, Verena; Goteborgs universitet Sahlgrenska Akademin, Institute of Clinical Science, Centre of Perinatal Medicine Health; Sahlgrenska University Hospital, Department of Obstetrics<br>Lundgren, Ingela; Goteborgs universitet Sahlgrenska Akademin, Institute of Health and Care Science; Sahlgrenska University Hospital, Department of Obstetrics<br>Bergh, Christina; Goteborgs Universitet, Institute of Clinical Sciences; Sahlgrenska universitetssjukhuset, Reproductive Medicine<br>Wikström, Anna-Karin; Uppsala University, Department of Women's and Children's Health<br>Saltvedt, Sissel; Karolinska University Hospital, Division of Obstetrics and Gynaecology; Karolinska Institutet, Department of Women's and Children's Health<br>Elden, Helen; Goteborgs universitet Sahlgrenska Akademin, Institution of Health and Care Sciences; Sahlgrenska University Hospital, Department of Obstertics |
| <b>Primary Subject Heading</b>: | Obstetrics and gynaecology |
| Secondary Subject Heading: | Obstetrics and gynaecology |
| Keywords: | OBSTETRICS, Maternal medicine < OBSTETRICS, REPRODUCTIVE |

| | MEDICINE |
|---|---|
| | |

SCHOLARONE™
Manuscripts

*I, the Submitting Author has the right to grant and does grant on behalf of all authors of the Work (as defined in the below author licence), an exclusive licence and/or a non-exclusive licence for contributions from authors who are: i) UK Crown employees; ii) where BMJ has agreed a CC-BY licence shall apply, and/or iii) in accordance with the terms applicable for US Federal Government officers or employees acting as part of their official duties; on a worldwide, perpetual, irrevocable, royalty-free basis to BMJ Publishing Group Ltd ("BMJ") its licensees and where the relevant Journal is co-owned by BMJ to the co-owners of the Journal, to publish the Work in this journal and any other BMJ products and to exploit all rights, as set out in our licence.*

*The Submitting Author accepts and understands that any supply made under these terms is made by BMJ to the Submitting Author unless you are acting as an employee on behalf of your employer or a postgraduate student of an affiliated institution which is paying any applicable article publishing charge ("APC") for Open Access articles. Where the Submitting Author wishes to make the Work available on an Open Access basis (and intends to pay the relevant APC), the terms of reuse of such Open Access shall be governed by a Creative Commons licence – details of these licences and which Creative Commons licence will apply to this Work are set out in our licence referred to above.*

*Other than as permitted in any relevant BMJ Author's Self Archiving Policies, I confirm this Work has not been accepted for publication elsewhere, is not being considered for publication elsewhere and does not duplicate material already published. I confirm all authors consent to publication of this Work and authorise the granting of this licence.*

For peer review only

# Women's childbirth experience in the Swedish Post-term Induction Study (SWEPIS): a multicentre, randomised, controlled trial

For peer review only

Helena Nilvér,[1] Anna Wessberg,[1, 3] Anna Dencker,[1] Henrik Hagberg,[2, 3] Ulla-Britt Wennerholm,[2, 3] Helena Fadl,[4] Jan Wesström,[5] Verena Sengpiel, [2, 3] Ingela Lundgren,[1, 3] Christina Bergh,[2, 6] Anna-Karin Wikström,[7] Sissel Saltvedt,[8] Helen Elden[1, 3]

[1]Institute of Health and Care Sciences, Sahlgrenska Academy, University of Gothenburg, Gothenburg, Sweden

[2]Centre of Perinatal Medicine & Health, Department of Obstetrics and gynaecology, Institute of Clinical Sciences, Sahlgrenska Academy, Gothenburg, Sweden

[3]Department of Obstetrics, Sahlgrenska University Hospital, Region Västra Götaland, Gothenburg, Sweden

[4]Department of Obstetrics and Gynaecology, Faculty of Medicine and Health, Örebro University, Örebro, Sweden

[5]Department of Women's and Children's Health, Uppsala University, Uppsala, Sweden; Centre for Clinical Research Dalarna, Falun, Sweden

[6]Reproductive Medicine, Sahlgrenska University hospital, Gothenburg, Sweden

[7]Department of Women's and Children's Health, Uppsala University, Uppsala, Sweden

[8]Department of Women's and Children's Health, Karolinska Institute, Karolinska University Hospital, Stockholm, Sweden

Corresponding author:
Helena Nilvér
Institute of Health and Care Sciences
Sahlgrenska Academy
University of Gothenburg
Box 457
Gothenburg SE-405 30
Sweden
Helena.nilver@gu.se



For peer review only - http://bmjopen.bmj.com/site/about/guidelines.xhtml

# ABSTRACT

## Objective

To compare childbirth experiences in women randomly assigned to either induction of labour at 41 weeks or to expectant management until 42 weeks in the Swedish Post-term Induction Study (SWEPIS).

## Design

A register based, multicentre, randomised, controlled superiority trial.

## Setting

Women were recruited at 14 hospitals in Sweden, 2016 - 2018.

## Participants

Women with an uncomplicated singleton pregnancy were recruited at 41 gestational weeks.

## Interventions

The women were randomly assigned to induction of labour at 41 weeks (induction group, n=1381), or expectant management until 42 weeks (expectant management group, n=1379).

## Outcome measures

As main outcome, women's childbirth experiences were measured using the Childbirth Experience Questionnaire version 2 (CEQ2), in 656 women, three months after the birth at three hospitals. As exploratory outcome, overall childbirth experience was measured in 1457 women using a Visual Analogue Scale (VAS 1-10) within three days after delivery at the remaining eleven hospitals.

## Results

The total response rate was 77% (2113/2760). There were no significant differences in childbirth experience measured with CEQ2 between the groups (induction group n=354, expectant management group n=302) in the subscales; *Own capacity* (2.8 versus 2.7, p=0.09), *Perceived safety* (3.3 versus 3.2, p=0.06) and *Professional support* (3.6 versus 3.5, p=0.38) or in the total CEQ2 score (3.3 versus 3.2, p=0.07) respectively. Women in the induction group scored higher in the subscale *Participation* (3.6 versus 3.4, p=0.02), although with a small effect size (0.19). No significant difference was observed in overall childbirth experience according to VAS (8.0 (n=735) versus 8.1 (n=735), p=0.22).

## Conclusions

There were no differences in childbirth experience according to CEQ2 or overall childbirth experience assessed with VAS between women randomly assigned to induction of labour at 41 weeks or expectant management until 42 weeks. Overall, women rated their childbirth experiences high.

## Trial registration

Current Controlled Trials, ISRCTN26113652.


For peer review only



For peer review only - http://bmjopen.bmj.com/site/about/guidelines.xhtml

## KEYWORDS

Childbirth experience, Childbirth Experience Questionnaire, induction of labour, prolonged pregnancy, randomised controlled trial

For peer review only



For peer review only - http://bmjopen.bmj.com/site/about/guidelines.xhtml

## STRENGTH AND LIMITATIONS OF THIS STUDY

- The study had a high total response rate.
- The study was based on a large randomised controlled trial.
- Childbirth experience was evaluated with a validated questionnaire (CEQ2) 3 months after birth.
- The questionnaire (CEQ2) was used in only three of 14 participating centres; other centres evaluated the overall childbirth experience with a visual analogue scale (VAS) as an exploratory outcome.
- The response rate with CEQ2 was higher in the induction group than in the expectant management group.

For peer review only

## INTRODUCTION

Women's childbirth experiences are individual, complex life events, which are related to the outcome for the mother and child.[1-3] These experiences may leave long lasting impressions, as women tend to remember their childbirth experiences very well.[4-6] A positive birth experience can strengthen the woman and be an empowering life event[5 7] while a negative experience can increase the risk of postpartum depression,[8] fear of childbirth[9 10] and future fear of vaginal births.[11] Women with traumatic childbirth experiences have identified the lack and loss of control, and insufficient practical and emotional support, as the main sources of their traumatic experiences.[12] A qualitative study on women's positive childbirth experiences showed that being in a safe and supporting environment gave them a sense of control, and made it possible to focus on strategies to manage childbirth. Knowledge about the birth process was also important, helping the women to perceive control and enable them to take part in decision-making.[13]

Earlier studies have reported induction of labour being associated with a more negative childbirth experience than spontaneous onset of labour.[3 14 15] However, in prolonged pregnancies, this difference was not as obvious.[3 16] Induction of labour in prolonged pregnancies can relieve some women from feelings of discomfort, and the uncertainty of not knowing when and where labour will begin.[17] Women's trust in health professionals as well as an impatience to deliver a healthy child can make women to choose and accept induction of labour.[18] Two qualitative interview studies have described that women in a prolonged pregnancy may have growing worries about their unborn child's health, and doubts about their body's ability to initiate labour.[19 20] Women also described a lack of information related to late term pregnancy and not being seen by health professionals.[20]

A systematic review of qualitative studies describes that induction of labour can require a shift in expectations as the woman needs to reconsider her original birth plan to a more medicalized one.[16] To our knowledge, there is one previous randomised controlled trial (RCT)[21] from Norway comparing women's experiences of induction of labour at 41 weeks with expectant management (n=508). Women in the expectant management group that had not given birth at 42 weeks and 5 days had an induction of labour. The majority of women in both groups stated, six month after giving birth, that they would prefer to be induced at 41 weeks instead of expectant management in a future prolonged pregnancy.[21] Another RCT compared childbirth experiences, of nulliparous women aged 35 or older, with induction of labour at 39 weeks with expectant management until spontaneous onset or medical indication for induction of labour, using the Childbirth Experience Questionnaire (CEQ) (n=618). No significant differences were found between the groups.[22]



For peer review only - http://bmjopen.bmj.com/site/about/guidelines.xhtml

Induction at or beyond term, compared with expectant management is related to a lower incidence of perinatal death.[23] In a newly published meta-analysis, it was concluded that, induction at 41 weeks compared with expectant management until 42 weeks, overall improved perinatal outcome in women without increasing caesarean delivery rate.[24] Still, induction of labour at 42 weeks was the current standard management in Sweden during the time of this study.

The present study is part of the SWEdish Post Term Induction Study (SWEPIS), a national, multicentre, randomised, controlled, superiority trial.[25] The aim was to evaluate if induction of labour at 41 weeks (n=1381), compared with expectant management and induction of labour at 42 weeks (n=1379), improved perinatal and maternal outcomes. The study was stopped in advance due to ethical reasons related to six cases of perinatal deaths in the expectant management group and none in the induction group. The rates of perinatal mortality and morbidity (primary composite outcome), caesarean deliveries and instrumental vaginal deliveries were similar in both groups.

A woman's childbirth experience is essential for her psychological wellbeing and health. Therefore, the aim of this study was to compare childbirth experiences in women randomly assigned to either induction of labour at 41 weeks or to expectant management and induction of labour at 42 weeks.

# METHODS

## Study design

The study reports on childbirth experience within SWEPIS[25 26], and was performed from May 2016 to October 2018 at 14 Swedish hospitals. The trial was a register based, multicentre, randomised, controlled, superiority trial. It was undertaken with support from the Swedish Network for National Clinical Studies within Obstetrics and Gynaecology (SNAKS, www.snaks.se).

## Participants

Eligible participants were women ≥18 years with an uncomplicated singleton pregnancy, with cephalic presentation at 40 weeks+6 days to 41 weeks+1 day. Women were excluded if they had had a previous caesarean delivery or other uterine surgery, had pregestational or insulin-dependent gestational diabetes, hypertensive disorder of pregnancy, a multiple pregnancy, breech or transverse position of the foetus, diagnosed oligohydramnios, the foetus being small for gestational age or foetal malformation, or contraindications for vaginal delivery.

## Study logistics

The women were given a written leaflet with information about SWEPIS at their regular antenatal check–up at 40 weeks. If interested, they were invited to phone or e-mail the study coordinators, who then gave additional information about the study and booked an appointment at 40 weeks +6 days to 41 weeks +1 day weeks. In the Stockholm Region, women where included during a routine ultrasound scan at 41 weeks. At the visit, the study was further explained, eligibility was confirmed, and written consent to participate was obtained. Randomisation was done online with a module set up by the Swedish Pregnancy Register.[27] Allocation to the trial group, 1:1 was done using central online randomisation by dynamic allocation, to minimise imbalance between the groups. Study centre and parity (primiparity versus multiparity) were used as minimisation variables. All participants gave written informed consent. Women randomised to the induction group were appointed for induction at 41weeks +0 days to 41 weeks +2 days. Women randomised to the



For peer review only - http://bmjopen.bmj.com/site/about/guidelines.xhtml

expectant management group were appointed for induction at 42 weeks +0 days to 42 weeks +1 day if they had not given birth at that time.

## Data collection

### *Main outcome*

Women included at Sahlgrenska University Hospital, Örebro University Hospital and Falun Hospital were asked to complete *The Childbirth Experience Questionnaire Version 2* (CEQ2) three months after birth.[28 29] These three hospitals were chosen with the aim of including different sizes of hospitals in Sweden, as well as urban and rural populations. The CEQ2 was sent through a link via e-mail with two reminders.

### *Exploratory outcome*

Measurement of overall childbirth experience on a *Visual Analogue Scale* (VAS) (1-10) within three days of delivery, is a clinical routine in Sweden and was added as an exploratory outcome variable in women giving birth at the remaining 11 centres (Uppsala University Hospital, Södra Älvsborg Hospital, Region Stockholm [n=5 hospitals], Varberg Hospital, Halmstad Hospital, Visby Hospital and Norra Älvsborg County Hospital). Data on VAS were retrieved from the Swedish Pregnancy Registry.[27]

Data on background variables, pregnancy and delivery outcomes were also retrieved from the Swedish Pregnancy Register.[27]

## Outcome measures

CEQ2 was used as the main outcome measure, and was a pre-specified secondary outcome in SWEPIS. The CEQ2 questionnaire was available in Swedish and English. A VAS (1-10) on the woman's overall childbirth experiences, was added as an exploratory outcome in the hospitals where CEQ2 was not distributed.

## The Childbirth Experience Questionnaire (CEQ)

The first CEQ[30] was developed and validated in Sweden, then translated and culturally adapted to several other languages.[31-34] In the present study, a revised version, CEQ2,[28 29] was used. CEQ2 consists of 22 questions considering four different subscales of the childbirth experience; *Own capacity* (8 items), *Perceived safety* (6 items), *Professional support* (5 items) and *Participation* (3 items), see Table 1. Responses to the items are given on a 4-point Likert scale, ranging from 1 =totally disagree, 2=mostly disagree, 3=mostly agree and 4=totally agree. Three of the items (perceived pain, control and sense of security) are rated on a visual analogue scale (VAS 1-100), which are categorized as 0-40=1, 41-60=2, 61-80=3 and 81-100=4. Negatively worded items are reversed in scoring. Higher scores represent a more positive childbirth experience. The score of each subscale is calculated as a mean of the individual domains. The total CEQ2 score is the mean of the four individual subscale scores (1-4).



For peer review only - http://bmjopen.bmj.com/site/about/guidelines.xhtml

*Table 1* Subscales and items in the Childbirth Experience Questionnaire version 2 (CEQ2)

| |
|---|
| *Own Capacity* |
| Labour and birth went as I had expected. |
| I felt strong during labour and birth. |
| I felt capable during labour and birth. |
| I was tired during labour and birth. |
| I felt happy during labour and birth. |
| I felt that I handled the situation well. |
| As a whole, how painful did you feel childbirth was? |
| As a whole, how much control did you feel you had during childbirth? |
| *Perceived safety* |
| I felt scared during labour and birth. |
| My impression of the team's medical skills made me feel secure. |
| I have many positive memories from childbirth. |
| I have many negative memories from childbirth. |
| Some of my memories from childbirth make me feel depressed. |
| As a whole, how secure did you feel during childbirth? |
| *Professional support* |
| Both my partner and I were treated with warmth and respect. |
| I would have preferred the midwife to be more present during labour and birth. |
| I would have preferred more encouragement from the midwife. |
| The midwife conveyed an atmosphere of calm. |
| The midwife helped me to find my inner strength. |
| *Participation* |
| I wish the staff had listened to me more during labour and birth. |
| I took part in decisions regarding my care and treatment as much as I wanted. |
| I received the information I needed during labour and birth. |

For peer review only

## Overall childbirth experience measured with VAS

As part of the clinical routine in Sweden, a question on overall childbirth experience, with a VAS, was used to assess the women's overall childbirth experience within three days after delivery. It was added as an exploratory outcome and retrieved for women in the hospitals where CEQ was not distributed. Women were asked by a midwife to rate their childbirth experience on a VAS ranging from 1, representing a very negative experience, to 10, representing a very positive experience.[35] When needed, this could be undertaken with the assistance of an interpreter. A rating of 8-10 is considered a very good childbirth experience.[35]

## Data analysis

Mean, standard deviation, median, 25-75 quartiles and 95% confidence intervals were calculated for both CEQ2 and VAS scores. The Mann-Whitney U-test was used to compare the differences in childbirth experience (CEQ2 and VAS) between groups, and Fisher's exact test for categorical variables. Cohen's effect size was used to estimate clinical differences between groups, where an



For peer review only - http://bmjopen.bmj.com/site/about/guidelines.xhtml

effect size of 0.2 was considered small, 0.5 medium and 0.8 as large.[36] All tests were two-sided, using a significance level of 5%. Results were analysed using SPSS, version 25.0 (SPSS Inc., Chicago, IL, USA) or SAS version 9 (SAS Institute, Cary, NC, USA).

## Patient and Public Involvement

Participants and public were not involved in the design of the study or in the conduction of the trial. The study findings will be disseminated to the participants and public through popular science articles.

# RESULTS

In all, 2760 participants were included in SWEPIS. Baseline characteristics were similar in the two treatment groups.[25] In total, 68% (656/959) of women responded to CEQ2, three months after birth and 81% (1457/1801) of women rated their overall childbirth experience on a VAS in the remaining centres. In total, 77% responded to either CEQ2 or overall childbirth experience on a VAS.

## CEQ2

In total, 960 women who were randomised at the three centres: Gothenburg (May 20, 2016 to October 15, 2018), Falun (January 23, 2017 to October 15, 2018) and Örebro (September 9, 2017 to October 15, 2018) were asked to fill in the web questionnaire. One woman in the induction group withdraw her consent to participate before induction of labour, and 22 participants were not fluent in Swedish or English, and thus did not receive the questionnaire. Due to practical and technical issues, another 26 participants did not receive the questionnaire, most often caused by the woman's e-mail address not being registered at randomisation due to technical issues. A total of 78% of women in the induction group and 66% of women in the expectant management group responded to CEQ2 three months after giving birth (Figure 1).

### *Responders and non-responders*

Women who responded to and returned the CEQ2 were more likely to have been born in Sweden (88.6%) compared to women not responding to CEQ2 (73.4%). As well did more women responding to CEQ2 have university education (71.6%) compared to the non-responders (59.1%). Of the women responding to CEQ2, 35.4% had spontaneous onset of labour and 67.1% had induction of labour compared to the non-responders where 47.5% had spontaneous onset of labour and 52.1% had induction of labour (Table 2 and Supplementary material, Table A).

**Table 2. Baseline characteristics for participants responding to CEQ2 and for total population randomised in centres where CEQ2 was sent out.**

| | Participants responding to CEQ | Total CEQ population |
|---|---|---|



For peer review only - http://bmjopen.bmj.com/site/about/guidelines.xhtml

| Variables | Induction group n=354 | Expectant management group n=302 | Induction group n=479 | Expectant management group n=480 |
|---|---|---|---|---|
| **Age at randomisation** (years) | | | | |
| Mean (SD) | 31.3 (5.0) | 31.2 (4.2) | 31.1 (4.9) | 31.1 (4.4) |
| Median (interquartile range) | 31 (28; 35) | 31 (28; 34) | 31 (28; 35) | 31 (28; 34) |
| < 35 years | 271 (76.6%) | 245 (81.1%) | 370 (77.2%) | 384 (80.0%) |
| ≥ 35 years | 83 (23.4%) | 57 (18.9%) | 109 (22.8%) | 96 (20.0%) |
| **Parity (includes stillborn or live births)** | | | | |
| Primiparous | 196 (55.4%) | 164 (54.3%) | 269 (56.2%) | 252 (52.5%) |
| Multiparous | 158 (44.6%) | 138 (45.7%) | 210 (43.8%) | 228 (47.5%) |
| **Smoking at first antenatal visit** | | | | |
| No | 296/302 (98.0%) | 254/259 (97.1%) | 392/404 (97.0%) | 388/400 (97.0%) |
| Yes | 6/302 (2.0%) | 5/259 (1.7%) | 12/404 (3.0%) | 12/400 (3.0%) |
| **BMI at first antenatal visit** | | | | |
| Mean (SD) | 25.2 (5.0) | 25.2 (4.8) | 25.3 (5.0) | 25.4 (5.0) |
| Median (interquartile range) | 24.1 (21.5; 27.7) n=302 | 24.2 (21.8; 27.6) n=267 | 24.1 (21.5; 27.8) n=410 | 24.3 (22.0; 27.7) n=417 |
| **Region of birth** | | | | |
| Sweden | 293/330 (88.8%) | 249/282 (88.3%) | 377/445 (84.7%) | 369/449 (82.2%) |
| Other Nordic countries | 15/330 (4.5%) | 18/282 (6.4%) | 27/445 (6.1%) | 30/449 (6.7%) |
| Europe outside Nordic countries | 6/330 (1.8%) | 4/282 (1.4%) | 7/445 (1.6%) | 6/449 (1.3%) |
| Outside Europe | 16/330 (4.8%) | 14/282 (5.0%) | 34/445 (7.6%) | 44/449 (9.8%) |
| **Highest education** | | | | |
| Primary school ≤ 9 years | 9/310 (2.9%) | 5/264 (1.9%) | 14/412 (3.4%) | 11/419 (2.6%) |
| High school 9 to 12 years | 83/310 (26.8%) | 66/264 (25.0%) | 120/412 (29.1%) | 123/419 (29.4%) |
| University or corresponding | 218/310 (70.3%) | 193/264 (73.1%) | 278/412 (67.5%) | 285/419 (68.0%) |

| | | | | |
|---|---|---|---|---|
| **Gestational age at delivery** (days) | 288.0 (1.0) | 291.7 (2.7) | 288.6 (1.2) | 291.5 (2.7) |
| Mean (SD) | 288 (288; 289) | 292 (289; 294) | 288 (288; 289) | 291 (289; 294) |
| Median (interquartile range) | | | | |
| **Time from admittance to labour ward to delivery** (hours) | | | | |
| Mean (SD) | 20.1 (14.2) | 13.3 (11.0) | 20.3 (14.1) | 13.1 (10.9) |
| Median (interquartile range) | 15.9 (9.5; 27.8) | 10.7 (4.5; 19.3) | 16.4 (9.8; 27.8) | 10.4 (4.6; 19.0) |
| **Onset of birth process** | | | | |
| Spontaneous | 31 (8.8%) | 201 (66.6%) | 49 (10.2%) | 153 (68.1%) |
| Scheduled caesarean delivery | 4 (1.1%) | 0 (0.0%) | 4 (0.8%) | 1 (0.2%) |
| Induction | 319 (90.1%) | 101 (33.4%) | 426 (88.9%) | 151 (31.7%) |
| **Mode of birth** | | | | |
| Spontaneous vaginal | 296 (83.6%) | 256 (84.8%) | 400 (83.5%) | 405 (84.4%) |
| Instrumental vaginal | 24 (6.8%) | 14 (4.6%) | 31 (6.5%) | 26 (5.4%) |
| Caesarean delivery | 34 (9.6%) | 32 (10.6%) | 48 (10.0%) | 49 (10.2%) |
| **Use of epidural anaesthesia** | 199 (56.2%) | 154 (51%) | 286 (59.7%) | 238 (49.6%) |
| **Maternal complications** | | | | |
| Perineal lacerations III-IV | 7 (2.0%) | 4 (1.3%) | 11 (2.3%) | 8 (1.7%) |
| Postpartum haemorrhage (>1000 ml) | 38 (10.7%) | 31 (10.3%) | 54 (11.3%) | 45 (9.4%) |
| Postpartum infection | 11 (3.1%) | 6 (2.0%) | 15 (3.1%) | 6 (1.3%) |
| Preeclampsia/gestational hypertension/eclampsia | 2 (0.6%) | 9 (3.0%) | 2 (0.4%) | 14 (2.9%) |
| **Perinatal complications** | | | | |
| Admittance to neonatal intensive care units (NICU) | 9 (2.5%) | 20 (6.6%) | 14 (2.9%) | 29 (6.0%) |
| Apgar score <7 at 5 min | 3/354 (0.8%) | 5/301 (1.7%) | 5/479 (1.0%) | 5/476 (1.1%) |
| Macrosomia (≥ 4500 g) | 19 (5.4%) | 30 (9.9%) | 2 (5.0%) | 46 (9.6%) |
| **Girls** | 165 (46.6%) | 136 (45.0%) | 222 (46.3%) | 213 (44.4%) |
| **Birth weight (g)** | | | | |
| Mean (SD) | 3811 (430) | 3905 (465) | 3816 (423) | 3897 (459) |
| Median (interquartile range) | 3800 (3530; 4066) | 3898 (3588; 4201) | 3800 (3540; 4084) | 3900 (3580; 4200) |

*Participant characteristics*

For participants responding to CEQ2, the baseline characteristics are presented in Table 2. In total, 90.1% of the women in the induction group had their labour induced, compared with 33.4% of the women in the expectant management group. The gestational age at delivery was on average 3 days longer in the expectant management group. The number of newborns admitted to neonatal intensive care units was 2.5% in the induction group and 6.6% in the expectant management group. The rates of spontaneous vaginal births were similar (83.6% versus 84.8% respectively).

*CEQ2 scores*

The results of the CEQ2 scores are presented for each domain, as well as the total CEQ score, in Table 3. There were no incomplete questionnaires. No significant differences in childbirth experience were identified in three of the four subscales (*Own capacity*, *Perceived safety* and *Professional support)* or in the total score in the CEQ2 between the two randomised groups. There was a significant difference in the subscale *Participation,* where women in the induction group scored slightly higher than women in the expectant management group (p=0.02), with a small effect size (0.19) (Table 3 and Figure 2).

**Table 3. Childbirth experience in the induction group and the expectant management group, according to the Childbirth Experience Questionnaire 2 (CEQ2)**

| | Induction group n=354 | Expectant management group n=302 | Effect size | p-value |
|---|---|---|---|---|
| **Own Capacity** | | | | |
| Mean (SD) | 2.8 (0.6) | 2.7 (0.6) | 0.16 | 0.09 |
| Median (interquartile range) | 2.9 (2.3-3.2) | 2.8 (2.3-3.2) | | |
| (95% CI for mean) | (2.7-2.9) | (2.6-2.8) | | |
| **Perceived Safety** | | | | |
| Mean (SD) | 3.3 (0.7) | 3.2 (0.8) | 0.17 | 0.06 |
| Median (interquartile range) | 3.5 (3.0-3.8) | 3.5 (2.7-3.8) | | |
| (95% CI for mean) | (3.3-3.4) | (3.1-3.3) | | |
| **Professional Support** | | | | |
| Mean (SD) | 3.6 (0.5) | 3.5 (0.6) | 0.09 | 0.38 |
| Median (interquartile range) | 3.8 (3.4-4.0) | 3.8 (3.4-4.0) | | |
| (95% CI for mean) | (3.5-3.6) | (3.4-3.6) | | |
| **Participation** | | | | |
| Mean (SD) | 3.6 (0.6) | 3.4 (0.7) | 0.19 | 0.02 |
| Median (interquartile range) | 4.0 (3.3-4.0) | 3.7 (3.0-4.0) | | |
| (95% CI for mean) | (3.5-3.6) | (3.4-3.5) | | |
| **Total CEQ** | | | | |
| Mean (SD) | 3.3 (0.5) | 3.2 (0.6) | 0.17 | 0.07 |



For peer review only - http://bmjopen.bmj.com/site/about/guidelines.xhtml

| | | | | |
|---|---|---|---|---|
| Median (interquartile range) | 3.5 (3.0-3.7) | 3.4 (2.9-3.7) | | |
| (95% CI for mean) | (3.3-3.4) | (3.2-3.3) | | |

p-values calculated with Mann-Whitney U-test.

## VAS

In total, 1802 women were included at the centres where CEQ2 was not distributed. One woman in the induction group withdrew her consent to participate before induction of labour. A total of 80% of women in the induction group and 82% women in the expectant management group rated their overall childbirth experience on a VAS (1-10) within 3 days of delivery (Supplementary material, Figure A).

### *Responders and non-responders*

The women responding to VAS had similar background characteristics to the women that did not answer VAS. Of women responding to VAS, 82.8% were born in Sweden, 61.6% had a university education, and 58.7% had their labour induced versus 79.9%, 62.0% and 60.5% of the non-responders (Supplementary material, Table B).

### *Participant characteristics*

In the induction group, 83.9% of women had their labour induced, compared with 33.5% of women in the expectant management group. Women in the expectant management group had a pregnancy on average 3 days longer than women in the induction group. There were similar modes of birth in the two groups, 83.2% women had a spontaneous vaginal birth in the induction group, and 82.4% women in the expectant management group (Supplementary Material, Table B).

### *VAS*

There was no significant difference between the induction group and the expectant management group in overall childbirth experience on VAS, or in the number of women rating their childbirth experience as positive (VAS 8-10) (Table 4).

**Table 4. Childbirth experience measured with VAS 1-10**

| | Induction group n=722 | Expectant management group n=735 | Effect size | p-value |
|---|---|---|---|---|
| **VAS** | | | | |
| Mean (SD) | 8.0 (1.2) | 8.1 (2.0) | -0.05 | 0.22 |
| Median (interquartile range) | 8 (7-10) | 9 (7-10) | | |
| (95% CI for Mean) | (7.9-8.2) | (8.0-8.3) | | |
| **VAS 8-10** | 497 (69%) | 528 (72%) | | 0.23 |

For continuous variables, p-values were calculated with the Mann-Whitney U-test.

For categorical variables, p-values were calculated with Pearson Chi-Square.



For peer review only - http://bmjopen.bmj.com/site/about/guidelines.xhtml

For peer review only

# DISCUSSION

The main finding in this study was that there were no significant differences in women's childbirth experiences between women randomised to induction of labour at 41 weeks and women randomised to expectant management and induction of labour at 42 weeks, in terms of the total CEQ2 score, three of four subscales (*Own Capacity, Perceived safety, Professional support)* or according to VAS. However, the women in the induction group scored slightly higher in the domain *Participation* compared with the women in the expectant management group. Women reported an overall positive childbirth experience.

Induction of labour has been reported to have a negative impact on women's childbirth experience.[15-17] However, this was not observed in our study, even though women had their labour induced to a larger extent in the induction group than in the expectant management group. The women had uncomplicated pregnancies and might have been more motivated for induction of labour as they were willing to participate in our study. Furthermore, induction can be perceived as a relief from feelings of discomfort in a prolonged pregnancy[17] and a way to meet the child sooner.[37] Another explanation for the results may be that there were no differences in caesarean deliveries or instrumental vaginal births between the two randomised groups,[25] factors that can have a negative impact on women's childbirth experience.[4 30 38]

None of the subscales *Own Capacity*, *Perceived Safety*, *Professional support* showed significant differences. Most women have expectations of their capacity to give birth and hope they will be able to use their own inherited physical and psychosocial capacities.[39] It is also reported that most women want a vaginal birth, focusing both on safety and psychosocial wellbeing.[39] A safe and supporting environment enables the woman to focus on techniques to manage the birth.[13]

Women in the induction group scored significantly higher in the subscale *Participation* than the expectant management group (3.6 versus 3.4, maximum score 4, p=0.02). In a systematic review of qualitative studies, Downe, et al. [39] found that most women want a physiological labour and birth. However, if interventions were needed, women wanted sufficient information and to be active in the decision-making to retain a sense of personal achievement and control.[39] This may be reflected in the domain *Participation* as the women who participated in the study may have been more motivated for induction of labour, and therefore those allocated to the induction group may have experienced being part of the decision-making to a higher degree than those randomised to the expectant management group (standard care).

The mean total CEQ2 score in the women in the induction group was 3.3, and 3.2 in the expectant management group. This is in agreement with earlier studies using the CEQ2. In the RCT by Walker et al  women at age 35 or older were randomised to either induction of labour at 39 weeks or expectant management until spontaneous onset or medical indication to induce labour (n=618).[22] The women in the induction group scored a total mean CEQ2 score of 3.03 versus 2.96 (p=0.12) in the expectant management group.[22] Also, women with vaginal delivery scored a mean total CEQ2 score of 3.3 in the CEQ2 validation study.[28]

In a retrospective cohort study from Sweden, including all women with a singleton pregnancy, using the VAS for assessment of overall birth experience, 69% (11 493/16 775) of women scored VAS 8-10 (mean 7.94).[15] These scores are similar to the scores in our study, where 69% in the induction group and 72% in the expectant management group scored 8-10 on VAS.



For peer review only - http://bmjopen.bmj.com/site/about/guidelines.xhtml

## Methodological considerations

By using CEQ2[28 30] to compare women's childbirth experiences, we chose an instrument with good psychometric properties.[40] Women could answer the CEQ2 in Swedish or English, which enabled more participants to fill in the questionnaire. The CEQ has been used in several studies in Sweden, and has been shown to have good internal and external validity.[41-44] The advantages of CEQ and CEQ2 are that they measure the multidimensional experience of labour and birth. However, there might be dimensions of the childbirth experience that are difficult to capture with a questionnaire.

A larger proportion of women answered the CEQ2 in the induction group (78%) than in the expectant management group (66%). This may be due to women not perceiving they were participating in a study when they were allocated to the expectant management group, which was standard care at the time. Furthermore, women with spontaneous onset of labour did not answer the questionnaire to the same extent as women whose labour was induced. This difference may also be related to that some women having spontaneous onset of labour perceived that they had not participated in the study. However, these differences in response rate between the randomisation groups were not seen for VAS. This might be explained by the fact that the VAS is used as part of the clinical routine including all women in the participating hospitals.

We added the VAS as an exploratory variable with the purpose of obtaining information on overall childbirth experience in the women at the 11 hospitals were the CEQ2 was not distributed. The reason for CEQ2 not was distributed at all participating centres were due to logistic reasons. The advantage of the VAS is that it is part of the clinical routine and therefore there might be less bias in the women's responses. On the other hand, it is a simplified and non-specific measure, and the women responded to it within three days after the birth, compared to the CEQ which was distributed 3 months after delivery and reflects four domains of childbirth. Hence, the childbirth experience may shift with time when the first wave of relief after the birth has settled. However, the VAS has been shown to correlate to women's overall childbirth experiences and is considered to be useful as a tool to screen for childbirth experience.[15 43 45]

A limitation of the study is that only 22% of eligible women participated in SWEPIS.[25] However, the baseline characteristics were similar for several background variables between the study population in SWEPIS and a Swedish background population of women with uncomplicated pregnancies. However, more women had completed a university education (64% vs. 55%) and were born in Sweden (83% vs. 73%) in SWEPIS compared to the background population.[25] Another limitation is that oral and written information was given by the midwives at a regular antenatal check-up at 40 weeks, but it is unclear how many women actually received the information. The caregiver's attitudes towards the study, as well as the influence of friends and family, might have affected the women's decision to participate.[46 47] Being part of SWEPIS gave the women a chance to have an induction of labour earlier than in routine care in Sweden, this was also expressed by women at randomisation. It is therefore probable that women participating in SWEPIS were more motivated for or positive to induction of labour than women who declined or chose not to contact the study team. If a specific intervention has negative associations, this may be a reason for not participating, and if it is considered favourable, a reason for participating.[48] Another reason for declining could have been that some women wanted a natural birth without interventions. This reason was expressed by women choosing not to be randomised in a RCT in the Netherlands, which aimed to compare the outcome between induction of labour at 41 weeks and expectant management until 42 weeks. In the same study, women who wanted induction reported lower quality of life (EQ-6D), than women preferring expectant management.[49]



For peer review only - http://bmjopen.bmj.com/site/about/guidelines.xhtml

In a qualitative study by Wessberg et al.,[20] women at 41 weeks expressed lack of information about late term pregnancy, showing the importance of clear and good information. This was also expressed in a study by Lou et al.[18] where some women requested more information about their options with late term pregnancy. Women included in SWEPIS, except in Region Stockholm, received an extra visit to a midwife that explained the procedure of induction of labour and thereby got extra detailed information about possible induction procedures. Therefore, the result of this study should be interpreted from this context.

# CONCLUSION

The main result of this study was that there were no significant differences in women's childbirth experiences between women randomised to induction of labour at 41 weeks and women randomised to expectant management and induction of labour at 42 weeks. However, women randomised to induction scored higher on the CEQ2 subscale *Participation* but the difference measured with effect size was small. Overall, women's ratings of their childbirth experience were high.

Further research is needed to describe women's experiences of induction of labour in a prolonged pregnancy, and how the health care system can meet these women's expectations and needs, in order to support them during pregnancy and childbirth.


## Acknowledgments

The authors wish to thank all the women who participated in the study and took their time to answer the questionnaire, and all the midwives and obstetricians at the participating centres. We thank and acknowledge the Swedish Network for National Clinical studies (SNAKS) for their assistance and support.


## Contributions

AW, AD, HH, UBW, VS and HE conceived and designed the study. HN, AW, HH, UBW, HF, JW, AKW and SS oversaw recruitment of study participants and collection of CEQ data at the local centres. UBW and HN did the data cleaning together with statisticians Mattias Molin and Per Ekman. HN compiled and analysed the CEQ2 data. Per Ekman did the analysis regarding VAS. HN wrote the first draft of the manuscript, which was then critically reviewed and revised by all co-authors (HN, AW, AD, HH, UBW, HF, JW, VS, IL, CB, AKW, SS and HE). All authors have approved the final version of the manuscript for submission. All authors have full access to all the data in the study and take responsibility for the integrity of the data and the accuracy of the data analysis. HE is the guarantor.

## Competing interests

None of the authors have any conflict of interest concerning this paper.


## Funding

This study was supported by the Swedish state under the agreement between the Swedish government and the county councils, the ALF-agreement (ALFGBG-440301, ALFGBG-718721,ALFGBG-70940, ALFGBG-426401), the Health Technology Centre at Sahlgrenska University Hospital, the Foundation of the Health and Medical care committee of the Region of Västra




For peer review only - http://bmjopen.bmj.com/site/about/guidelines.xhtml

Götaland, Sweden (VGFOUREG387351, VGFOUREG640891, VGFOUREG854081), Hjalmar Svensson Foundation, the foundation Mary von Sydow, born Wijk donation fund, Uppsala-Örebro regional research council (RFR-556711, RFR-736891), Region Örebro County research committee (OLL-715501), the ALF-agreement in Stockholm (ALF-561222,ALF-562222, ALF-563222), and Centre for Clinical Research Dalarna-Uppsala University, Sweden (CKFUU-417011). The funders had no role in the study design, data collection, data analysis, data interpretation, or writing of the report. The researchers were independent of the funders.

## Ethics approval

This study was approved by the Regional Ethics Board in Gothenburg, Sweden, in May 2014 (Dnr: 285–14) and later complementary applications (T 905-15, T 291-16, T 1180-16, T 330-17, T 1066-17, T 087-18, T 347-18, T 961-18, T 1110-18).

## Consent to participate

Informed written consent was obtained from all women prior to randomisation.

## Data availability statement

Data will be made available, from the corresponding author, upon reasonable request.

## REFERENCES

1. Larkin P, Begley CM, Devane D. Women's experiences of labour and birth: an evolutionary concept analysis. *Midwifery* 2009;25(2):e49-59. doi: 10.1016/j.midw.2007.07.010 [published Online First: 2007/11/13]
2. Lundgren I. Swedish women's experience of childbirth 2 years after birth. *Midwifery* 2005;21(4):346-54. doi: 10.1016/j.midw.2005.01.001 [published Online First: 2005/07/19]
3. Hildingsson I, Karlstrom A, Nystedt A. Women's experiences of induction of labour--findings from a Swedish regional study. *The Australian & New Zealand journal of obstetrics & gynaecology* 2011;51(2):151-7. doi: 10.1111/j.1479-828X.2010.01262.x [published Online First: 2011/04/07]
4. Bossano CM, Townsend KM, Walton AC, et al. The maternal childbirth experience more than a decade after delivery. *American journal of obstetrics and gynecology* 2017;217(3):342.e1-42.e8. doi: 10.1016/j.ajog.2017.04.027 [published Online First: 2017/04/30]
5. Simkin P. Just another day in a woman's life? Women's long-term perceptions of their first birth experience. Part I. *Birth (Berkeley, Calif)* 1991;18(4):203-10. [published Online First: 1991/12/01]
6. Simkin P. Just another day in a woman's life? Part II: Nature and consistency of women's long-term memories of their first birth experiences. *Birth (Berkeley, Calif)* 1992;19(2):64-81. [published Online First: 1992/06/01]
7. Olza I, Leahy-Warren P, Benyamini Y, et al. Women's psychological experiences of physiological childbirth: a meta-synthesis. *BMJ open* 2018;8(10):e020347. doi: 10.1136/bmjopen-2017-020347 [published Online First: 2018/10/21]
8. Bell AF, Andersson E. The birth experience and women's postnatal depression: A systematic review. *Midwifery* 2016;39:112-23. doi: 10.1016/j.midw.2016.04.014 [published Online First: 2016/06/21]
9. Nilsson C, Lundgren I. Women's lived experience of fear of childbirth. *Midwifery* 2009;25(2):e1-9. doi: 10.1016/j.midw.2007.01.017 [published Online First: 2007/05/22]



For peer review only - http://bmjopen.bmj.com/site/about/guidelines.xhtml

10. Dencker A, Nilsson C, Begley C, et al. Causes and outcomes in studies of fear of childbirth: A systematic review. *Women and birth : journal of the Australian College of Midwives* 2019;32(2):99-111. doi: 10.1016/j.wombi.2018.07.004 [published Online First: 2018/08/18]
11. Pang MW, Leung TN, Lau TK, et al. Impact of first childbirth on changes in women's preference for mode of delivery: follow-up of a longitudinal observational study. *Birth (Berkeley, Calif)* 2008;35(2):121-8. doi: 10.1111/j.1523-536X.2008.00225.x [published Online First: 2008/05/30]
12. Hollander MH, van Hastenberg E, van Dillen J, et al. Preventing traumatic childbirth experiences: 2192 women's perceptions and views. *Archives of women's mental health* 2017;20(4):515-23. doi: 10.1007/s00737-017-0729-6 [published Online First: 2017/05/30]
13. Karlstrom A, Nystedt A, Hildingsson I. The meaning of a very positive birth experience: focus groups discussions with women. *BMC pregnancy and childbirth* 2015;15:251. doi: 10.1186/s12884-015-0683-0 [published Online First: 2015/10/11]
14. Henderson J, Redshaw M. Women's experience of induction of labor: a mixed methods study. *Acta obstetricia et gynecologica Scandinavica* 2013;92(10):1159-67. doi: 10.1111/aogs.12211 [published Online First: 2013/07/03]
15. Falk M, Nelson M, Blomberg M. The impact of obstetric interventions and complications on women's satisfaction with childbirth a population based cohort study including 16,000 women. *BMC pregnancy and childbirth* 2019;19(1):494. doi: 10.1186/s12884-019-2633-8 [published Online First: 2019/12/13]
16. Lou S, Hvidman L, Uldbjerg N, et al. Women's experiences of postterm induction of labor: A systematic review of qualitative studies. *Birth (Berkeley, Calif)* 2018 doi: 10.1111/birt.12412 [published Online First: 2018/12/19]
17. Moore JE, Low LK, Titler MG, et al. Moving toward patient-centered care: Women's decisions, perceptions, and experiences of the induction of labor process. *Birth (Berkeley, Calif)* 2014;41(2):138-46. doi: 10.1111/birt.12080 [published Online First: 2014/04/08]
18. Lou S, Carstensen K, Hvidman L, et al. "I guess baby was just too comfy in there…": A qualitative study of women's experiences of elective late-term induction of labour. *Women and birth : journal of the Australian College of Midwives* 2020 doi: 10.1016/j.wombi.2020.03.012 [published Online First: 2020/05/15]
19. Wessberg A, Lundgren I, Elden H. Late-term pregnancy: Navigating in unknown waters - A hermeneutic study. *Women and birth : journal of the Australian College of Midwives* 2019 doi: 10.1016/j.wombi.2019.03.011 [published Online First: 2019/04/06]
20. Wessberg A, Lundgren I, Elden H. Being in limbo: Women's lived experiences of pregnancy at 41 weeks of gestation and beyond - A phenomenological study. *BMC pregnancy and childbirth* 2017;17(1):162. doi: 10.1186/s12884-017-1342-4 [published Online First: 2017/06/06]
21. Heimstad R, Romundstad PR, Hyett J, et al. Women's experiences and attitudes towards expectant management and induction of labor for post-term pregnancy. *Acta obstetricia et gynecologica Scandinavica* 2007;86(8):950-6. doi: 10.1080/00016340701416929 [published Online First: 2007/07/27]
22. Walker KF, Bugg GJ, Macpherson M, et al. Randomized Trial of Labor Induction in Women 35 Years of Age or Older. *N Engl J Med* 2016;374(9):813-22. doi: 10.1056/NEJMoa1509117 [published Online First: 2016/03/11]
23. Middleton P, Shepherd E, Crowther CA. Induction of labour for improving birth outcomes for women at or beyond term. *The Cochrane database of systematic reviews* 2018;5:Cd004945. doi: 10.1002/14651858.CD004945.pub4 [published Online First: 2018/05/10]
24. Alkmark M, Keulen JKJ, Kortekaas JC, et al. Induction of labour at 41 weeks or expectant management until 42 weeks: A systematic review and an individual participant data meta-analysis of randomised trials. *PLoS medicine* 2020;17(12):e1003436. doi: 10.1371/journal.pmed.1003436 [published Online First: 2020/12/09]



For peer review only - http://bmjopen.bmj.com/site/about/guidelines.xhtml

25. Wennerholm U-B, Saltvedt S, Wessberg A, et al. Induction of labour at 41 weeks versus expectant management and induction of labour at 42 weeks (SWEdish Post-term Induction Study, SWEPIS): multicentre, open label, randomised, superiority trial. *BMJ (Clinical research ed)* 2019;367:l6131. doi: 10.1136/bmj.l6131
26. Elden H, Hagberg H, Wessberg A, et al. Study protocol of SWEPIS a Swedish multicentre register based randomised controlled trial to compare induction of labour at 41 completed gestational weeks versus expectant management and induction at 42 completed gestational weeks. *BMC pregnancy and childbirth* 2016;16:49. doi: 10.1186/s12884-016-0836-9 [published Online First: 2016/03/10]
27. Stephansson O, Petersson K, Bjork C, et al. The Swedish Pregnancy Register - for quality of care improvement and research. *Acta obstetricia et gynecologica Scandinavica* 2018;97(4):466-76. doi: 10.1111/aogs.13266 [published Online First: 2017/11/25]
28. Dencker A, Bergqvist L, Berg M, et al. Measuring women's experiences of decision-making and aspects of midwifery support: a confirmatory factor analysis of the revised Childbirth Experience Questionnaire. *BMC pregnancy and childbirth* 2020;20(1):199. doi: 10.1186/s12884-020-02869-0 [published Online First: 2020/04/08]
29. Walker KF, Dencker A, Thornton JG. Childbirth experience questionnaire 2: Validating its use in the United Kingdom. *European journal of obstetrics & gynecology and reproductive biology: X* 2020;5:100097. doi: 10.1016/j.eurox.2019.100097 [published Online First: 2020/02/06]
30. Dencker A, Taft C, Bergqvist L, et al. Childbirth experience questionnaire (CEQ): development and evaluation of a multidimensional instrument. *BMC pregnancy and childbirth* 2010;10:81. doi: 10.1186/1471-2393-10-81 [published Online First: 2010/12/15]
31. Abbaspoor Z, Moghaddam-Banaem L, Ronaghi S, et al. Translation and Cultural Adaptation of the Childbirth Experience Questionnaire (CEQ) in Iran. *Iranian journal of nursing and midwifery research* 2019;24(4):296-300. doi: 10.4103/ijnmr.IJNMR_103_18 [published Online First: 2019/07/25]
32. Soriano-Vidal FJ, Oliver-Roig A, Cabrero-Garcia J, et al. The Spanish version of the Childbirth Experience Questionnaire (CEQ-E): reliability and validity assessment. *BMC pregnancy and childbirth* 2016;16(1):372. doi: 10.1186/s12884-016-1100-z [published Online First: 2016/11/26]
33. Walker KF, Wilson P, Bugg GJ, et al. Childbirth experience questionnaire: validating its use in the United Kingdom. *BMC pregnancy and childbirth* 2015;15:86. doi: 10.1186/s12884-015-0513-4 [published Online First: 2015/04/18]
34. Zhu X, Wang Y, Zhou H, et al. Adaptation of the Childbirth Experience Questionnaire (CEQ) in China: A multisite cross-sectional study. *PloS one* 2019;14(4):e0215373. doi: 10.1371/journal.pone.0215373 [published Online First: 2019/04/25]
35. Graviditetsregistrets Årsrapport 2018 (The Swedish Pregnancy Register yearly report 2018) [Available from: https://www.medscinet.com/GR/uploads/hemsida/dokumentarkiv/2018%20GR%20%C3%A5rsrapport_2.0.pdf.
36. Cohen J. A power primer. *Psychological bulletin* 1992;112(1):155.
37. Gatward H, Simpson M, Woodhart L, et al. Women's experiences of being induced for post-date pregnancy. *Women and birth : journal of the Australian College of Midwives* 2010;23(1):3-9. doi: 10.1016/j.wombi.2009.06.002 [published Online First: 2009/08/04]
38. Nystedt A, Hildingsson I. Women's and men's negative experience of child birth-A cross-sectional survey. *Women and birth : journal of the Australian College of Midwives* 2018;31(2):103-09. doi: 10.1016/j.wombi.2017.07.002 [published Online First: 2017/08/10]
39. Downe S, Finlayson K, Oladapo OT, et al. What matters to women during childbirth: A systematic qualitative review. *PloS one* 2018;13(4):e0194906. doi: 10.1371/journal.pone.0194906 [published Online First: 2018/04/18]



For peer review only - http://bmjopen.bmj.com/site/about/guidelines.xhtml

40. Nilver H, Begley C, Berg M. Measuring women's childbirth experiences: a systematic review for identification and analysis of validated instruments. *BMC pregnancy and childbirth* 2017;17(1):203. doi: 10.1186/s12884-017-1356-y [published Online First: 2017/07/01]
41. Bergqvist L, Dencker A, Taft C, et al. Women's experiences after early versus postponed oxytocin treatment of slow progress in first childbirth--a randomized controlled trial. *Sexual & reproductive healthcare : official journal of the Swedish Association of Midwives* 2012;3(2):61-5. doi: 10.1016/j.srhc.2012.03.003 [published Online First: 2012/05/15]
42. Hildingsson I, Rubertsson C. Childbirth experiences among women with fear of birth randomized to internet-based cognitive therapy or midwife counseling. *Journal of psychosomatic obstetrics and gynaecology* 2019:1-10. doi: 10.1080/0167482x.2019.1634047 [published Online First: 2019/06/28]
43. Turkmen S, Tjernstrom M, Dahmoun M, et al. Post-partum duration of satisfaction with childbirth. *The journal of obstetrics and gynaecology research* 2018;44(12):2166-73. doi: 10.1111/jog.13775 [published Online First: 2018/07/31]
44. Ulfsdottir H, Saltvedt S, Georgsson S. Women's experiences of waterbirth compared with conventional uncomplicated births. *Midwifery* 2019;79:102547. doi: 10.1016/j.midw.2019.102547 [published Online First: 2019/10/15]
45. Larsson C, Saltvedt S, Edman G, et al. Factors independently related to a negative birth experience in first-time mothers. *Sexual & reproductive healthcare : official journal of the Swedish Association of Midwives* 2011;2(2):83-9. doi: 10.1016/j.srhc.2010.11.003 [published Online First: 2011/03/29]
46. Monteiro TM, Katz L, Bento SF, et al. Reasons given by pregnant women for participating in a clinical trial aimed at preventing premature delivery: a qualitative analysis. *BMC pregnancy and childbirth* 2019;19(1):97. doi: 10.1186/s12884-019-2240-8 [published Online First: 2019/03/22]
47. van der Zande ISE, van der Graaf R, Hooft L, et al. Facilitators and barriers to pregnant women's participation in research: A systematic review. *Women and birth : journal of the Australian College of Midwives* 2018;31(5):350-61. doi: 10.1016/j.wombi.2017.12.009 [published Online First: 2018/01/27]
48. Oude Rengerink K, Logtenberg S, Hooft L, et al. Pregnant womens' concerns when invited to a randomized trial: a qualitative case control study. *BMC pregnancy and childbirth* 2015;15:207. doi: 10.1186/s12884-015-0641-x [published Online First: 2015/09/06]
49. Keulen JKJ, Nieuwkerk PT, Kortekaas JC, et al. What women want and why. Women's preferences for induction of labour or expectant management in late-term pregnancy. *Women and birth : journal of the Australian College of Midwives* 2020 doi: 10.1016/j.wombi.2020.03.010 [published Online First: 2020/05/24]

Figure Legends:

Figure 1. Flowchart of the women in the two randomised groups that responded to CEQ2 three months after birth.

Figure 2. Childbirth experience in the induction group and the expectant management group, according to the Childbirth Experience Questionnaire 2 (CEQ2). The line in the box refers to the median and the + refers to the mean.



For peer review only - http://bmjopen.bmj.com/site/about/guidelines.xhtml

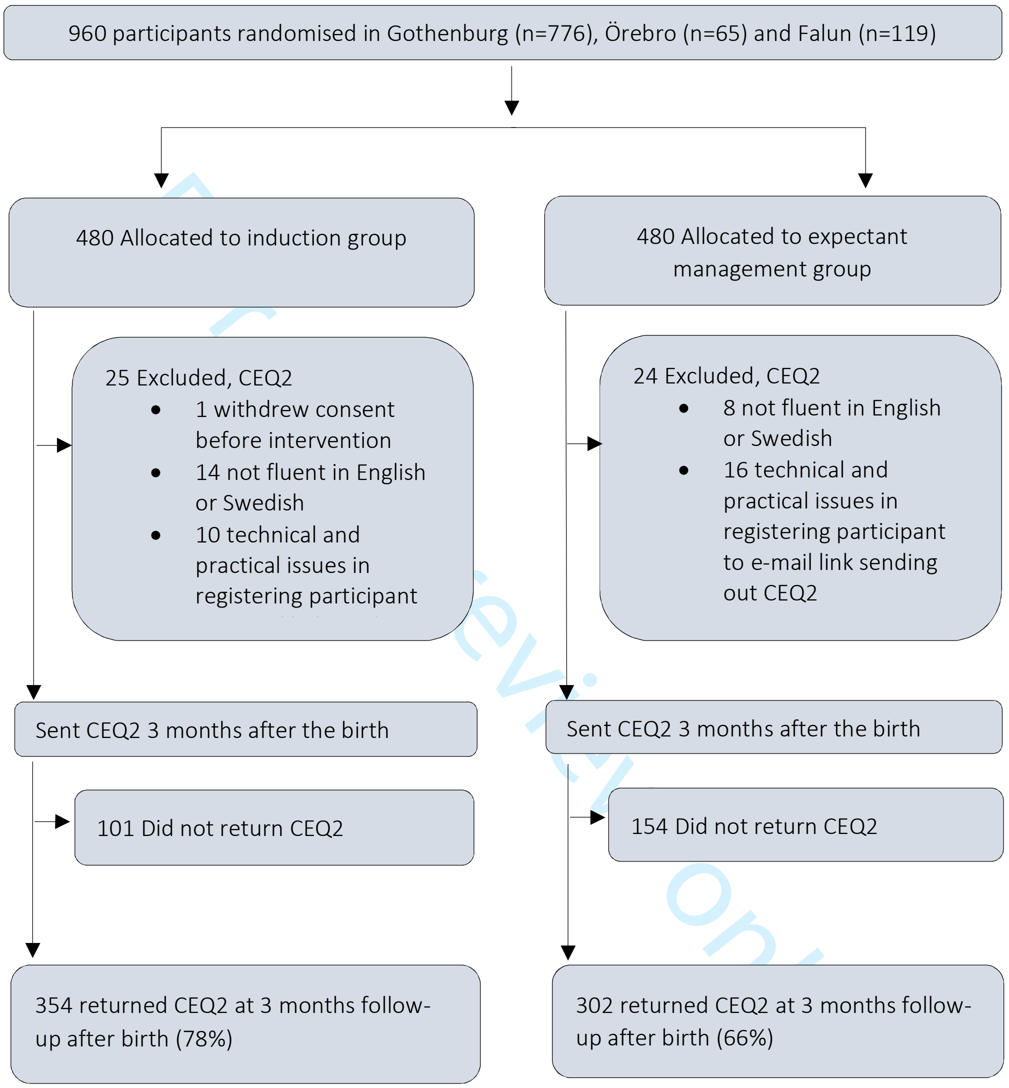


For peer review only - http://bmjopen.bmj.com/site/about/guidelines.xhtml

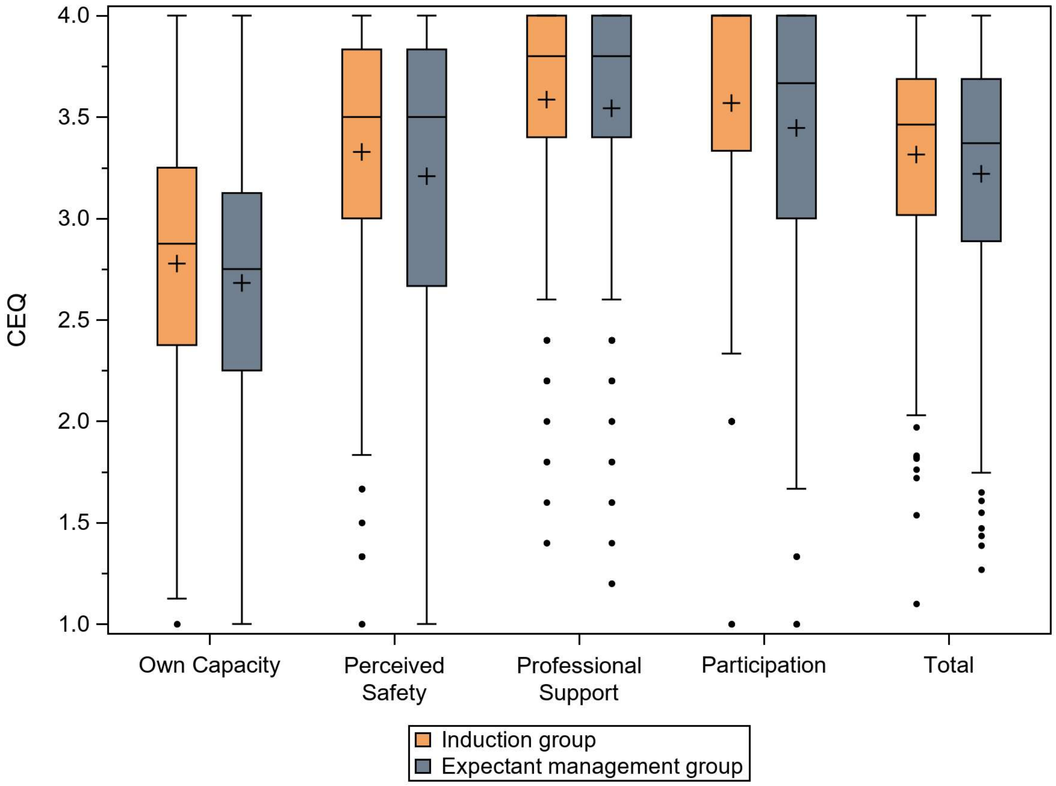
CEQ
4.0
3.5
3.0
2.5
2.0
1.5
1.0
Own Capacity
Perceived Safety
Professional Support
Participation
Total
Induction group
Expectant management group

Supplementary material, Figure A
Flowchart of participants in the two randomised groups rating their overall childbirth experience on a VAS (1-10) within 3 days of delivery

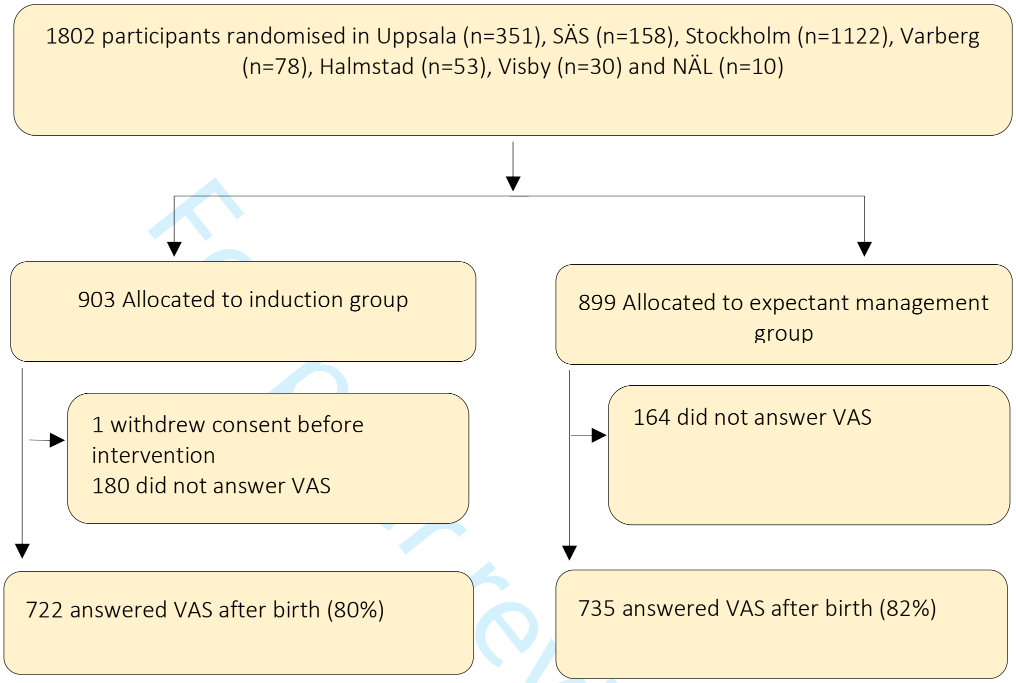

Supplementary material, Table A Baseline characteristics for participants not answering CEQ2

| Variables | Induction group n=125 | Expectant management group n=178 |
|---|---|---|
| Age at randomisation (years) | 30.7 (4.8) | 30.8 (4.8) |
| Mean (SD) | 30 (27; 34) | 30 (27; 35) |
| Median (interquartile range) | | |
| < 35 years | 99 (79.2%) | 139 (78.1%) |
| ≥ 35 years | 26 (20.8%) | 39 (21.9%) |
| Parity (includes stillborn or live births) | | |
| Primiparous | 73 (58.4%) | 88 (49.4%) |
| Multiparous | 52 (41.6%) | 90 (50.6%) |
| Smoking at first antenatal visit | | |
| No | 96/102 (94.1%) | 134/141 (95.0%) |
| Yes | 6/102 (5.9%) | 7/141 (5.0%) |
| BMI at first antenatal visit | 25.4 (5.0) | 25.7 (5.4) |
| Mean (SD) | 23.9 (21.8; 28.3) | 24.4 (22.0; 28.0) |
| Median (interquartile range) | n=108 | n=150 |
| Region of birth | | |
| Sweden | 84/115 (64.2%) | 123/167 (73.7%) |
| Other Nordic countries | 12/115 (10.4&) | 12/167 (7.2%) |
| Europe outside Nordic countries | 1/115 (1.0%) | 2/167 (1.2%) |
| Outside Europe | 18/115 (15.7%) | 12/167 (7.2%) |
| Highest education | | |
| Primary school ≤ 9 years | 5/102 (4.9%) | 6/155 (3.9%) |
| High school 9 to 12 years | 37/102 (36.2%) | 57/155 (36.8%) |
| University or corresponding | 60/102 (58.8%) | 92/155 (59.4%) |
| Gestational age at delivery (days) | 288.7 (1.6) | 291.1 (2.6) |
| Mean (SD) | 288 (288; 289) | 291 (289; 294) |
| Median (interquartile range) | | |
| Time from admittance to labour ward to delivery | 21.1 (13.9) | 12.8 (10.8) |
| (hours) | 18.1 (10.5; 27.8) | 10.1 (5.1; 18.0) |
| Mean (SD) | | |
| Median (interquartile range) | | |
| Onset of birth process | | |
| Spontaneous | 18 (14.4%) | 126 (70.(%) |
| Scheduled caesarean delivery | 0 (0%) | 1 (0.6%) |
| Induction | 107 (85.6%) | 51 (28.7%) |
| Mode of birth | | |
| Spontaneous vaginal | 104 (83.2%) | 149 (83.7%) |
| Instrumental vaginal | 7 (5.6%) | 12 (6.7%) |
| Caesarean delivery | 14 (11.2%) | 17 (9.6%) |
| Use of epidural anaesthesia | 87 (69.6%) | 84 (47.2%) |
| Maternal complications | | |
| Perineal lacerations III-IV | 4 (3.2%) | 4 (2.2%) |
| Postpartum haemorrhage (>1000 ml) | 16 (12.8%) | 14 (7.9%) |
| Postpartum infection | 4 (3.2%) | 0 (0%) |
| Preeclampsia/gestational hypertension/eclampsia | 0 (0%) | 5 (2.8%) |
| Perinatal complications | | |
| Admittance to neonatal intensive care units (NICU) | 5 (4.0%) | 9 (5.1%) |
| Apgar score <7 at 5 min | 2/125 (1.6%) | 0/175 (0.0%) |
| Macrosomia (≥ 4500 g) | 5 (4.0%) | 16 (9.0%) |
| Girls | 57 (45.6%) | 77 (43.3%) |
| Birth weight (g) | 3829 (403) | 3882 (450) |
| Mean (SD) | 3815 (3547; 4102) | 3917 (3563; 4199) |
| Median (interquartile range) | | |

review only

Supplementary material, Table B

Baseline characteristics for participants responding to a VAS (1-10) on overall childbirth experience, not responding to VAS and total VAS population of women participating in SWEPIS.

| | Participants responding to VAS | | Participants not responding to VAS | | Total VAS population | |
|---|---|---|---|---|---|---|
| Variables | Induction group n=722 | Expectant management group n=735 | Induction group n=180 | Expectant management group n=164 | Induction group n=902 | Expectant management group n=899 |
| Age at randomisation (years) | | | 31.5 (4.4) | 31.7 (4.3) | | |
| Mean (SD) | 31.3 (4.7) | 31.3 (4.7) | 31.5 (28; 34) | 31.5 (29; 35) | 31.3 (4.6) | 31.0 (4.6) |
| Median (interquartile range) | 31.2 (28; 35) | 31.2 (28; 35) | | | 31.2 (28; 34.6) | 30.9 (28; 34.2) |
| < 35 years | 567 (78.5%) | 567 (78.5%) | 141 (78.3%) | 127 (77.4%) | 708 (78.5%) | 716 (79.6%) |
| ≥ 35 years | 155 (21.5%) | 155 (21.5%) | 39 (21.7%) | 37 (22.6%) | 194 (21.5%) | 183 (20.4%) |
| Parity (includes stillborn or live births) | | | | | | |
| Primiparous | 402 (55.7%) | 407 (55.4%) | 91 (50.6%) | 94 (57.3%) | 493 (54.7%) | 501 (55.7%) |
| Multiparous | 320 (44.3%) | 328 (44.6%) | 89 (49.4%) | 70 (42.7%) | 402 (45.3%) | 398 (44.3%) |
| Smoking at first antenatal visit | | | | | | |
| No | 678/695 (97.6%) | 686/707 (97.0%) | 172/175 (98.3%) | 154/160 (96.3%) | 850/870 (97.7%) | 840/867 (96.9%) |
| Yes | 17/695 (2.0%) | 21/707 (2.0%) | 3/175 (1.7%) | 6/160 (3.8%) | 20/870 (2.3%) | 27/867 (3.1%) |
| BMI at first antenatal visit | | | | | | |
| Mean (SD) | 24.7 (4.4) | 25.1 (4.9) | 25.2 (5.1) | 24.6 (4.2) | 24.8 (4.6) | 25.0 (4.8) |
| Median (interquartile range) | 23.8 (21.7; 26.7) n=695 | 24 (21.6; 27.5) n=692 | 23.7 (22; 28) n=170 | 23.7 (22; 27) n=156 | 23.8 (21.7; 26.8) n=865 | 23.9 (21.6; 27.2) n=848 |
| Region of birth | | | | | | |
| Sweden | 562/683 (82.3%) | 581/697 (83.4%) | 130/161 (80.7%) | 120/152 (78.9%) | 692/844 (82.0%) | 701/849 (82.6%) |
| Other Nordic countries | 39/683 (5.7%) | 42/697 (6.0%) | 8/161 (5.0%) | 12/152 (7.9%) | 47/844 (5.6%) | 54/849 (6.4%) |
| Europe outside Nordic countries | 10/683 (1.5%) | 9/697 (1.3%) | 3/161 (1.9%) | 3/152 (2.0%) | 13/844 (1.5%) | 12/849 (1.4%) |
| Outside Europe | 72/683 (10.5%) | 65/697 (9.3%) | 20/161 (12.4%) | 17/152 (11.2%) | 92/844 (10.9%) | 82/849 (9.7%) |
| Highest education | | | | | | |



For peer review only - http://bmjopen.bmj.com/site/about/guidelines.xhtml

| | | | | | | |
|---|---|---|---|---|---|---|
| Primary school ≤ 9 years | 31/657 (4.7%) | 37/675 (5.4%) | 9/152 (5.9%) | 9/148 (6.1%) | 40/809 (4.9%) | 46/823 (5.8%) |
| High school 9 to 12 years | 207/657 (31.5%) | 237/675 (35.1%) | 51/152 (33.6%) | 45/148 (30.4%) | 258/809 (31.9%) | 282/823 (34.3%) |
| University or corresponding | 419/657 (63.8%) | 401/675 (59.4%) | 92/152 (60.5%) | 94/148 (63.5%) | 511/809 (63.2%) | 495/823 (60.1%) |
| Gestational age at delivery (days) | | | 289.2 (1.4) | 291.8 (2.7) | | |
| Mean (SD) | 288.9 (1.3) | 291.9 (2.6) | 289 (288; 290) | 292 (290; 295) | 289.0 (1.4) | 291.9 (2.7) |
| Median (interquartile range) | 289 (288; 289) | 292 (290; 294) | | | 289 (288; 289) | 292 (290; 294) |
| Time from admittance to labour ward to delivery (hours) | | | 21.2 (17.4) | 14.1 (12.3) | | |
| Mean (SD) | 19.7 (14.6) | 13.9 (12.9) | 17.1 (9; 29) | 10.1 (5; 20) | 20.0 (15.2) | 13.9 (12.8) |
| Median (interquartile range) | 15.9 (8.8; 27.5)<br>n=721 | 10.4 (4.6; 18.6)<br>n=734 | | | 15.9 (8.8; 27.9)<br>n=901 | 10.4 (4.6; 18.9)<br>n=898 |
| Onset of birth process | | | | | | |
| Spontaneous | 115 (15.9%) | 489 (66.5%) | 31 (17.2%) | 104(63.4%) | 146 (16.2%) | 592 (66.0%) |
| Scheduled caesarean delivery | 1 (0.1%) | 0 (0.0%) | 0 (0.0%) | 1 (0.6%) | 1 (0.1%) | 1/899 (0.1%) |
| Induction | 606 (83.9%) | 246 (33.5%) | 149 (82.8%) | 59 (36.0%) | 755 (83.7%) | 305 (33.9%) |
| Mode of birth | | | | | | |
| Spontaneous vaginal | 601 (83.2%) | 606 (82.4%) | 149 (82.8%) | 129 (78.7%) | 750 (83.1%) | 735 (81.8%) |
| Instrumental vaginal | 46 (6.4%) | 51 (6.9%) | 11 (6.1%) | 14 (8.5%) | 95 (10.5%) | 99 (11.0%) |
| Caesarean delivery | 75 (10.4%) | 78 (10.6%) | 20 (11.1%) | 21 (12.8%) | 57 (6.3%) | 65 (7.2%) |
| Use of epidural anaesthesia | 346 (47.9%) | 345 (46.9%) | 97 (53.9%) | 86 (52.4%) | 443 (49.1%) | 431 (47.9%) |
| Maternal complications | | | | | | |
| Perineal lacerations III-IV | 23 (3.2%) | 34 (4.6%) | 6 (3.3%) | 8 (4.9%) | 29/902 (3.2%) | 42/899 (4.7%) |
| Postpartum haemorrhage (>1000 ml) | 68 (9.4%) | 88 (12%) | 18 (10.0%) | 13 (7.9%) | 86/902 (9.5%) | 101/899 (11.2%) |
| Postpartum infection | 22 (3.0%) | 16 (2.2%) | 5 (2.8%) | 5 (3.0%) | 27/902 (3.0%) | 21/899 (2.3%) |
| Preeclampsia/gestational hypertension/eclampsia | 14 (1.9%) | 26 (3.5%) | 3 (1.7%) | 2 (1.2%) | 17/902 (1.9%) | 28/899 (3.1%) |
| Perinatal complications | | | | | | |
| Admittance to neonatal intensive care units (NICU) | 28 (3.9%) | 34 (4.6%) | 13 (7.2%) | 19 (11.7%) | 41/902 (4.5%) | 53/898 (5.9%) |



For peer review only - http://bmjopen.bmj.com/site/about/guidelines.xhtml

| | | | | | | |
|---|---|---|---|---|---|---|
| Macrosomia (≥ 4500 g) | 37 (5.1%) | 58 (7.9%) | 7 (3.9%) | 10 (6.1%) | 44/902 (4.9%) | 68/898 (7.6%) |
| **Girls** | 305 (42.2%) | 340 (46.3%) | 73/180 (40.6%) | 69/163 (42.3%) | 378/902 (41.9%) | 409/898 (45.5%) |
| **Birth weight (g)** | | | | | | |
| Mean (SD) | 3796 (406) | 3859 (424) | 3891 (373) | 3888 (418) | 3815 (401) | 3865 (423) |
| Median (interquartile range) | 3783 (3500; 4060) | 3845 (3565; 4120) | 3893 (3621; 4175) n=180 | 3900 (3610; 4190) n=163 | 3804 (3530; 4090) n=902 | 3850 (3570; 4128) n=898 |

For peer review only



For peer review only - http://bmjopen.bmj.com/site/about/guidelines.xhtml

# CONSORT 2010 checklist of information to include when reporting a randomised trial*

| Section/Topic | Item No | Checklist item | Reported on page No |
|---|---|---|---|
| **Title and abstract** | | | |
| | 1a | Identification as a randomised trial in the title | yes |
| | 1b | Structured summary of trial design, methods, results, and conclusions (for specific guidance see CONSORT for abstracts) | 2 |
| **Introduction** | | | |
| Background and objectives | 2a | Scientific background and explanation of rationale | 3 |
| | 2b | Specific objectives or hypotheses | 4 |
| **Methods** | | | |
| Trial design | 3a | Description of trial design (such as parallel, factorial) including allocation ratio | 4 |
| | 3b | Important changes to methods after trial commencement (such as eligibility criteria), with reasons | 3 |
| Participants | 4a | Eligibility criteria for participants | 4 |
| | 4b | Settings and locations where the data were collected | 4 |
| Interventions | 5 | The interventions for each group with sufficient details to allow replication, including how and when they were actually administered | 4 |
| Outcomes | 6a | Completely defined pre-specified primary and secondary outcome measures, including how and when they were assessed | 5 |
| | 6b | Any changes to trial outcomes after the trial commenced, with reasons | No changes |
| Sample size | 7a | How sample size was determined | This was a secondary outcome measure |
| | 7b | When applicable, explanation of any interim analyses and stopping guidelines | - |
| Randomisation: | | | |
| Sequence generation | 8a | Method used to generate the random allocation sequence | 4 |
| | 8b | Type of randomisation; details of any restriction (such as blocking and block size) | 4 |
| Allocation concealment mechanism | 9 | Mechanism used to implement the random allocation sequence (such as sequentially numbered containers), describing any steps taken to conceal the sequence until interventions were assigned | 4 |


For peer review only - http://bmjopen.bmj.com/site/about/guidelines.xhtml

| | | | |
|---|---|---|---|
| Implementation | 10 | Who generated the random allocation sequence, who enrolled participants, and who assigned participants to interventions | 4 |
| Blinding | 11a | If done, who was blinded after assignment to interventions (for example, participants, care providers, those assessing outcomes) and how | - |
| | 11b | If relevant, description of the similarity of interventions | - |
| Statistical methods | 12a | Statistical methods used to compare groups for primary and secondary outcomes | 6 |
| | 12b | Methods for additional analyses, such as subgroup analyses and adjusted analyses | 6 |
| **Results** | | | |
| Participant flow (a diagram is strongly recommended) | 13a | For each group, the numbers of participants who were randomly assigned, received intended treatment, and were analysed for the primary outcome | 6 |
| | 13b | For each group, losses and exclusions after randomisation, together with reasons | 7 |
| Recruitment | 14a | Dates defining the periods of recruitment and follow-up | 6 |
| | 14b | Why the trial ended or was stopped | 3 |
| Baseline data | 15 | A table showing baseline demographic and clinical characteristics for each group | 7 |
| Numbers analysed | 16 | For each group, number of participants (denominator) included in each analysis and whether the analysis was by original assigned groups | 7 |
| Outcomes and estimation | 17a | For each primary and secondary outcome, results for each group, and the estimated effect size and its precision (such as 95% confidence interval) | 8 |
| | 17b | For binary outcomes, presentation of both absolute and relative effect sizes is recommended | - |
| Ancillary analyses | 18 | Results of any other analyses performed, including subgroup analyses and adjusted analyses, distinguishing pre-specified from exploratory | - |
| Harms | 19 | All important harms or unintended effects in each group (for specific guidance see CONSORT for harms) | - |
| **Discussion** | | | |
| Limitations | 20 | Trial limitations, addressing sources of potential bias, imprecision, and, if relevant, multiplicity of analyses | 10-11 |
| Generalisability | 21 | Generalisability (external validity, applicability) of the trial findings | 11 |
| Interpretation | 22 | Interpretation consistent with results, balancing benefits and harms, and considering other relevant evidence | 9-10 |
| **Other information** | | | |
| Registration | 23 | Registration number and name of trial registry | 2 |
| Protocol | 24 | Where the full trial protocol can be accessed, if available | - |
| Funding | 25 | Sources of funding and other support (such as supply of drugs), role of funders | 12 |

For peer review only


For peer review only - http://bmjopen.bmj.com/site/about/guidelines.xhtml

*We strongly recommend reading this statement in conjunction with the CONSORT 2010 Explanation and Elaboration for important clarifications on all the items. If relevant, we also recommend reading CONSORT extensions for cluster randomised trials, non-inferiority and equivalence trials, non-pharmacological treatments, herbal interventions, and pragmatic trials. Additional extensions are forthcoming: for those and for up to date references relevant to this checklist, see www.consort-statement.org.

For peer review only

BMJ Open

# Women's childbirth experience in the Swedish Post-term Induction Study (SWEPIS): a multicentre, randomised, controlled trial



| | |
|---|---|
| Journal: | *BMJ Open* |
| Manuscript ID | bmjopen-2020-042340.R2 |
| Article Type: | Original research |
| Date Submitted by the Author: | 14-Mar-2021 |





| | |
|---|---|
| Complete List of Authors: | Nilvér, Helena; Göteborgs Universitet, Institution of Helath and Care Science<br>Wessberg, Anna; Goteborgs universitet Sahlgrenska Akademin, Institute of Helath and Care Sciense; Sahlgrenska universitetssjukhuset, Department of Obstetrics<br>Dencker, Anna; Goteborgs universitet Sahlgrenska Akademin, Institute of Health and Care Science<br>Hagberg, Henrik; Goteborgs universitet Sahlgrenska Akademin, Institute of Clinical Sciences, Centre of Perinatal Medicine & Health ; Sahlgrenska University Hospital, Department of Obstertics<br>Wennerholm, Ulla-Britt; Goteborgs universitet Sahlgrenska Akademin, Institute of Clinical Sciences, Centre of Perinatal Medicine &Health; Sahlgrenska University Hospital, Department of obstertics<br>Fadl, Helena; Örebro Universitet Institutionen för Medicinska Vetenskaper,<br>Wesström, Jan; Dalarna County Council, Centre for Clinical Research, Department of Women's health<br>Sengpiel, Verena; Goteborgs universitet Sahlgrenska Akademin, Institute of Clinical Science, Centre of Perinatal Medicine Health; Sahlgrenska University Hospital, Department of Obstetrics<br>Lundgren, Ingela; Goteborgs universitet Sahlgrenska Akademin, Institute of Health and Care Science; Sahlgrenska University Hospital, Department of Obstetrics<br>Bergh, Christina; Goteborgs Universitet, Institute of Clinical Sciences; Sahlgrenska universitetssjukhuset, Reproductive Medicine<br>Wikström, Anna-Karin; Uppsala University, Department of Women's and Children's Health<br>Saltvedt, Sissel; Karolinska University Hospital, Division of Obstetrics and Gynaecology; Karolinska Institutet, Department of Women's and Children's Health<br>Elden, Helen; Goteborgs universitet Sahlgrenska Akademin, Institution of Health and Care Sciences; Sahlgrenska University Hospital, Department of Obstertics |





| | |
|---|---|
| <b>Primary Subject Heading</b>: | Obstetrics and gynaecology |
| Secondary Subject Heading: | Obstetrics and gynaecology |
| Keywords: | OBSTETRICS, Maternal medicine < OBSTETRICS, REPRODUCTIVE |



For peer review only - http://bmjopen.bmj.com/site/about/guidelines.xhtml

| | MEDICINE |
|---|---|
| | |

SCHOLARONE™
Manuscripts

*I, the Submitting Author has the right to grant and does grant on behalf of all authors of the Work (as defined in the below author licence), an exclusive licence and/or a non-exclusive licence for contributions from authors who are: i) UK Crown employees; ii) where BMJ has agreed a CC-BY licence shall apply, and/or iii) in accordance with the terms applicable for US Federal Government officers or employees acting as part of their official duties; on a worldwide, perpetual, irrevocable, royalty-free basis to BMJ Publishing Group Ltd ("BMJ") its licensees and where the relevant Journal is co-owned by BMJ to the co-owners of the Journal, to publish the Work in this journal and any other BMJ products and to exploit all rights, as set out in our licence.*

*The Submitting Author accepts and understands that any supply made under these terms is made by BMJ to the Submitting Author unless you are acting as an employee on behalf of your employer or a postgraduate student of an affiliated institution which is paying any applicable article publishing charge ("APC") for Open Access articles. Where the Submitting Author wishes to make the Work available on an Open Access basis (and intends to pay the relevant APC), the terms of reuse of such Open Access shall be governed by a Creative Commons licence – details of these licences and which Creative Commons licence will apply to this Work are set out in our licence referred to above.*

*Other than as permitted in any relevant BMJ Author's Self Archiving Policies, I confirm this Work has not been accepted for publication elsewhere, is not being considered for publication elsewhere and does not duplicate material already published. I confirm all authors consent to publication of this Work and authorise the granting of this licence.*

For peer review only

# Women's childbirth experience in the Swedish Post-term Induction Study (SWEPIS): a multicentre, randomised, controlled trial

Helena Nilvér,[1] Anna Wessberg,[1,3] Anna Dencker,[1] Henrik Hagberg,[2,3] Ulla-Britt Wennerholm,[2,3] Helena Fadl,[4] Jan Wesström,[5] Verena Sengpiel,[2,3] Ingela Lundgren,[1,3] Christina Bergh,[2,6] Anna-Karin Wikström,[7] Sissel Saltvedt,[8] Helen Elden[1,3]

[1]Institute of Health and Care Sciences, Sahlgrenska Academy, University of Gothenburg, Gothenburg, Sweden

[2]Centre of Perinatal Medicine & Health, Department of Obstetrics and gynaecology, Institute of Clinical Sciences, Sahlgrenska Academy, Gothenburg, Sweden

[3]Department of Obstetrics, Sahlgrenska University Hospital, Region Västra Götaland, Gothenburg, Sweden

[4]Department of Obstetrics and Gynaecology, Faculty of Medicine and Health, Örebro University, Örebro, Sweden

[5]Department of Women's and Children's Health, Uppsala University, Uppsala, Sweden; Centre for Clinical Research Dalarna, Falun, Sweden

[6]Reproductive Medicine, Sahlgrenska University hospital, Gothenburg, Sweden

[7]Department of Women's and Children's Health, Uppsala University, Uppsala, Sweden

[8]Department of Women's and Children's Health, Karolinska Institute, Karolinska University Hospital, Stockholm, Sweden

Corresponding author:
Helena Nilvér
Institute of Health and Care Sciences
Sahlgrenska Academy
University of Gothenburg
Box 457
Gothenburg SE-405 30
Sweden
Helena.nilver@gu.se

For peer review only



For peer review only - http://bmjopen.bmj.com/site/about/guidelines.xhtml

# ABSTRACT

## Objective

To compare childbirth experiences in women randomly assigned to either induction of labour at 41 weeks or to expectant management until 42 weeks in the Swedish Post-term Induction Study (SWEPIS).

## Design

A register based, multicentre, randomised, controlled superiority trial.

## Setting

Women were recruited at 14 hospitals in Sweden, 2016 - 2018.

## Participants

Women with an uncomplicated singleton pregnancy were recruited at 41 gestational weeks.

## Interventions

The women were randomly assigned to induction of labour at 41 weeks (induction group, n=1381), or expectant management until 42 weeks (expectant management group, n=1379).

## Outcome measures

As main outcome, women's childbirth experiences were measured using the Childbirth Experience Questionnaire version 2 (CEQ2), in 656 women, three months after the birth at three hospitals. As exploratory outcome, overall childbirth experience was measured in 1457 women using a Visual Analogue Scale (VAS 1-10) within three days after delivery at the remaining eleven hospitals.

## Results

The total response rate was 77% (2113/2760). There were no significant differences in childbirth experience measured with CEQ2 between the groups (induction group n=354, expectant management group n=302) in the subscales; *Own capacity* (2.8 versus 2.7, p=0.09), *Perceived safety* (3.3 versus 3.2, p=0.06) and *Professional support* (3.6 versus 3.5, p=0.38) or in the total CEQ2 score (3.3 versus 3.2, p=0.07) respectively. Women in the induction group scored higher in the subscale *Participation* (3.6 versus 3.4, p=0.02), although with a small effect size (0.19). No significant difference was observed in overall childbirth experience according to VAS (8.0 (n=735) versus 8.1 (n=735), p=0.22).

## Conclusions

There were no differences in childbirth experience according to CEQ2 or overall childbirth experience assessed with VAS between women randomly assigned to induction of labour at 41 weeks or expectant management until 42 weeks. Overall, women rated their childbirth experiences high.

## Trial registration

Current Controlled Trials, ISRCTN26113652.


For peer review only

**KEYWORDS**

Childbirth experience, Childbirth Experience Questionnaire, induction of labour, prolonged pregnancy, randomised controlled trial

For peer review only

## STRENGTH AND LIMITATIONS OF THIS STUDY

- The study had a high total response rate.
- The study was based on a large randomised controlled trial.
- Childbirth experience was evaluated with a validated questionnaire (CEQ2) 3 months after birth.
- The questionnaire (CEQ2) was used in only three of 14 participating centres; other centres evaluated the overall childbirth experience with a visual analogue scale (VAS) as an exploratory outcome.
- The response rate with CEQ2 was higher in the induction group than in the expectant management group.

For peer review only

## INTRODUCTION

Women's childbirth experiences are individual, complex life events, which are related to the outcome for the mother and child.[1-3] These experiences may leave long lasting impressions, as women tend to remember their childbirth experiences very well.[4-6] A positive birth experience can strengthen the woman and be an empowering life event[5 7] while a negative experience can increase the risk of postpartum depression,[8] fear of childbirth[9 10] and future fear of vaginal births.[11] Women with traumatic childbirth experiences have identified the lack and loss of control, and insufficient practical and emotional support, as the main sources of their traumatic experiences.[12] A qualitative study on women's positive childbirth experiences showed that being in a safe and supporting environment gave them a sense of control, and made it possible to focus on strategies to manage childbirth. Knowledge about the birth process was also important, helping the women to perceive control and enable them to take part in decision-making.[13]

Earlier studies have reported induction of labour being associated with a more negative childbirth experience than spontaneous onset of labour.[3 14 15] However, in prolonged pregnancies, this difference was not as obvious.[3 16] Induction of labour in prolonged pregnancies can relieve some women from feelings of discomfort, and the uncertainty of not knowing when and where labour will begin.[17] Women's trust in health professionals as well as an impatience to deliver a healthy child can make women to choose and accept induction of labour.[18] Two qualitative interview studies have described that women in a prolonged pregnancy may have growing worries about their unborn child's health, and doubts about their body's ability to initiate labour.[19 20] Women also described a lack of information related to late term pregnancy and not being seen by health professionals.[20]

A systematic review of qualitative studies describes that induction of labour can require a shift in expectations as the woman needs to reconsider her original birth plan to a more medicalized one.[16] To our knowledge, there is one previous randomised controlled trial (RCT)[21] from Norway comparing women's experiences of induction of labour at 41 weeks with expectant management (n=508). Women in the expectant management group that had not given birth at 42 weeks and 5 days had an induction of labour. The majority of women in both groups stated, six month after giving birth, that they would prefer to be induced at 41 weeks instead of expectant management in a future prolonged pregnancy.[21] Another RCT compared childbirth experiences, of nulliparous women aged 35 or older, with induction of labour at 39 weeks with expectant management until spontaneous onset or medical indication for induction of labour, using the Childbirth Experience Questionnaire (CEQ) (n=618). No significant differences were found between the groups.[22]

Induction at or beyond term, compared with expectant management is related to a lower incidence of perinatal death.[23] In a newly published meta-analysis, it was concluded that, induction at 41 weeks compared with expectant management until 42 weeks, overall improved perinatal outcome in women without increasing caesarean delivery rate.[24] Still, induction of labour at 42 weeks was the current standard management in Sweden during the time of this study.

The present study is part of the SWEdish Post Term Induction Study (SWEPIS), a national, multicentre, randomised, controlled, superiority trial.[25] The aim was to evaluate if induction of labour at 41 weeks (n=1381), compared with expectant management and induction of labour at 42 weeks (n=1379), improved perinatal and maternal outcomes. The study was stopped in advance due to ethical reasons related to six cases of perinatal deaths in the expectant management group and none in the induction group. The rates of perinatal mortality and morbidity (primary composite outcome), caesarean deliveries and instrumental vaginal deliveries were similar in both groups.

A woman's childbirth experience is essential for her psychological wellbeing and health. Therefore, the aim of this study was to compare childbirth experiences in women randomly assigned to either induction of labour at 41 weeks or to expectant management and induction of labour at 42 weeks.

# METHODS

## Study design

The study reports on childbirth experience within SWEPIS[25 26], and was performed from May 2016 to October 2018 at 14 Swedish hospitals. The trial was a register based, multicentre, randomised, controlled, superiority trial. It was undertaken with support from the Swedish Network for National Clinical Studies within Obstetrics and Gynaecology (SNAKS, www.snaks.se).

## Participants

Eligible participants were women ≥18 years with an uncomplicated singleton pregnancy, with cephalic presentation at 40 weeks+6 days to 41 weeks+1 day. Women were excluded if they had had a previous caesarean delivery or other uterine surgery, had pregestational or insulin-dependent gestational diabetes, hypertensive disorder of pregnancy, a multiple pregnancy, breech or transverse position of the foetus, diagnosed oligohydramnios, the foetus being small for gestational age or foetal malformation, or contraindications for vaginal delivery.

## Study logistics

The women were given a written leaflet with information about SWEPIS at their regular antenatal check–up at 40 weeks. If interested, they were invited to phone or e-mail the study coordinators, who then gave additional information about the study and booked an appointment at 40 weeks +6 days to 41 weeks +1 day weeks. In the Stockholm Region, women where included during a routine ultrasound scan at 41 weeks. At the visit, the study was further explained, eligibility was confirmed, and written consent to participate was obtained. Randomisation was done online with a module set up by the Swedish Pregnancy Register.[27] Allocation to the trial group, 1:1 was done using central online randomisation by dynamic allocation, to minimise imbalance between the groups. Study centre and parity (primiparity versus multiparity) were used as minimisation variables. All participants gave written informed consent. Women randomised to the induction group were appointed for induction at 41weeks +0 days to 41 weeks +2 days. Women randomised to the



For peer review only - http://bmjopen.bmj.com/site/about/guidelines.xhtml

expectant management group were appointed for induction at 42 weeks +0 days to 42 weeks +1 day if they had not given birth at that time.

## Outcome measures

*The Childbirth Experience Questionnaire Version 2* (CEQ2) was used as the main outcome measure, and was a pre-specified secondary outcome in SWEPIS. The CEQ2 questionnaire was available in Swedish and English. A *Visual Analogue Scale* (VAS) (1-10) on the woman's overall childbirth experiences, was added as an exploratory outcome in the hospitals where CEQ2 was not distributed.

## Data collection

### *Main outcome*

Women included at Sahlgrenska University Hospital, Örebro University Hospital and Falun Hospital were asked to complete the CEQ2 three months after birth.[28 29] These three hospitals were chosen with the aim of including different sizes of hospitals in Sweden, as well as urban and rural populations. The CEQ2 was sent through a link via e-mail with two reminders.

### *Exploratory outcome*

Measurement of overall childbirth experience on a VAS (1-10) within three days of delivery, is a clinical routine in Sweden and was added as an exploratory outcome variable in women giving birth at the remaining 11 centres (Uppsala University Hospital, Södra Älvsborg Hospital, Region Stockholm [n=5 hospitals], Varberg Hospital, Halmstad Hospital, Visby Hospital and Norra Älvsborg County Hospital). Data on VAS were retrieved from the Swedish Pregnancy Registry.[27]

Data on background variables, pregnancy and delivery outcomes were also retrieved from the Swedish Pregnancy Register.[27]

## The Childbirth Experience Questionnaire (CEQ)

The first CEQ[30] was developed and validated in Sweden, then translated and culturally adapted to several other languages.[31-34] In the present study, a revised version, CEQ2,[28 29] was used. CEQ2 consists of 22 questions considering four different subscales of the childbirth experience; *Own capacity* (8 items), *Perceived safety* (6 items), *Professional support* (5 items) and *Participation* (3 items), see Table 1. Responses to the items are given on a 4-point Likert scale, ranging from 1 =totally disagree, 2=mostly disagree, 3=mostly agree and 4=totally agree. Three of the items (perceived pain, control and sense of security) are rated on a visual analogue scale (VAS 1-100), which are categorized as 0-40=1, 41-60=2, 61-80=3 and 81-100=4. Negatively worded items are reversed in scoring. Higher scores represent a more positive childbirth experience. The score of each subscale is calculated as a mean of the individual domains. The total CEQ2 score is the mean of the four individual subscale scores (1-4).



For peer review only - http://bmjopen.bmj.com/site/about/guidelines.xhtml

*Table 1* Subscales and items in the Childbirth Experience Questionnaire version 2 (CEQ2)

| |
|---|
| *Own Capacity* |
| Labour and birth went as I had expected. |
| I felt strong during labour and birth. |
| I felt capable during labour and birth. |
| I was tired during labour and birth. |
| I felt happy during labour and birth. |
| I felt that I handled the situation well. |
| As a whole, how painful did you feel childbirth was? |
| As a whole, how much control did you feel you had during childbirth? |
| *Perceived safety* |
| I felt scared during labour and birth. |
| My impression of the team's medical skills made me feel secure. |
| I have many positive memories from childbirth. |
| I have many negative memories from childbirth. |
| Some of my memories from childbirth make me feel depressed. |
| As a whole, how secure did you feel during childbirth? |
| *Professional support* |
| Both my partner and I were treated with warmth and respect. |
| I would have preferred the midwife to be more present during labour and birth. |
| I would have preferred more encouragement from the midwife. |
| The midwife conveyed an atmosphere of calm. |
| The midwife helped me to find my inner strength. |
| *Participation* |
| I wish the staff had listened to me more during labour and birth. |
| I took part in decisions regarding my care and treatment as much as I wanted. |
| I received the information I needed during labour and birth. |

For peer review only

## Overall childbirth experience measured with VAS

As part of the clinical routine in Sweden, a question on overall childbirth experience, with a VAS, was used to assess the women's overall childbirth experience within three days after delivery. It was added as an exploratory outcome and retrieved for women in the hospitals where CEQ was not distributed. Women were asked by a midwife to rate their childbirth experience on a VAS ranging from 1, representing a very negative experience, to 10, representing a very positive experience.[35] When needed, this could be undertaken with the assistance of an interpreter. A rating of 8-10 is considered a very good childbirth experience.[35]

## Data analysis

Mean, standard deviation, median, 25-75 quartiles and 95% confidence intervals were calculated for both CEQ2 and VAS scores. The Mann-Whitney U-test was used to compare the differences in childbirth experience (CEQ2 and VAS) between groups, and Fisher's exact test for categorical variables. Cohen's effect size was used to estimate clinical differences between groups, where an



For peer review only - http://bmjopen.bmj.com/site/about/guidelines.xhtml

effect size of 0.2 was considered small, 0.5 medium and 0.8 as large.[36] All tests were two-sided, using a significance level of 5%. Results were analysed using SPSS, version 25.0 (SPSS Inc., Chicago, IL, USA) or SAS version 9 (SAS Institute, Cary, NC, USA).

## Patient and Public Involvement

Participants and public were not involved in the design of the study or in the conduction of the trial. The study findings will be disseminated to the participants and public through popular science articles.

# RESULTS

In all, 2760 participants were included in SWEPIS. Baseline characteristics were similar in the two treatment groups.[25] In total, 68% (656/959) of women responded to CEQ2, three months after birth and 81% (1457/1801) of women rated their overall childbirth experience on a VAS in the remaining centres. In total, 77% responded to either CEQ2 or overall childbirth experience on a VAS.

## CEQ2

In total, 960 women who were randomised at the three centres: Gothenburg (May 20, 2016 to October 15, 2018), Falun (January 23, 2017 to October 15, 2018) and Örebro (September 9, 2017 to October 15, 2018) were asked to fill in the web questionnaire. One woman in the induction group withdrew her consent to participate before induction of labour, and 22 participants were not fluent in Swedish or English, and thus did not receive the questionnaire. Due to practical and technical issues, another 26 participants did not receive the questionnaire, most often caused by the woman's e-mail address not being registered at randomisation due to technical issues. A total of 78% of women in the induction group and 66% of women in the expectant management group responded to CEQ2 three months after giving birth (Figure 1).

### *Responders and non-responders*

Women who responded to and returned the CEQ2 were more likely to have been born in Sweden (88.6%), compared to women who did not respond to CEQ2 (73.4%). More women responding to CEQ2 had a university education (71.6%), compared to the non-responders (59.1%). Of the women responding to CEQ2, 35.4% had spontaneous onset of labour and 67.1% had induction of labour compared to the non-responders where 47.5% had spontaneous onset of labour and 52.1% had induction of labour (Table 2 and Supplementary material, Table A).

**Table 2. Baseline characteristics for participants responding to CEQ2 and for total population randomised in centres where CEQ2 was sent out.**

| | Participants responding to CEQ | Total CEQ population |
|---|---|---|



For peer review only - http://bmjopen.bmj.com/site/about/guidelines.xhtml

| Variables | Induction group n=354 | Expectant management group n=302 | Induction group n=479 | Expectant management group n=480 |
|---|---|---|---|---|
| **Age at randomisation** (years) | | | | |
| Mean (SD) | 31.3 (5.0) | 31.2 (4.2) | 31.1 (4.9) | 31.1 (4.4) |
| Median (interquartile range) | 31 (28; 35) | 31 (28; 34) | 31 (28; 35) | 31 (28; 34) |
| < 35 years | 271 (76.6%) | 245 (81.1%) | 370 (77.2%) | 384 (80.0%) |
| ≥ 35 years | 83 (23.4%) | 57 (18.9%) | 109 (22.8%) | 96 (20.0%) |
| **Parity (includes stillborn or live births)** | | | | |
| Primiparous | 196 (55.4%) | 164 (54.3%) | 269 (56.2%) | 252 (52.5%) |
| Multiparous | 158 (44.6%) | 138 (45.7%) | 210 (43.8%) | 228 (47.5%) |
| **Smoking at first antenatal visit** | | | | |
| No | 296/302 (98.0%) | 254/259 (97.1%) | 392/404 (97.0%) | 388/400 (97.0%) |
| Yes | 6/302 (2.0%) | 5/259 (1.7%) | 12/404 (3.0%) | 12/400 (3.0%) |
| **BMI at first antenatal visit** | | | | |
| Mean (SD) | 25.2 (5.0) | 25.2 (4.8) | 25.3 (5.0) | 25.4 (5.0) |
| Median (interquartile range) | 24.1 (21.5; 27.7) n=302 | 24.2 (21.8; 27.6) n=267 | 24.1 (21.5; 27.8) n=410 | 24.3 (22.0; 27.7) n=417 |
| **Region of birth** | | | | |
| Sweden | 293/330 (88.8%) | 249/282 (88.3%) | 377/445 (84.7%) | 369/449 (82.2%) |
| Other Nordic countries | 15/330 (4.5%) | 18/282 (6.4%) | 27/445 (6.1%) | 30/449 (6.7%) |
| Europe outside Nordic countries | 6/330 (1.8%) | 4/282 (1.4%) | 7/445 (1.6%) | 6/449 (1.3%) |
| Outside Europe | 16/330 (4.8%) | 14/282 (5.0%) | 34/445 (7.6%) | 44/449 (9.8%) |
| **Highest education** | | | | |
| Primary school ≤ 9 years | 9/310 (2.9%) | 5/264 (1.9%) | 14/412 (3.4%) | 11/419 (2.6%) |
| High school 9 to 12 years | 83/310 (26.8%) | 66/264 (25.0%) | 120/412 (29.1%) | 123/419 (29.4%) |
| University or corresponding | 218/310 (70.3%) | 193/264 (73.1%) | 278/412 (67.5%) | 285/419 (68.0%) |

| | | | | |
|---|---|---|---|---|
| **Gestational age at delivery** (days) | | | | |
| Mean (SD) | 288.0 (1.0) | 291.7 (2.7) | 288.6 (1.2) | 291.5 (2.7) |
| Median (interquartile range) | 288 (288; 289) | 292 (289; 294) | 288 (288; 289) | 291 (289; 294) |
| **Time from admittance to labour ward to delivery** (hours) | | | | |
| Mean (SD) | 20.1 (14.2) | 13.3 (11.0) | 20.3 (14.1) | 13.1 (10.9) |
| Median (interquartile range) | 15.9 (9.5; 27.8) | 10.7 (4.5; 19.3) | 16.4 (9.8; 27.8) | 10.4 (4.6; 19.0) |
| **Onset of birth process** | | | | |
| Spontaneous | 31 (8.8%) | 201 (66.6%) | 49 (10.2%) | 153 (68.1%) |
| Scheduled caesarean delivery | 4 (1.1%) | 0 (0.0%) | 4 (0.8%) | 1 (0.2%) |
| Induction | 319 (90.1%) | 101 (33.4%) | 426 (88.9%) | 151 (31.7%) |
| **Mode of birth** | | | | |
| Spontaneous vaginal | 296 (83.6%) | 256 (84.8%) | 400 (83.5%) | 405 (84.4%) |
| Instrumental vaginal | 24 (6.8%) | 14 (4.6%) | 31 (6.5%) | 26 (5.4%) |
| Caesarean delivery | 34 (9.6%) | 32 (10.6%) | 48 (10.0%) | 49 (10.2%) |
| **Use of epidural anaesthesia** | 199 (56.2%) | 154 (51%) | 286 (59.7%) | 238 (49.6%) |
| **Maternal complications** | | | | |
| Perineal lacerations III-IV | 7 (2.0%) | 4 (1.3%) | 11 (2.3%) | 8 (1.7%) |
| Postpartum haemorrhage (>1000 ml) | 38 (10.7%) | 31 (10.3%) | 54 (11.3%) | 45 (9.4%) |
| Postpartum infection | 11 (3.1%) | 6 (2.0%) | 15 (3.1%) | 6 (1.3%) |
| Preeclampsia/gestational hypertension/eclampsia | 2 (0.6%) | 9 (3.0%) | 2 (0.4%) | 14 (2.9%) |
| **Perinatal complications** | | | | |
| Admittance to neonatal intensive care units (NICU) | 9 (2.5%) | 20 (6.6%) | 14 (2.9%) | 29 (6.0%) |
| Apgar score <7 at 5 min | 3/354 (0.8%) | 5/301 (1.7%) | 5/479 (1.0%) | 5/476 (1.1%) |
| Macrosomia (≥ 4500 g) | 19 (5.4%) | 30 (9.9%) | 2 (5.0%) | 46 (9.6%) |
| **Girls** | 165 (46.6%) | 136 (45.0%) | 222 (46.3%) | 213 (44.4%) |
| **Birth weight (g)** | | | | |
| Mean (SD) | 3811 (430) | 3905 (465) | 3816 (423) | 3897 (459) |
| Median (interquartile range) | 3800 (3530; 4066) | 3898 (3588; 4201) | 3800 (3540; 4084) | 3900 (3580; 4200) |



For peer review only - http://bmjopen.bmj.com/site/about/guidelines.xhtml

*Participant characteristics*

For participants responding to CEQ2, the baseline characteristics are presented in Table 2. In total, 90.1% of the women in the induction group had their labour induced, compared with 33.4% of the women in the expectant management group. The gestational age at delivery was on average 3 days longer in the expectant management group. The number of newborns admitted to neonatal intensive care units was 2.5% in the induction group and 6.6% in the expectant management group. The rates of spontaneous vaginal births were similar (83.6% versus 84.8% respectively).

*CEQ2 scores*

The results of the CEQ2 scores are presented for each domain, as well as the total CEQ score, in Table 3. There were no incomplete questionnaires. No significant differences in childbirth experience were identified in three of the four subscales (*Own capacity*, *Perceived safety* and *Professional support)* or in the total score in the CEQ2 between the two randomised groups. There was a significant difference in the subscale *Participation,* where women in the induction group scored slightly higher than women in the expectant management group (p=0.02), with a small effect size (0.19) (Table 3 and Figure 2).

**Table 3. Childbirth experience in the induction group and the expectant management group, according to the Childbirth Experience Questionnaire 2 (CEQ2)**

| | Induction group n=354 | Expectant management group n=302 | Effect size | p-value |
|---|---|---|---|---|
| **Own Capacity** | | | | |
| Mean (SD) | 2.8 (0.6) | 2.7 (0.6) | 0.16 | 0.09 |
| Median (interquartile range) | 2.9 (2.3-3.2) | 2.8 (2.3-3.2) | | |
| (95% CI for mean) | (2.7-2.9) | (2.6-2.8) | | |
| **Perceived Safety** | | | | |
| Mean (SD) | 3.3 (0.7) | 3.2 (0.8) | 0.17 | 0.06 |
| Median (interquartile range) | 3.5 (3.0-3.8) | 3.5 (2.7-3.8) | | |
| (95% CI for mean) | (3.3-3.4) | (3.1-3.3) | | |
| **Professional Support** | | | | |
| Mean (SD) | 3.6 (0.5) | 3.5 (0.6) | 0.09 | 0.38 |
| Median (interquartile range) | 3.8 (3.4-4.0) | 3.8 (3.4-4.0) | | |
| (95% CI for mean) | (3.5-3.6) | (3.4-3.6) | | |
| **Participation** | | | | |
| Mean (SD) | 3.6 (0.6) | 3.4 (0.7) | 0.19 | 0.02 |
| Median (interquartile range) | 4.0 (3.3-4.0) | 3.7 (3.0-4.0) | | |
| (95% CI for mean) | (3.5-3.6) | (3.4-3.5) | | |
| **Total CEQ** | | | | |
| Mean (SD) | 3.3 (0.5) | 3.2 (0.6) | 0.17 | 0.07 |



For peer review only - http://bmjopen.bmj.com/site/about/guidelines.xhtml

| | | |
|---|---|---|
| Median (interquartile range) | 3.5 (3.0-3.7) | 3.4 (2.9-3.7) |
| (95% CI for mean) | (3.3-3.4) | (3.2-3.3) |

p-values calculated with Mann-Whitney U-test.

## VAS

In total, 1802 women were included at the centres where CEQ2 was not distributed. One woman in the induction group withdrew her consent to participate before induction of labour. A total of 80% of women in the induction group and 82% women in the expectant management group rated their overall childbirth experience on a VAS (1-10) within 3 days of delivery (Supplementary material, Figure A).

### *Responders and non-responders*

The women responding to VAS had similar background characteristics to the women who did not answer VAS. Of women responding to VAS, 82.8% were born in Sweden, 61.6% had a university education, and 58.7% had their labour induced versus 79.9%, 62.0% and 60.5% of the non-responders (Supplementary material, Table B).

### *Participant characteristics*

In the induction group, 83.9% of women had their labour induced, compared with 33.5% of women in the expectant management group. Women in the expectant management group had a pregnancy on average 3 days longer than women in the induction group. There were similar modes of birth in the two groups, 83.2% women had a spontaneous vaginal birth in the induction group, and 82.4% women in the expectant management group (Supplementary Material, Table B).

### *VAS*

There was no significant difference between the induction group and the expectant management group in overall childbirth experience on VAS, or in the number of women rating their childbirth experience as positive (VAS 8-10) (Table 4).

**Table 4. Childbirth experience measured with VAS 1-10**

| | Induction group n=722 | Expectant management group n=735 | Effect size | p-value |
|---|---|---|---|---|
| **VAS** | | | | |
| Mean (SD) | 8.0 (1.2) | 8.1 (2.0) | -0.05 | 0.22 |
| Median (interquartile range) | 8 (7-10) | 9 (7-10) | | |
| (95% CI for Mean) | (7.9-8.2) | (8.0-8.3) | | |
| **VAS 8-10** | 497 (69%) | 528 (72%) | | 0.23 |

For continuous variables, p-values were calculated with the Mann-Whitney U-test.

For categorical variables, p-values were calculated with Pearson Chi-Square.



For peer review only - http://bmjopen.bmj.com/site/about/guidelines.xhtml

# DISCUSSION

The main finding in this study was that there were no significant differences in women's childbirth experiences between women randomised to induction of labour at 41 weeks and women randomised to expectant management and induction of labour at 42 weeks, in terms of the total CEQ2 score, three of four subscales (*Own Capacity, Perceived safety, Professional support)* or according to VAS. However, the women in the induction group scored slightly higher in the domain *Participation* compared with the women in the expectant management group. Women reported an overall positive childbirth experience.

Induction of labour has been reported to have a negative impact on women's childbirth experience.[15-17] However, this was not observed in our study, even though women had their labour induced to a larger extent in the induction group than in the expectant management group. The women had uncomplicated pregnancies and might have been more motivated for induction of labour as they were willing to participate in our study. Furthermore, induction can be perceived as a relief from feelings of discomfort in a prolonged pregnancy[17] and a way to meet the child sooner.[37] Another explanation for the results may be that there were no differences in caesarean deliveries or instrumental vaginal births between the two randomised groups,[25] factors that can have a negative impact on women's childbirth experience.[4 30 38]

None of the subscales *Own Capacity*, *Perceived Safety*, *Professional support* showed significant differences. Most women have expectations of their capacity to give birth and hope they will be able to use their own inherited physical and psychosocial capacities.[39] It is also reported that most women want a vaginal birth, focusing both on safety and psychosocial wellbeing.[39] A safe and supporting environment enables the woman to focus on techniques to manage the birth.[13]

Women in the induction group scored significantly higher in the subscale *Participation* than the expectant management group (3.6 versus 3.4, maximum score 4, p=0.02). In a systematic review of qualitative studies, Downe, et al. [39] found that most women want a physiological labour and birth. However, if interventions were needed, women wanted sufficient information and to be active in the decision-making to retain a sense of personal achievement and control.[39] This may be reflected in the domain *Participation* as the women who participated in the study may have been more motivated for induction of labour, and therefore those allocated to the induction group may have experienced being part of the decision-making to a higher degree than those randomised to the expectant management group (standard care).

The mean total CEQ2 score in the women in the induction group was 3.3, and 3.2 in the expectant management group. This is in agreement with earlier studies using the CEQ2. In the RCT by Walker et al., women at age 35 or older were randomised to either induction of labour at 39 weeks or expectant management until spontaneous onset or medical indication to induce labour (n=618).[22] The women in the induction group scored a total mean CEQ2 score of 3.03 versus 2.96 (p=0.12) in the expectant management group.[22] Also, women with vaginal delivery scored a mean total CEQ2 score of 3.3 in the CEQ2 validation study.[28]

In a retrospective cohort study from Sweden, including all women with a singleton pregnancy, using the VAS for assessment of overall birth experience, 69% (11 493/16 775) of women scored VAS 8-10 (mean 7.94).[15] These scores are similar to the scores in our study, where 69% in the induction group and 72% in the expectant management group scored 8-10 on VAS.



For peer review only - http://bmjopen.bmj.com/site/about/guidelines.xhtml

## Methodological considerations

By using CEQ2[28 30] to compare women's childbirth experiences, we chose an instrument with good psychometric properties.[40] Women could answer the CEQ2 in Swedish or English, which enabled more participants to fill in the questionnaire. The CEQ has been used in several studies in Sweden, and has been shown to have good internal and external validity.[41-44] The advantages of CEQ and CEQ2 are that they measure the multidimensional experience of labour and birth. However, there might be dimensions of the childbirth experience that are difficult to capture with a questionnaire.

A larger proportion of women answered the CEQ2 in the induction group (78%) than in the expectant management group (66%). This may be due to women not perceiving they were participating in a study when they were allocated to the expectant management group, which was standard care at the time. Furthermore, women with spontaneous onset of labour did not answer the questionnaire to the same extent as women whose labour was induced. This difference may also be related to that some women having spontaneous onset of labour perceived that they had not participated in the study. However, these differences in response rate between the randomisation groups were not seen for VAS. This might be explained by the fact that the VAS is used as part of the clinical routine including all women in the participating hospitals.

We added the VAS as an exploratory variable with the purpose of obtaining information on overall childbirth experience in the women at the 11 hospitals were the CEQ2 was not distributed. The reason for CEQ2 not was distributed at all participating centres were due to logistic reasons. The advantage of the VAS is that it is part of the clinical routine and therefore there might be less bias in the women's responses. On the other hand, it is a simplified and non-specific measure, and the women responded to it within three days after the birth, compared to the CEQ which was distributed 3 months after delivery and reflects four domains of childbirth. Hence, the childbirth experience may shift with time when the first wave of relief after the birth has settled. However, the VAS has been shown to correlate to women's overall childbirth experiences and is considered to be useful as a tool to screen for childbirth experience.[15 43 45]

A limitation of the study is that only 22% of eligible women participated in SWEPIS.[25] However, the baseline characteristics were similar for several background variables between the study population in SWEPIS and a Swedish background population of women with uncomplicated pregnancies. However, more women had completed a university education (64% vs. 55%) and were born in Sweden (83% vs. 73%) in SWEPIS compared to the background population.[25] Another limitation is that oral and written information was given by the midwives at a regular antenatal check-up at 40 weeks, but it is unclear how many women actually received the information. The caregiver's attitudes towards the study, as well as the influence of friends and family, might have affected the women's decision to participate.[46 47] Being part of SWEPIS gave the women a chance to have an induction of labour earlier than in routine care in Sweden, this was also expressed by women at randomisation. It is therefore probable that women participating in SWEPIS were more motivated for or positive to induction of labour than women who declined or chose not to contact the study team. If a specific intervention has negative associations, this may be a reason for not participating, and if it is considered favourable, a reason for participating.[48] Another reason for declining could have been that some women wanted a natural birth without interventions. This reason was expressed by women choosing not to be randomised in a RCT in the Netherlands, which aimed to compare the outcome between induction of labour at 41 weeks and expectant management until 42 weeks. In the same study, women who wanted induction reported lower quality of life (EQ-6D), than women preferring expectant management.[49]



For peer review only - http://bmjopen.bmj.com/site/about/guidelines.xhtml

In a qualitative study by Wessberg et al.,[20] women at 41 weeks expressed lack of information about late term pregnancy, showing the importance of clear and good information. This was also expressed in a study by Lou et al.[18] where some women requested more information about their options with late term pregnancy. Women included in SWEPIS, except in Region Stockholm, received an extra visit to a midwife that explained the procedure of induction of labour and thereby got extra detailed information about possible induction procedures. Therefore, the result of this study should be interpreted from this context.

# CONCLUSION

The main result of this study was that there were no significant differences in women's childbirth experiences between women randomised to induction of labour at 41 weeks and women randomised to expectant management and induction of labour at 42 weeks. However, women randomised to induction scored higher on the CEQ2 subscale *Participation* but the difference measured with effect size was small. Overall, women's ratings of their childbirth experience were high.

Further research is needed to describe women's experiences of induction of labour in a prolonged pregnancy, and how the health care system can meet these women's expectations and needs, in order to support them during pregnancy and childbirth.


## Acknowledgments

The authors wish to thank all the women who participated in the study and took their time to answer the questionnaire, and all the midwives and obstetricians at the participating centres. We thank and acknowledge the Swedish Network for National Clinical studies (SNAKS) for their assistance and support.


## Contributions

AW, AD, HH, UBW, VS and HE conceived and designed the study. HN, AW, HH, UBW, HF, JW, AKW and SS oversaw recruitment of study participants and collection of CEQ data at the local centres. UBW and HN did the data cleaning together with statisticians Mattias Molin and Per Ekman. HN compiled and analysed the CEQ2 data. Per Ekman did the analysis regarding VAS. HN wrote the first draft of the manuscript, which was then critically reviewed and revised by all co-authors (HN, AW, AD, HH, UBW, HF, JW, VS, IL, CB, AKW, SS and HE). All authors have approved the final version of the manuscript for submission. All authors have full access to all the data in the study and take responsibility for the integrity of the data and the accuracy of the data analysis. HE is the guarantor.

## Competing interests

None of the authors have any conflict of interest concerning this paper.


## Funding

This study was supported by the Swedish state under the agreement between the Swedish government and the county councils, the ALF-agreement (ALFGBG-440301, ALFGBG-718721,ALFGBG-70940, ALFGBG-426401), the Health Technology Centre at Sahlgrenska University Hospital, the Foundation of the Health and Medical care committee of the Region of Västra




For peer review only - http://bmjopen.bmj.com/site/about/guidelines.xhtml

Götaland, Sweden (VGFOUREG387351, VGFOUREG640891, VGFOUREG854081), Hjalmar Svensson Foundation, the foundation Mary von Sydow, born Wijk donation fund, Uppsala-Örebro regional research council (RFR-556711, RFR-736891), Region Örebro County research committee (OLL-715501), the ALF-agreement in Stockholm (ALF-561222,ALF-562222, ALF-563222), and Centre for Clinical Research Dalarna-Uppsala University, Sweden (CKFUU-417011). The funders had no role in the study design, data collection, data analysis, data interpretation, or writing of the report. The researchers were independent of the funders.

## Ethics approval

This study was approved by the Regional Ethics Board in Gothenburg, Sweden, in May 2014 (Dnr: 285–14) and later complementary applications (T 905-15, T 291-16, T 1180-16, T 330-17, T 1066-17, T 087-18, T 347-18, T 961-18, T 1110-18).

## Consent to participate

Informed written consent was obtained from all women prior to randomisation.

## Data availability statement

Data will be made available, from the corresponding author, upon reasonable request.

# REFERENCES

1. Larkin P, Begley CM, Devane D. Women's experiences of labour and birth: an evolutionary concept analysis. *Midwifery* 2009;25(2):e49-59. doi: 10.1016/j.midw.2007.07.010 [published Online First: 2007/11/13]
2. Lundgren I. Swedish women's experience of childbirth 2 years after birth. *Midwifery* 2005;21(4):346-54. doi: 10.1016/j.midw.2005.01.001 [published Online First: 2005/07/19]
3. Hildingsson I, Karlstrom A, Nystedt A. Women's experiences of induction of labour--findings from a Swedish regional study. *The Australian & New Zealand journal of obstetrics & gynaecology* 2011;51(2):151-7. doi: 10.1111/j.1479-828X.2010.01262.x [published Online First: 2011/04/07]
4. Bossano CM, Townsend KM, Walton AC, et al. The maternal childbirth experience more than a decade after delivery. *American journal of obstetrics and gynecology* 2017;217(3):342.e1-42.e8. doi: 10.1016/j.ajog.2017.04.027 [published Online First: 2017/04/30]
5. Simkin P. Just another day in a woman's life? Women's long-term perceptions of their first birth experience. Part I. *Birth (Berkeley, Calif)* 1991;18(4):203-10. [published Online First: 1991/12/01]
6. Simkin P. Just another day in a woman's life? Part II: Nature and consistency of women's long-term memories of their first birth experiences. *Birth (Berkeley, Calif)* 1992;19(2):64-81. [published Online First: 1992/06/01]
7. Olza I, Leahy-Warren P, Benyamini Y, et al. Women's psychological experiences of physiological childbirth: a meta-synthesis. *BMJ open* 2018;8(10):e020347. doi: 10.1136/bmjopen-2017-020347 [published Online First: 2018/10/21]
8. Bell AF, Andersson E. The birth experience and women's postnatal depression: A systematic review. *Midwifery* 2016;39:112-23. doi: 10.1016/j.midw.2016.04.014 [published Online First: 2016/06/21]
9. Nilsson C, Lundgren I. Women's lived experience of fear of childbirth. *Midwifery* 2009;25(2):e1-9. doi: 10.1016/j.midw.2007.01.017 [published Online First: 2007/05/22]



For peer review only - http://bmjopen.bmj.com/site/about/guidelines.xhtml

10. Dencker A, Nilsson C, Begley C, et al. Causes and outcomes in studies of fear of childbirth: A systematic review. *Women and birth : journal of the Australian College of Midwives* 2019;32(2):99-111. doi: 10.1016/j.wombi.2018.07.004 [published Online First: 2018/08/18]
11. Pang MW, Leung TN, Lau TK, et al. Impact of first childbirth on changes in women's preference for mode of delivery: follow-up of a longitudinal observational study. *Birth (Berkeley, Calif)* 2008;35(2):121-8. doi: 10.1111/j.1523-536X.2008.00225.x [published Online First: 2008/05/30]
12. Hollander MH, van Hastenberg E, van Dillen J, et al. Preventing traumatic childbirth experiences: 2192 women's perceptions and views. *Archives of women's mental health* 2017;20(4):515-23. doi: 10.1007/s00737-017-0729-6 [published Online First: 2017/05/30]
13. Karlstrom A, Nystedt A, Hildingsson I. The meaning of a very positive birth experience: focus groups discussions with women. *BMC pregnancy and childbirth* 2015;15:251. doi: 10.1186/s12884-015-0683-0 [published Online First: 2015/10/11]
14. Henderson J, Redshaw M. Women's experience of induction of labor: a mixed methods study. *Acta obstetricia et gynecologica Scandinavica* 2013;92(10):1159-67. doi: 10.1111/aogs.12211 [published Online First: 2013/07/03]
15. Falk M, Nelson M, Blomberg M. The impact of obstetric interventions and complications on women's satisfaction with childbirth a population based cohort study including 16,000 women. *BMC pregnancy and childbirth* 2019;19(1):494. doi: 10.1186/s12884-019-2633-8 [published Online First: 2019/12/13]
16. Lou S, Hvidman L, Uldbjerg N, et al. Women's experiences of postterm induction of labor: A systematic review of qualitative studies. *Birth (Berkeley, Calif)* 2018 doi: 10.1111/birt.12412 [published Online First: 2018/12/19]
17. Moore JE, Low LK, Titler MG, et al. Moving toward patient-centered care: Women's decisions, perceptions, and experiences of the induction of labor process. *Birth (Berkeley, Calif)* 2014;41(2):138-46. doi: 10.1111/birt.12080 [published Online First: 2014/04/08]
18. Lou S, Carstensen K, Hvidman L, et al. "I guess baby was just too comfy in there…": A qualitative study of women's experiences of elective late-term induction of labour. *Women and birth : journal of the Australian College of Midwives* 2020 doi: 10.1016/j.wombi.2020.03.012 [published Online First: 2020/05/15]
19. Wessberg A, Lundgren I, Elden H. Late-term pregnancy: Navigating in unknown waters - A hermeneutic study. *Women and birth : journal of the Australian College of Midwives* 2019 doi: 10.1016/j.wombi.2019.03.011 [published Online First: 2019/04/06]
20. Wessberg A, Lundgren I, Elden H. Being in limbo: Women's lived experiences of pregnancy at 41 weeks of gestation and beyond - A phenomenological study. *BMC pregnancy and childbirth* 2017;17(1):162. doi: 10.1186/s12884-017-1342-4 [published Online First: 2017/06/06]
21. Heimstad R, Romundstad PR, Hyett J, et al. Women's experiences and attitudes towards expectant management and induction of labor for post-term pregnancy. *Acta obstetricia et gynecologica Scandinavica* 2007;86(8):950-6. doi: 10.1080/00016340701416929 [published Online First: 2007/07/27]
22. Walker KF, Bugg GJ, Macpherson M, et al. Randomized Trial of Labor Induction in Women 35 Years of Age or Older. *N Engl J Med* 2016;374(9):813-22. doi: 10.1056/NEJMoa1509117 [published Online First: 2016/03/11]
23. Middleton P, Shepherd E, Crowther CA. Induction of labour for improving birth outcomes for women at or beyond term. *The Cochrane database of systematic reviews* 2018;5:Cd004945. doi: 10.1002/14651858.CD004945.pub4 [published Online First: 2018/05/10]
24. Alkmark M, Keulen JKJ, Kortekaas JC, et al. Induction of labour at 41 weeks or expectant management until 42 weeks: A systematic review and an individual participant data meta-analysis of randomised trials. *PLoS medicine* 2020;17(12):e1003436. doi: 10.1371/journal.pmed.1003436 [published Online First: 2020/12/09]



For peer review only - http://bmjopen.bmj.com/site/about/guidelines.xhtml

25. Wennerholm U-B, Saltvedt S, Wessberg A, et al. Induction of labour at 41 weeks versus expectant management and induction of labour at 42 weeks (SWEdish Post-term Induction Study, SWEPIS): multicentre, open label, randomised, superiority trial. *BMJ (Clinical research ed)* 2019;367:l6131. doi: 10.1136/bmj.l6131
26. Elden H, Hagberg H, Wessberg A, et al. Study protocol of SWEPIS a Swedish multicentre register based randomised controlled trial to compare induction of labour at 41 completed gestational weeks versus expectant management and induction at 42 completed gestational weeks. *BMC pregnancy and childbirth* 2016;16:49. doi: 10.1186/s12884-016-0836-9 [published Online First: 2016/03/10]
27. Stephansson O, Petersson K, Bjork C, et al. The Swedish Pregnancy Register - for quality of care improvement and research. *Acta obstetricia et gynecologica Scandinavica* 2018;97(4):466-76. doi: 10.1111/aogs.13266 [published Online First: 2017/11/25]
28. Dencker A, Bergqvist L, Berg M, et al. Measuring women's experiences of decision-making and aspects of midwifery support: a confirmatory factor analysis of the revised Childbirth Experience Questionnaire. *BMC pregnancy and childbirth* 2020;20(1):199. doi: 10.1186/s12884-020-02869-0 [published Online First: 2020/04/08]
29. Walker KF, Dencker A, Thornton JG. Childbirth experience questionnaire 2: Validating its use in the United Kingdom. *European journal of obstetrics & gynecology and reproductive biology: X* 2020;5:100097. doi: 10.1016/j.eurox.2019.100097 [published Online First: 2020/02/06]
30. Dencker A, Taft C, Bergqvist L, et al. Childbirth experience questionnaire (CEQ): development and evaluation of a multidimensional instrument. *BMC pregnancy and childbirth* 2010;10:81. doi: 10.1186/1471-2393-10-81 [published Online First: 2010/12/15]
31. Abbaspoor Z, Moghaddam-Banaem L, Ronaghi S, et al. Translation and Cultural Adaptation of the Childbirth Experience Questionnaire (CEQ) in Iran. *Iranian journal of nursing and midwifery research* 2019;24(4):296-300. doi: 10.4103/ijnmr.IJNMR_103_18 [published Online First: 2019/07/25]
32. Soriano-Vidal FJ, Oliver-Roig A, Cabrero-Garcia J, et al. The Spanish version of the Childbirth Experience Questionnaire (CEQ-E): reliability and validity assessment. *BMC pregnancy and childbirth* 2016;16(1):372. doi: 10.1186/s12884-016-1100-z [published Online First: 2016/11/26]
33. Walker KF, Wilson P, Bugg GJ, et al. Childbirth experience questionnaire: validating its use in the United Kingdom. *BMC pregnancy and childbirth* 2015;15:86. doi: 10.1186/s12884-015-0513-4 [published Online First: 2015/04/18]
34. Zhu X, Wang Y, Zhou H, et al. Adaptation of the Childbirth Experience Questionnaire (CEQ) in China: A multisite cross-sectional study. *PloS one* 2019;14(4):e0215373. doi: 10.1371/journal.pone.0215373 [published Online First: 2019/04/25]
35. Graviditetsregistrets Årsrapport 2018 (The Swedish Pregnancy Register yearly report 2018) [Available from: https://www.medscinet.com/GR/uploads/hemsida/dokumentarkiv/2018%20GR%20%C3%A5rsrapport_2.0.pdf.
36. Cohen J. A power primer. *Psychological bulletin* 1992;112(1):155.
37. Gatward H, Simpson M, Woodhart L, et al. Women's experiences of being induced for post-date pregnancy. *Women and birth : journal of the Australian College of Midwives* 2010;23(1):3-9. doi: 10.1016/j.wombi.2009.06.002 [published Online First: 2009/08/04]
38. Nystedt A, Hildingsson I. Women's and men's negative experience of child birth-A cross-sectional survey. *Women and birth : journal of the Australian College of Midwives* 2018;31(2):103-09. doi: 10.1016/j.wombi.2017.07.002 [published Online First: 2017/08/10]
39. Downe S, Finlayson K, Oladapo OT, et al. What matters to women during childbirth: A systematic qualitative review. *PloS one* 2018;13(4):e0194906. doi: 10.1371/journal.pone.0194906 [published Online First: 2018/04/18]



For peer review only - http://bmjopen.bmj.com/site/about/guidelines.xhtml

40. Nilver H, Begley C, Berg M. Measuring women's childbirth experiences: a systematic review for identification and analysis of validated instruments. *BMC pregnancy and childbirth* 2017;17(1):203. doi: 10.1186/s12884-017-1356-y [published Online First: 2017/07/01]
41. Bergqvist L, Dencker A, Taft C, et al. Women's experiences after early versus postponed oxytocin treatment of slow progress in first childbirth--a randomized controlled trial. *Sexual & reproductive healthcare : official journal of the Swedish Association of Midwives* 2012;3(2):61-5. doi: 10.1016/j.srhc.2012.03.003 [published Online First: 2012/05/15]
42. Hildingsson I, Rubertsson C. Childbirth experiences among women with fear of birth randomized to internet-based cognitive therapy or midwife counseling. *Journal of psychosomatic obstetrics and gynaecology* 2019:1-10. doi: 10.1080/0167482x.2019.1634047 [published Online First: 2019/06/28]
43. Turkmen S, Tjernstrom M, Dahmoun M, et al. Post-partum duration of satisfaction with childbirth. *The journal of obstetrics and gynaecology research* 2018;44(12):2166-73. doi: 10.1111/jog.13775 [published Online First: 2018/07/31]
44. Ulfsdottir H, Saltvedt S, Georgsson S. Women's experiences of waterbirth compared with conventional uncomplicated births. *Midwifery* 2019;79:102547. doi: 10.1016/j.midw.2019.102547 [published Online First: 2019/10/15]
45. Larsson C, Saltvedt S, Edman G, et al. Factors independently related to a negative birth experience in first-time mothers. *Sexual & reproductive healthcare : official journal of the Swedish Association of Midwives* 2011;2(2):83-9. doi: 10.1016/j.srhc.2010.11.003 [published Online First: 2011/03/29]
46. Monteiro TM, Katz L, Bento SF, et al. Reasons given by pregnant women for participating in a clinical trial aimed at preventing premature delivery: a qualitative analysis. *BMC pregnancy and childbirth* 2019;19(1):97. doi: 10.1186/s12884-019-2240-8 [published Online First: 2019/03/22]
47. van der Zande ISE, van der Graaf R, Hooft L, et al. Facilitators and barriers to pregnant women's participation in research: A systematic review. *Women and birth : journal of the Australian College of Midwives* 2018;31(5):350-61. doi: 10.1016/j.wombi.2017.12.009 [published Online First: 2018/01/27]
48. Oude Rengerink K, Logtenberg S, Hooft L, et al. Pregnant womens' concerns when invited to a randomized trial: a qualitative case control study. *BMC pregnancy and childbirth* 2015;15:207. doi: 10.1186/s12884-015-0641-x [published Online First: 2015/09/06]
49. Keulen JKJ, Nieuwkerk PT, Kortekaas JC, et al. What women want and why. Women's preferences for induction of labour or expectant management in late-term pregnancy. *Women and birth : journal of the Australian College of Midwives* 2020 doi: 10.1016/j.wombi.2020.03.010 [published Online First: 2020/05/24]

Figure Legends:

Figure 1. Flowchart of the women in the two randomised groups that responded to CEQ2 three months after birth.

Figure 2. Childbirth experience in the induction group and the expectant management group, according to the Childbirth Experience Questionnaire 2 (CEQ2). The line in the box refers to the median and the + refers to the mean.



For peer review only - http://bmjopen.bmj.com/site/about/guidelines.xhtml

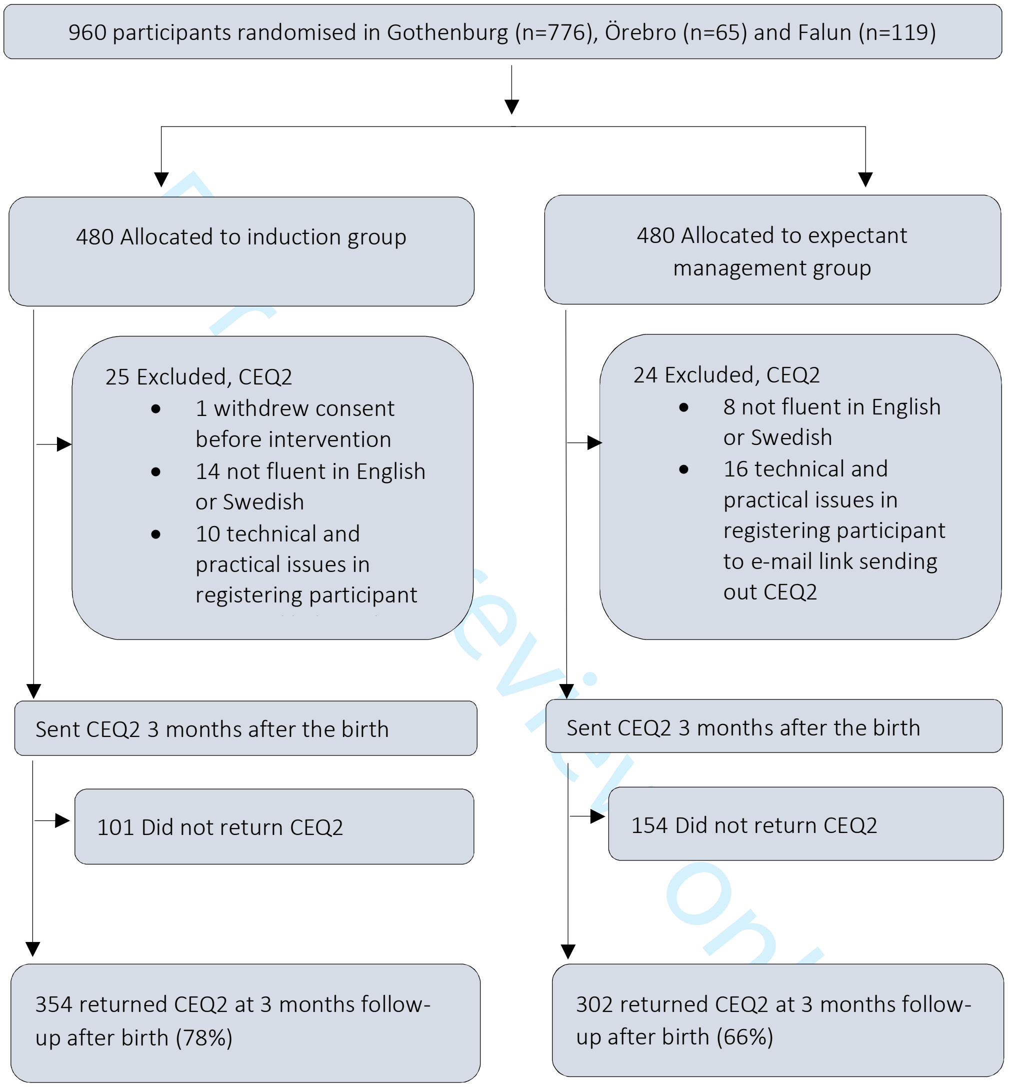
960 participants randomised in Gothenburg (n=776), Örebro (n=65) and Falun (n=119)
480 Allocated to induction group
480 Allocated to expectant management group
25 Excluded, CEQ2
1 withdrew consent before intervention
14 not fluent in English or Swedish
10 technical and practical issues in registering participant
24 Excluded, CEQ2
8 not fluent in English or Swedish
16 technical and practical issues in registering participant to e-mail link sending out CEQ2
Sent CEQ2 3 months after the birth
Sent CEQ2 3 months after the birth
101 Did not return CEQ2
154 Did not return CEQ2
354 returned CEQ2 at 3 months follow-up after birth (78%)
302 returned CEQ2 at 3 months follow-up after birth (66%)

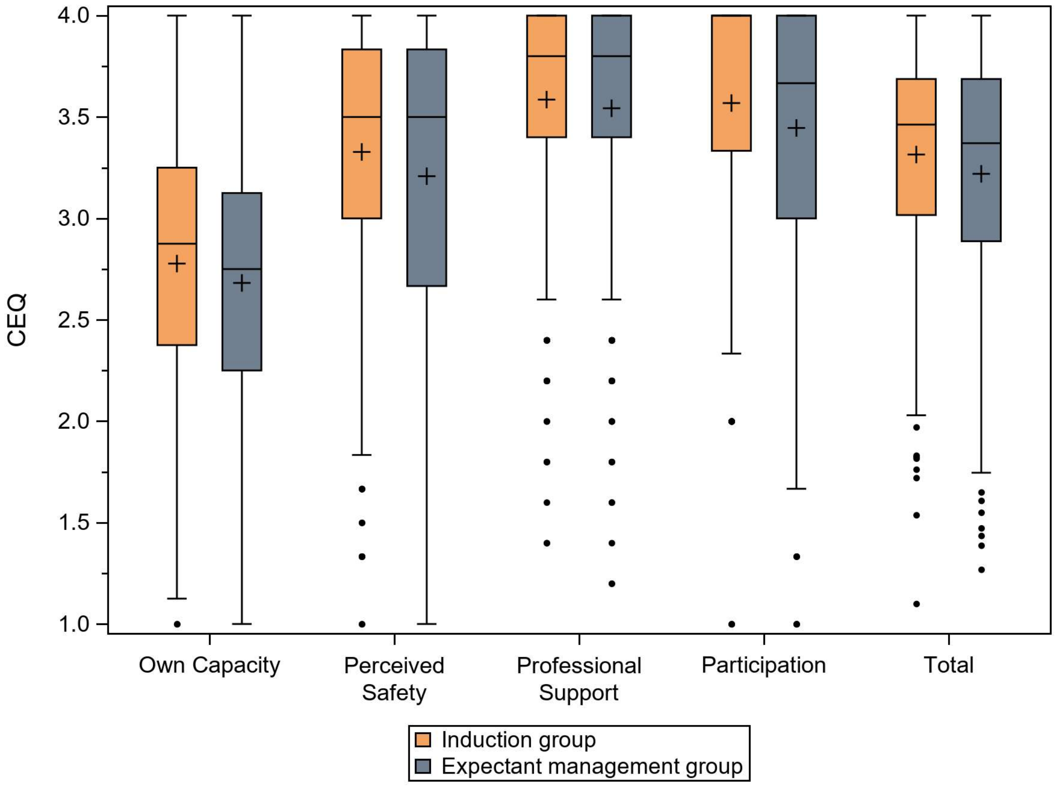
4.0
3.5
3.0
2.5
2.0
1.5
1.0
CEQ
Own Capacity
Perceived Safety
Professional Support
Participation
Total
Induction group
Expectant management group

Supplementary material, Figure A
Flowchart of participants in the two randomised groups rating their overall childbirth experience on a VAS (1-10) within 3 days of delivery

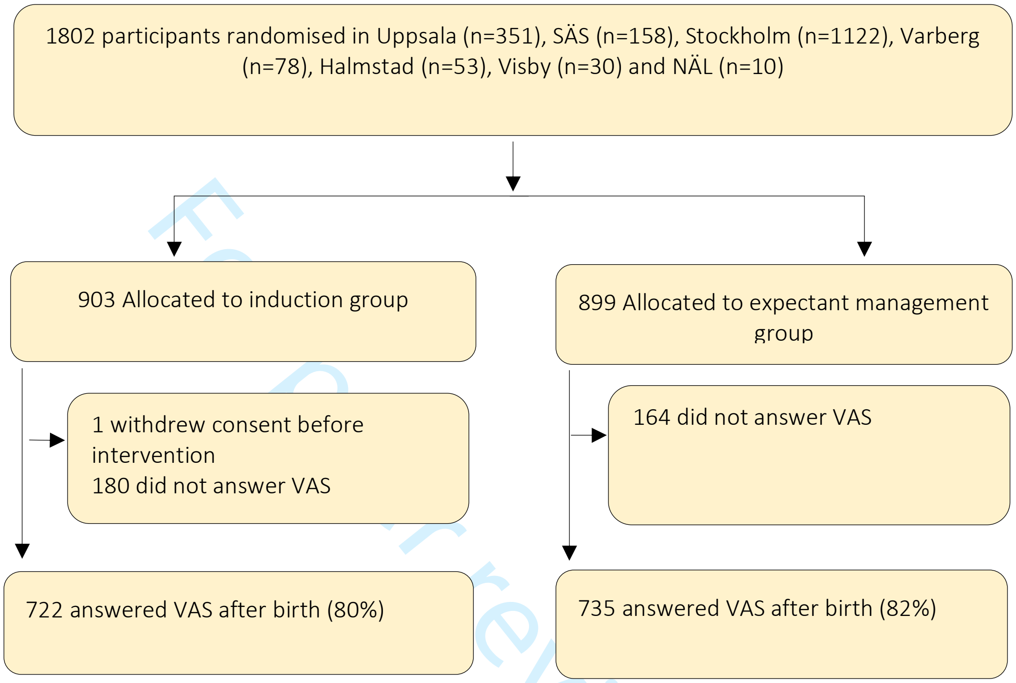


For peer review only

Supplementary material, Table A Baseline characteristics for participants not answering CEQ2

| Variables | Induction group n=125 | Expectant management group n=178 |
|---|---|---|
| Age at randomisation (years) | 30.7 (4.8) | 30.8 (4.8) |
| Mean (SD) | 30 (27; 34) | 30 (27; 35) |
| Median (interquartile range) | | |
| < 35 years | 99 (79.2%) | 139 (78.1%) |
| ≥ 35 years | 26 (20.8%) | 39 (21.9%) |
| Parity (includes stillborn or live births) | | |
| Primiparous | 73 (58.4%) | 88 (49.4%) |
| Multiparous | 52 (41.6%) | 90 (50.6%) |
| Smoking at first antenatal visit | | |
| No | 96/102 (94.1%) | 134/141 (95.0%) |
| Yes | 6/102 (5.9%) | 7/141 (5.0%) |
| BMI at first antenatal visit | 25.4 (5.0) | 25.7 (5.4) |
| Mean (SD) | 23.9 (21.8; 28.3) | 24.4 (22.0; 28.0) |
| Median (interquartile range) | n=108 | n=150 |
| Region of birth | | |
| Sweden | 84/115 (64.2%) | 123/167 (73.7%) |
| Other Nordic countries | 12/115 (10.4&) | 12/167 (7.2%) |
| Europe outside Nordic countries | 1/115 (1.0%) | 2/167 (1.2%) |
| Outside Europe | 18/115 (15.7%) | 12/167 (7.2%) |
| Highest education | | |
| Primary school ≤ 9 years | 5/102 (4.9%) | 6/155 (3.9%) |
| High school 9 to 12 years | 37/102 (36.2%) | 57/155 (36.8%) |
| University or corresponding | 60/102 (58.8%) | 92/155 (59.4%) |
| Gestational age at delivery (days) | 288.7 (1.6) | 291.1 (2.6) |
| Mean (SD) | 288 (288; 289) | 291 (289; 294) |
| Median (interquartile range) | | |
| Time from admittance to labour ward to delivery (hours) | 21.1 (13.9) | 12.8 (10.8) |
| Mean (SD) | 18.1 (10.5; 27.8) | 10.1 (5.1; 18.0) |
| Median (interquartile range) | | |
| Onset of birth process | | |
| Spontaneous | 18 (14.4%) | 126 (70.(%) |
| Scheduled caesarean delivery | 0 (0%) | 1 (0.6%) |
| Induction | 107 (85.6%) | 51 (28.7%) |
| Mode of birth | | |
| Spontaneous vaginal | 104 (83.2%) | 149 (83.7%) |
| Instrumental vaginal | 7 (5.6%) | 12 (6.7%) |
| Caesarean delivery | 14 (11.2%) | 17 (9.6%) |
| Use of epidural anaesthesia | 87 (69.6%) | 84 (47.2%) |
| Maternal complications | | |
| Perineal lacerations III-IV | 4 (3.2%) | 4 (2.2%) |
| Postpartum haemorrhage (>1000 ml) | 16 (12.8%) | 14 (7.9%) |
| Postpartum infection | 4 (3.2%) | 0 (0%) |
| Preeclampsia/gestational hypertension/eclampsia | 0 (0%) | 5 (2.8%) |
| Perinatal complications | | |
| Admittance to neonatal intensive care units (NICU) | 5 (4.0%) | 9 (5.1%) |
| Apgar score <7 at 5 min | 2/125 (1.6%) | 0/175 (0.0%) |
| Macrosomia (≥ 4500 g) | 5 (4.0%) | 16 (9.0%) |
| Girls | 57 (45.6%) | 77 (43.3%) |
| Birth weight (g) | 3829 (403) | 3882 (450) |
| Mean (SD) | 3815 (3547; 4102) | 3917 (3563; 4199) |
| Median (interquartile range) | | |

For peer review only - http://bmjopen.bmj.com/site/about/guidelines.xhtml

Supplementary material, Table B

Baseline characteristics for participants responding to a VAS (1-10) on overall childbirth experience, not responding to VAS and total VAS population of women participating in SWEPIS.

| | Participants responding to VAS | | Participants not responding to VAS | | Total VAS population | |
|---|---|---|---|---|---|---|
| Variables | Induction group n=722 | Expectant management group n=735 | Induction group n=180 | Expectant management group n=164 | Induction group n=902 | Expectant management group n=899 |
| Age at randomisation (years) | | | | | | |
| Mean (SD) | 31.3 (4.7) | 31.3 (4.7) | 31.5 (4.4) | 31.7 (4.3) | 31.3 (4.6) | 31.0 (4.6) |
| Median (interquartile range) | 31.2 (28; 35) | 31.2 (28; 35) | 31.5 (28; 34) | 31.5 (29; 35) | 31.2 (28; 34.6) | 30.9 (28; 34.2) |
| < 35 years | 567 (78.5%) | 567 (78.5%) | 141 (78.3%) | 127 (77.4%) | 708 (78.5%) | 716 (79.6%) |
| ≥ 35 years | 155 (21.5%) | 155 (21.5%) | 39 (21.7%) | 37 (22.6%) | 194 (21.5%) | 183 (20.4%) |
| Parity (includes stillborn or live births) | | | | | | |
| Primiparous | 402 (55.7%) | 407 (55.4%) | 91 (50.6%) | 94 (57.3%) | 493 (54.7%) | 501 (55.7%) |
| Multiparous | 320 (44.3%) | 328 (44.6%) | 89 (49.4%) | 70 (42.7%) | 402 (45.3%) | 398 (44.3%) |
| Smoking at first antenatal visit | | | | | | |
| No | 678/695 (97.6%) | 686/707 (97.0%) | 172/175 (98.3%) | 154/160 (96.3%) | 850/870 (97.7%) | 840/867 (96.9%) |
| Yes | 17/695 (2.0%) | 21/707 (2.0%) | 3/175 (1.7%) | 6/160 (3.8%) | 20/870 (2.3%) | 27/867 (3.1%) |
| BMI at first antenatal visit | | | | | | |
| Mean (SD) | 24.7 (4.4) | 25.1 (4.9) | 25.2 (5.1) | 24.6 (4.2) | 24.8 (4.6) | 25.0 (4.8) |
| Median (interquartile range) | 23.8 (21.7; 26.7) n=695 | 24 (21.6; 27.5) n=692 | 23.7 (22; 28) n=170 | 23.7 (22; 27) n=156 | 23.8 (21.7; 26.8) n=865 | 23.9 (21.6; 27.2) n=848 |
| Region of birth | | | | | | |
| Sweden | 562/683 (82.3%) | 581/697 (83.4%) | 130/161 (80.7%) | 120/152 (78.9%) | 692/844 (82.0%) | 701/849 (82.6%) |
| Other Nordic countries | 39/683 (5.7%) | 42/697 (6.0%) | 8/161 (5.0%) | 12/152 (7.9%) | 47/844 (5.6%) | 54/849 (6.4%) |
| Europe outside Nordic countries | 10/683 (1.5%) | 9/697 (1.3%) | 3/161 (1.9%) | 3/152 (2.0%) | 13/844 (1.5%) | 12/849 (1.4%) |
| Outside Europe | 72/683 (10.5%) | 65/697 (9.3%) | 20/161 (12.4%) | 17/152 (11.2%) | 92/844 (10.9%) | 82/849 (9.7%) |
| Highest education | | | | | | |

| | | | | | | |
|---|---|---|---|---|---|---|
| Primary school ≤ 9 years | 31/657 (4.7%) | 37/675 (5.4%) | 9/152 (5.9%) | 9/148 (6.1%) | 40/809 (4.9%) | 46/823 (5.8%) |
| High school 9 to 12 years | 207/657 (31.5%) | 237/675 (35.1%) | 51/152 (33.6%) | 45/148 (30.4%) | 258/809 (31.9%) | 282/823 (34.3%) |
| University or corresponding | 419/657 (63.8%) | 401/675 (59.4%) | 92/152 (60.5%) | 94/148 (63.5%) | 511/809 (63.2%) | 495/823 (60.1%) |
| Gestational age at delivery (days) | | | 289.2 (1.4) | 291.8 (2.7) | | |
| Mean (SD) | 288.9 (1.3) | 291.9 (2.6) | 289 (288; 290) | 292 (290; 295) | 289.0 (1.4) | 291.9 (2.7) |
| Median (interquartile range) | 289 (288; 289) | 292 (290; 294) | | | 289 (288; 289) | 292 (290; 294) |
| Time from admittance to labour ward to delivery (hours) | | | 21.2 (17.4) | 14.1 (12.3) | | |
| Mean (SD) | 19.7 (14.6) | 13.9 (12.9) | 17.1 (9; 29) | 10.1 (5; 20) | 20.0 (15.2) | 13.9 (12.8) |
| Median (interquartile range) | 15.9 (8.8; 27.5)<br>n=721 | 10.4 (4.6; 18.6)<br>n=734 | | | 15.9 (8.8; 27.9)<br>n=901 | 10.4 (4.6; 18.9)<br>n=898 |
| Onset of birth process | | | | | | |
| Spontaneous | 115 (15.9%) | 489 (66.5%) | 31 (17.2%) | 104(63.4%) | 146 (16.2%) | 592 (66.0%) |
| Scheduled caesarean delivery | 1 (0.1%) | 0 (0.0%) | 0 (0.0%) | 1 (0.6%) | 1 (0.1%) | 1/899 (0.1%) |
| Induction | 606 (83.9%) | 246 (33.5%) | 149 (82.8%) | 59 (36.0%) | 755 (83.7%) | 305 (33.9%) |
| Mode of birth | | | | | | |
| Spontaneous vaginal | 601 (83.2%) | 606 (82.4%) | 149 (82.8%) | 129 (78.7%) | 750 (83.1%) | 735 (81.8%) |
| Instrumental vaginal | 46 (6.4%) | 51 (6.9%) | 11 (6.1%) | 14 (8.5%) | 95 (10.5%) | 99 (11.0%) |
| Caesarean delivery | 75 (10.4%) | 78 (10.6%) | 20 (11.1%) | 21 (12.8%) | 57 (6.3%) | 65 (7.2%) |
| Use of epidural anaesthesia | 346 (47.9%) | 345 (46.9%) | 97 (53.9%) | 86 (52.4%) | 443 (49.1%) | 431 (47.9%) |
| Maternal complications | | | | | | |
| Perineal lacerations III-IV | 23 (3.2%) | 34 (4.6%) | 6 (3.3%) | 8 (4.9%) | 29/902 (3.2%) | 42/899 (4.7%) |
| Postpartum haemorrhage (>1000 ml) | 68 (9.4%) | 88 (12%) | 18 (10.0%) | 13 (7.9%) | 86/902 (9.5%) | 101/899 (11.2%) |
| Postpartum infection | 22 (3.0%) | 16 (2.2%) | 5 (2.8%) | 5 (3.0%) | 27/902 (3.0%) | 21/899 (2.3%) |
| Preeclampsia/gestational hypertension/eclampsia | 14 (1.9%) | 26 (3.5%) | 3 (1.7%) | 2 (1.2%) | 17/902 (1.9%) | 28/899 (3.1%) |
| Perinatal complications | | | | | | |
| Admittance to neonatal intensive care units (NICU) | 28 (3.9%) | 34 (4.6%) | 13 (7.2%) | 19 (11.7%) | 41/902 (4.5%) | 53/898 (5.9%) |



For peer review only - http://bmjopen.bmj.com/site/about/guidelines.xhtml

| | | | | | | |
|---|---|---|---|---|---|---|
| Macrosomia (≥ 4500 g) | 37 (5.1%) | 58 (7.9%) | 7 (3.9%) | 10 (6.1%) | 44/902 (4.9%) | 68/898 (7.6%) |
| **Girls** | 305 (42.2%) | 340 (46.3%) | 73/180 (40.6%) | 69/163 (42.3%) | 378/902 (41.9%) | 409/898 (45.5%) |
| **Birth weight (g)** | | | | | | |
| Mean (SD) | 3796 (406) | 3859 (424) | 3891 (373) | 3888 (418) | 3815 (401) | 3865 (423) |
| Median (interquartile range) | 3783 (3500; 4060) | 3845 (3565; 4120) | 3893 (3621; 4175) n=180 | 3900 (3610; 4190) n=163 | 3804 (3530; 4090) n=902 | 3850 (3570; 4128) n=898 |

For peer review only



For peer review only - http://bmjopen.bmj.com/site/about/guidelines.xhtml

# CONSORT 2010 checklist of information to include when reporting a randomised trial*

| Section/Topic | Item No | Checklist item | Reported on page No |
|---|---|---|---|
| **Title and abstract** | | | |
| | 1a | Identification as a randomised trial in the title | yes |
| | 1b | Structured summary of trial design, methods, results, and conclusions (for specific guidance see CONSORT for abstracts) | 2 |
| **Introduction** | | | |
| Background and objectives | 2a | Scientific background and explanation of rationale | 3 |
| | 2b | Specific objectives or hypotheses | 4 |
| **Methods** | | | |
| Trial design | 3a | Description of trial design (such as parallel, factorial) including allocation ratio | 4 |
| | 3b | Important changes to methods after trial commencement (such as eligibility criteria), with reasons | 3 |
| Participants | 4a | Eligibility criteria for participants | 4 |
| | 4b | Settings and locations where the data were collected | 4 |
| Interventions | 5 | The interventions for each group with sufficient details to allow replication, including how and when they were actually administered | 4 |
| Outcomes | 6a | Completely defined pre-specified primary and secondary outcome measures, including how and when they were assessed | 5 |
| | 6b | Any changes to trial outcomes after the trial commenced, with reasons | No changes |
| Sample size | 7a | How sample size was determined | This was a secondary outcome measure |
| | 7b | When applicable, explanation of any interim analyses and stopping guidelines | - |
| Randomisation: | | | |
| Sequence generation | 8a | Method used to generate the random allocation sequence | 4 |
| | 8b | Type of randomisation; details of any restriction (such as blocking and block size) | 4 |
| Allocation concealment mechanism | 9 | Mechanism used to implement the random allocation sequence (such as sequentially numbered containers), describing any steps taken to conceal the sequence until interventions were assigned | 4 |


For peer review only - http://bmjopen.bmj.com/site/about/guidelines.xhtml
For peer review only

| | | | |
|---|---|---|---|
| Implementation | 10 | Who generated the random allocation sequence, who enrolled participants, and who assigned participants to interventions | 4 |
| Blinding | 11a | If done, who was blinded after assignment to interventions (for example, participants, care providers, those assessing outcomes) and how | - |
| | 11b | If relevant, description of the similarity of interventions | - |
| Statistical methods | 12a | Statistical methods used to compare groups for primary and secondary outcomes | 6 |
| | 12b | Methods for additional analyses, such as subgroup analyses and adjusted analyses | 6 |
| **Results** | | | |
| Participant flow (a diagram is strongly recommended) | 13a | For each group, the numbers of participants who were randomly assigned, received intended treatment, and were analysed for the primary outcome | 6 |
| | 13b | For each group, losses and exclusions after randomisation, together with reasons | 7 |
| Recruitment | 14a | Dates defining the periods of recruitment and follow-up | 6 |
| | 14b | Why the trial ended or was stopped | 3 |
| Baseline data | 15 | A table showing baseline demographic and clinical characteristics for each group | 7 |
| Numbers analysed | 16 | For each group, number of participants (denominator) included in each analysis and whether the analysis was by original assigned groups | 7 |
| Outcomes and estimation | 17a | For each primary and secondary outcome, results for each group, and the estimated effect size and its precision (such as 95% confidence interval) | 8 |
| | 17b | For binary outcomes, presentation of both absolute and relative effect sizes is recommended | - |
| Ancillary analyses | 18 | Results of any other analyses performed, including subgroup analyses and adjusted analyses, distinguishing pre-specified from exploratory | - |
| Harms | 19 | All important harms or unintended effects in each group (for specific guidance see CONSORT for harms) | - |
| **Discussion** | | | |
| Limitations | 20 | Trial limitations, addressing sources of potential bias, imprecision, and, if relevant, multiplicity of analyses | 10-11 |
| Generalisability | 21 | Generalisability (external validity, applicability) of the trial findings | 11 |
| Interpretation | 22 | Interpretation consistent with results, balancing benefits and harms, and considering other relevant evidence | 9-10 |
| **Other information** | | | |
| Registration | 23 | Registration number and name of trial registry | 2 |
| Protocol | 24 | Where the full trial protocol can be accessed, if available | - |
| Funding | 25 | Sources of funding and other support (such as supply of drugs), role of funders | 12 |

For peer review only

*We strongly recommend reading this statement in conjunction with the CONSORT 2010 Explanation and Elaboration for important clarifications on all the items. If relevant, we also recommend reading CONSORT extensions for cluster randomised trials, non-inferiority and equivalence trials, non-pharmacological treatments, herbal interventions, and pragmatic trials. Additional extensions are forthcoming: for those and for up to date references relevant to this checklist, see www.consort-statement.org.

For peer review only


For peer review only - http://bmjopen.bmj.com/site/about/guidelines.xhtml
